# Brachyury engineers cardiac repair competent stem cells

**DOI:** 10.1002/sctm.20-0193

**Published:** 2020-10-24

**Authors:** Mark Li, Satsuki Yamada, Ao Shi, Raman Deep Singh, Tyler J. Rolland, Ryounghoon Jeon, Natalia Lopez, Lukas Shelerud, Andre Terzic, Atta Behfar

**Affiliations:** ^1^ Center for Regenerative Medicine, Van Cleve Cardiac Regenerative Medicine Program, Marriott Heart Disease Research Program, Department of Cardiovascular Medicine Mayo Clinic Rochester Minnesota USA; ^2^ Department of Molecular Pharmacology and Experimental Therapeutics Mayo Clinic Rochester Minnesota USA; ^3^ Division of Geriatric Medicine and Gerontology, Department of Medicine Mayo Clinic Rochester Minnesota USA; ^4^ Department of Biochemistry and Molecular Biology Mayo Clinic Rochester Minnesota USA; ^5^ Department of Clinical Genomics Mayo Clinic Rochester Minnesota USA; ^6^ Department of Physiology and Biomedical Engineering Mayo Clinic Rochester Minnesota USA

**Keywords:** cardiopoiesis, cardiopoietic stem cells, heart failure, myocardial infarction, regenerative therapy, RNA engineering

## Abstract

To optimize the regenerative proficiency of stem cells, a cardiopoietic protein‐based cocktail consisting of multiple growth factors has been developed and advanced into clinical trials for treatment of ischemic heart failure. Streamlining the inductors of cardiopoiesis would address the resource intensive nature of the current stem cell enhancement protocol. To this end, the microencapsulated‐modified‐mRNA (M^3^RNA) technique was here applied to introduce early cardiogenic genes into human adipose‐derived mesenchymal stem cells (AMSCs). A single mesodermal transcription factor, Brachyury, was sufficient to trigger high expression of cardiopoietic markers, Nkx2.5 and Mef2c. Engineered cardiopoietic stem cells (eCP) featured a transcriptome profile distinct from pre‐engineered AMSCs. In vitro, eCP demonstrated protective antioxidant capacity with enhanced superoxide dismutase expression and activity; a vasculogenic secretome driving angiogenic tube formation; and macrophage polarizing immunomodulatory properties. In vivo, in a murine model of myocardial infarction, intramyocardial delivery of eCP (600 000 cells per heart) improved cardiac performance and protected against decompensated heart failure. Thus, heart repair competent stem cells, armed with antioxidant, vasculogenic, and immunomodulatory traits, are here engineered through a protein‐independent single gene manipulation, expanding the available regenerative toolkit.

## Graphical abstract

Cardiopoietic stem cells are originally derived using a cocktail of cardiogenic growth factors. This study unveils the feasibility of achieving cardiopoiesis by a single gene (Brachyury) based induction of the human stem cell source. Engineered cardiopoietic stem cell progeny features pro‐reparative traits and rescues heart failure postinfarction, introducing a scalable platform for regenerative therapy.

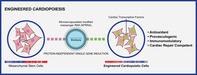

**Significance statement**

This article reports the engineering of cardiac repair competent stem cells using Brachyury, a mesodermal transcription factor. Brachyury transfection, achieved by the microencapsulated‐modified‐mRNA technique, induces naïve mesenchymal stem cells to adopt cardiopoietic fate endowed with cardioreparative proficiency. Evolving the traditional cardiopoiesis process, requiring multiple recombinant proteins, the newly streamlined single‐gene approach would simplify cell procurement, advancing clinical translation.

## 1 INTRODUCTION

Success in the management of myocardial infarction has paradoxically precipitated an epidemic of ischemic heart failure.^1^, ^2^, ^3^ In this context, reparative interventions, including delivery of stem cells, are explored as options to halt or reverse organ deterioration.^4^, ^5^, ^6^, ^7^ The safety and feasibility of adult stem cell therapy in patients with heart disease have been established, and mechanistic insights increasingly elucidated.^8^, ^9^, ^10^, ^11^, ^12^, ^13^, ^14^ Yet, there is a pressing need to advance these therapies in a fiscally responsible and logistically realistic way to adequately address the needs of a growing patient population.^15^

A case in point is the optimization of cardioreparative fitness in patient‐derived stem cells, proven achievable by coinstruction with cardiogenic growth factors.^16^, ^17^, ^18^, ^19^ This clinically implemented guided cardiopoiesis approach, employs however a complex regimen relying on multiple recombinant proteins.^20^, ^21^, ^22^ Used to derive cardiopoietic stem cells from a naïve mesenchymal stem cell source, this formulation remains resource intense and difficult to scale.^23^, ^24^ Protocol streamlining is thus warranted in order to facilitate further translational and clinical development.

Accordingly, this study probes essential inducer(s) of cardiopoiesis, and leverages a novel mRNA delivery system to engineer cardiac repair competency while limiting resource utilization in the production process. Human adipose‐derived mesenchymal stem cells (AMSCs) were here used as source material due to ease of harvest, malleability in culture, and repair potential.^25^, ^26^, ^27^ A microencapsulated‐modified‐messenger RNA (M^3^RNA) technology was implemented for targeted gene delivery to achieve a nonintegrating and viral‐free transfection.^28^, ^29^ This approach facilitated screening of known mesodermal and precardiac transcription factors to pinpoint best candidates for induction of cardiopoiesis. Ultimately, a single gene, Brachyury (T), was found sufficient to engineer the cardiopoietic phenotype, with maintained proficiency in rescuing the heart failure syndrome postinfarction, introducing a minimalistic approach for predelivery optimization of a regenerative cell product.

## 2 MATERIALS AND METHODS

### 2.1 Cell culture and transfection

Human AMSCs (Thermo Fisher, Waltham, Massachusetts; R7788115, Lot #1001002) were cultured with media composed of Advanced MEM (Thermo Fisher, 12492013), 1x Glutamax (Thermo Fisher, 35050079), 0.2% Normocin (InvivoGen, San Diego, California; ant‐nr‐2), units/mL heparin (National Drug Code 67457‐384‐31), and 2.5% PLTMax (MilliporeSigma, Burlington, Massachusetts; SCM141). Using the M^3^RNA mRNA delivery platform,^28^ AMSCs were transfected with seven distinct mRNAs, namely myocyte enhancer factor 2C (Mef2c; TriLink, San Diego, California; M14‐AH02B), NK2 homeobox 5 (Nkx2.5; TriLink, M14‐AH01B), mesoderm posterior bHLH transcription factor 1 (Mesp1; TriLink, M14‐AK01A), Brachyury (TriLink, M14‐AN01A), octamer‐binding transcription factor 4 (Oct4; TriLink, T1‐APH01A), GATA binding protein 4 (Gata4; TriLink, M14‐AH04A), and T‐box transcription factor 5 (Tbx5; TriLink, M14‐AH03A), individually or in various combinations. Media was changed every 2 days, and AMSCs at ≤7 passages were seeded at 80 000/well in a 6‐well plate (1 day prior to transfection), and washed in OptiMEM reduced serum media (Thermo Fisher, 31985070). A mixture of 3 μL of Lipofectamine Stem Transfection Reagent (Thermo Fisher, STEM00015) in 50 μL OptiMEM was added to the tested mRNA(s) (1.75 μg each in 50 μL OptiMEM), incubated for 9 minutes at 37°C, and dispensed to each well. Following 6 hours, 1.5 mL of regular MSC media was added per well.

### 2.2 Monitoring cardiopoiesis

Transfected AMSCs were washed with Phosphate Buffered Saline (PBS), fixed in 4% paraformaldehyde at room temperature for 20 minutes, permeabilized in PBS + 0.05% Triton‐X, quenched in 50 mM ammonium chloride for 20 minutes, and finally blocked (5% normal donkey serum, 0.2% Triton‐X in PBS) for 2 hours at room temperature prior to primary antibody incubation at 4°C overnight (Supporting Information Materials and Methods). Next day, cells were washed with PBS + 0.05% Triton‐X and incubated in secondary antibody for 2 hours at room temperature. To monitor cardiopoietic markers,^30^ cells were stained for Nkx2.5 (1:250; Santa Cruz, Dallas, Texas; sc‐376565) or Mef2C (1:1500; LifeSpan, Seattle, Washington; LS‐C356188‐100), colocalized with 4′,6‐diamidino‐2‐phenylindole (Thermo Fisher, D1306) and imaged using either Zeiss AxioObserver Z1 fluorescence microscope or Zeiss LSM 780 confocal microscope (Carl Zeiss AG, Oberkochen, Germany). Fluorescence images were converted to .tiff file for offline analysis (using Zen Blue, Carl Zeiss AG; ImageJ, NIH, Bethesda, Maryland). Expressions of Nkx2.5 and Mef2C were further confirmed by Western blot. Here, cytoplasmic and nuclear fraction of cell lysates were extracted using NE‐PER nuclear and cytoplasmic extraction reagent kit (Thermo Fisher, 78833). Proteins were run on 10% Criterion Tri‐HCl gels (BioRad, Hercules, California; 3450009) and transferred onto Odyssey Nitrocellulose membrane (Li‐Cor, Lincoln, Nebraska; 926‐31092). Total protein (Li‐Cor, 926‐11010) determination and final imaging were performed using the Li‐Cor Odyssey CLx imaging system and analyzed with the Empiria Studio software (Li‐Cor).

### 2.3 Transcriptome, RNA sequencing, and gene ontology analysis

RNA was extracted from transfected AMSCs, in biological triplicates using the RNeasy Plus Mini Kit (Qiagen, Hilden, Germany; 74134), and quantified with using the NanoDrop ND‐1000 spectrophotometer (Thermo Fisher). Time points included 0, 24, 48, 72, and 96 hours after transfection. RNA library preparations and sequencing reactions were conducted at GeneWiz (South Plainfield, New Jersey) using the NEBNext Ultra RNA Library Prep Kit for Illumina (NEB, Ipswich, Massachusetts). To this end, RNA samples were quantified using Qubit 2.0 Fluorometer (Life Technologies, Carlsbad, California) and RNA integrity was checked using Agilent 4200 TapeStation (Agilent Technologies, Santa Clara, California). Validated on the Agilent TapeStation and measured by Qubit 2.0 Fluorometer as well as by quantitative polymerase chain reaction (qPCR) (Kapa Biosystems, Wilmington, Massachusetts), the sequencing libraries were clustered on a single lane of a flow cell loaded on Illumina HiSeq 4000 (Illumina, San Diego, California). Samples were sequenced using a 2x 150 bp paired end configuration. Image analysis and base calling were conducted by the HiSeq control software. The gene hit counts table was used for downstream differential expression analysis. Using the edgeR R package (Bioconductor, Seattle, Washington), a comparison of gene expression between the groups of samples was performed. The quantile‐adjusted conditional maximum likelihood method (qCML)^31^ was used to generate *P* values and log2 fold changes. Genes with adjusted *P* values <.05 and absolute log2 fold changes >1 were called as differentially expressed genes. A Gene Ontology (GO) analysis was performed on the statistically significant set of genes by implementing the R package. Each group was subjected to functional annotation analysis under the “Biological Processes” category^32^, ^33^ using DAVID Bioinformatics Resources^34^ to determine significantly enriched gene.

### 2.4 Secretome

Conditioned media was collected from plates used to culture AMSCs with or without Brachyury transfection, and spun at 1500 g for 15 minutes. The supernatant was filtered using a 0.45‐μm filter to remove cell debris and then ultra‐centrifuged at 100 000*g* for 16 hours. The resulting supernatant was collected. The pellet was resuspended for downstream culture or analysis. Alternatively, media was centrifuged at 3000*g* for 30 minutes using Amicon Ultra‐15 centrifugal filter unit (MilliporeSigma, UFC901024).

### 2.5 Antioxidant capacity

AC16 human cardiomyocytes (MilliporeSigma, SCC109) were seeded onto a 96‐well plate at 8000 cells per well in 100 μL of culturing media. Next day, cells were treated with IncuCyte NucLight Rapid Red Reagent (1:500; Sartorius, Ann Arbor, Michigan; 4717) and Caspase‐3/7 Green (1:1000; Sartorius, 4440) in culture medium alone or plus OptiMEM, or plus AMSC‐derived conditioned media, or plus Brachyury transfected AMSC‐derived conditioned media. Concomitantly, 20 μM of LY83583 (Cayman chemical, Ann Arbor, Michigan; 70230) was added. Nucleus positive for Caspase‐3/7 were counted every 2 hours up to 24 hours (IncuCyte S3 Live‐Cell Analysis System, Sartorius). Superoxide dismutase (SOD) activity and total antioxidant capacity of conditioned media were measured using respective kits (Abcam, Cambridge, United Kingdom; ab65354 and ab65329). The concentration/ratio of conditioned medium ranged between 1.69 and 6.05 μg/μL, depending on the protein assay applied. Throughout protocols, the concentration was consistent among groups with equivalent volume of conditioned media loaded per well.

### 2.6 Tube formation assay and angiogenesis‐related protein profile

Matrigel basement membrane matrix (Corning, Corning, New York; 356231) was diluted in endothelial cell growth basal medium‐2 (Lonza, Basel, Switzerland; CC‐3156) and coated onto 96‐well plate at 37°C overnight. Then, 1 × 10^4^ green fluorescent protein (GFP)‐tagged human umbilical vein endothelial cells (HUVECs; Essen BioScience, Ann Arbor, Michigan; 4453) in 50 μL serum‐free EBM‐2 media was seeded per well 1 hour prior to treatment. Cells were incubated for 6 hours in 100 ng/mL vascular endothelial growth factor (VEGF; PromoCell, Heidelberg Germany; C‐64420), 10 μM LY83583 soluble guanylate cyclase inhibitor (CAS 91300‐60‐6), and conditioned media from AMSC with or without Brachyury transfection; fixed with 4% paraformaldehyde; and imaged on a Leica DMI6000 B microscope (Leica, Wetzlar, Germany). Images were exported and analyzed using AngioTool.^35^ Proteome profiling was performed using the human angiogenesis array kit (R&D, Minneapolis, Minnesota; ARY007) and analyzed using the Quick Spots tool from HLImage++ software (Western Vision, Salt Lake City, Utah).

### 2.7 Macrophage polarization

Bone marrow‐derived macrophages, harvested from the femurs of C57BL mice (Jackson Laboratory, Bar Harbor, Maine) and cultured for 7 days at 37°C in macrophage culturing media, namely RPMI (Gibco ‐ Thermo Fisher, Waltham, Massachusetts, 11875‐093), 1x Pen/strep (Gibco ‐ Thermo Fisher, 15140‐122), 10% Fetal Bovine Serum (FBS) (Gibco ‐ Thermo Fisher, 26140‐079), 50 μM β‐mercaptomethanol (MilliporeSigma, M7522), 50 ng/mL macrophage colony‐stimulating factor (MilliporeSigma, M6518), were pretreated with 50 ng/mL of lipopolysaccharides (MilliporeSigma, L6529) for 2 hours prior to adding PBS, conditioned media derived from AMSC with or without Brachyury transfection. After 24 hours incubation, cells were collected for real‐time quantitative polymerase chain reaction (RT‐qPCR). RT‐qPCR was performed using Quantitect SYBR Green RT‐PCR Kit (Qiagen, 204 243). The reaction was executed on ViiA 7 Real‐Time PCR System (Applied Biosystems, Foster City, California) with the following parameters: 30 minutes at 50°C, 15 minutes at 95°C, 40 cycles of 15 seconds at 94°C, 30 seconds at 60°C, 30 seconds at 72°C, then 15 seconds at 95°C. A complete list of primers is summarized in Supporting Information Materials and Methods.

### 2.8 Cell intervention for myocardial infarction

Eight‐ to 12‐week‐old athymic nude male mice (n = 14) underwent myocardial infarction surgery imposed by permanent ligation of the left anterior descending artery, followed by 1:1 randomization in the cell (n = 7) or vehicle control (n = 7) treatment groups. Stem cells were transfected with Brachyury, incubated for 72 hours (>90% viability) and epicardially delivered into six sites of the infarcted left ventricular (LV) wall (600 000 cells per heart in 15 μL Hanks' Balanced Salt Solution [HBSS; Thermo Fisher, 14025092]).^36^ Stem cell‐free vehicle treatment, using the same delivery protocol, consisted of delivering HBSS (15 μL per heart) injected into the LV. Systemic and cardiac parameters of heart failure postmyocardial infarction included survival, vital signs, treadmill performance (Columbus Instruments, Columbus, Ohio), oxygen consumption (Oxymax, Columbus Instruments), and LV structure and function measured by echocardiography (Vevo3100 with MX400, FUJIFILM VisualSonics, Toronto, Canada).^37^ Prospective echocardiography was scheduled during acute (2 days) and chronic (1 month) phases postinfarction to monitor randomized groups for equivalent initial infarction size and long‐term therapeutic outcomes, respectively.^38^, ^39^ Ejection fraction (EF) and mass of the LV were calculated from the two‐dimentional parasternal long‐axis view as described.^40^, ^41^, ^42^ To secure reproducibility and minimize bias, echocardiographic data were analyzed by a board‐certified cardiologist/sonographer in a blinded fashion. To assess cell proliferation, 5‐ethynyl‐2′‐deoxyuridine (EdU) was administered intraperitoneally (10 mg/kg in 200 μL saline, twice a week) for 1 month in infarcted animals with stem cell treatment (n = 5) followed by costaining of Edu and muscle‐specific desmin in cardiac tissue sections. Animal experiments were carried out in accordance with federal and institutional regulations. Humane handling of animals included prophylactic management of pain and distress using isoflurane anesthesia and analgesics. Animals demonstrating severe decompensated heart failure and/or a left ventricular ejection fraction (LVEF) of less than 10% were euthanized.

### 2.9 Histopathology

At end of follow‐up, noninfarcted, infarcted hearts with or without stem cell treatment were excised, and embedded in paraffin. Tissue samples were cut into 8 μm sections throughout the length of the heart, and stained with Masson's trichrome for collagen content and infarct perimeter/size quantification using ImageJ. For immunohistochemistry, antigen retrieval was performed in acidic buffer (R&D, CTS014). Slides, washed in PBS + 0.05% Triton‐X and quenched in 50 mM ammonium chloride for 30 minutes at room temperature, were incubated in the following steps; for 2 hours in blocking buffer (5% normal donkey serum, 0.2% Triton‐X, PBS) at room temperature, overnight in primary antibody at 4°C (Supporting Information Materials and Methods), and for 2 hours in secondary antibody at room temperature. Microscope images (Zeiss AxioObserver Z1 or LSM 780, Carl Zeiss AG) were exported as .tiff file using Zen Blue and analyzed using ImageJ.

### 2.10 Statistics

Unless otherwise stated, data are presented as mean ± SEM and a *P* value of less than .05 was considered significant. For in vitro studies, Student's *t* test, one‐way analysis of variance (ANOVA) followed by Bonferroni post hoc test, or Mann‐Whitney test were used where appropriate. For in vivo studies, data were analyzed in investigator‐blinded fashion. Two‐way repeated measures ANOVA, nonparametric Mann‐Whitney *U* test, or Fisher's exact test was used to compare stem cell vs vehicle control treatment (JMP Pro 14.1.0; SAS Institute, Cary, North Carolina).

## 3 RESULTS

### 3.1 Brachyury transfection induces cardiopoiesis

**FIGURE 1:**
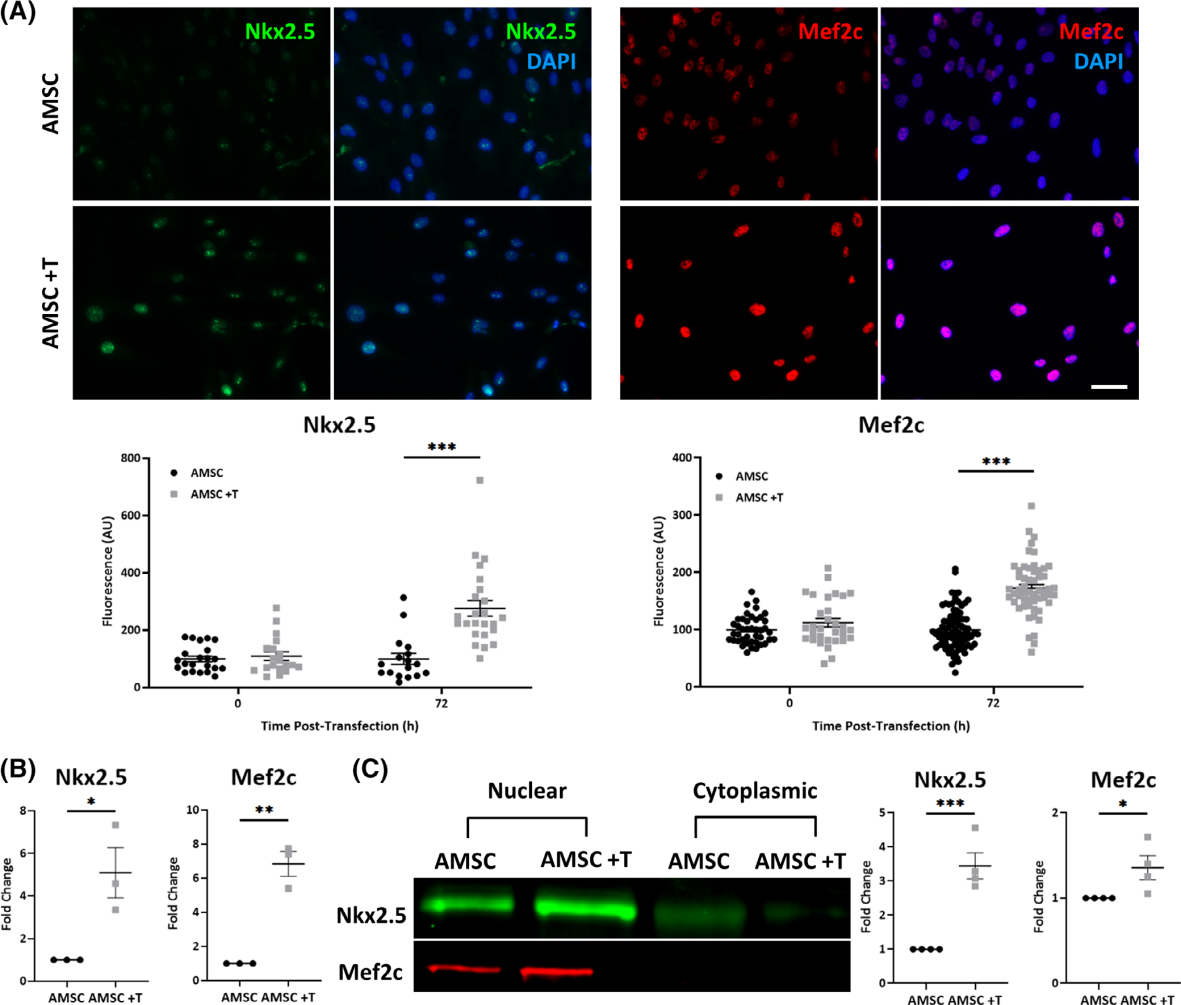
Brachyury (T) transfection provokes expression of cardiopoietic markers in human adipose‐derived mesenchymal stem cells (AMSCs). A, Immunofluorescent images demonstrate increased nuclear expressions of Nkx2.5 and Mef2c in AMSCs 72 hours post‐transfection with M^3^RNA encoding T (AMSC +T), compared to nontransfected AMSCs. Relative nuclear expressions of Nkx2.5 and Mef2c were compared based on fluorescence intensity normalized to nontransfected AMSCs. Numbers of biological replicates (n) were ≥17 for Nkx2.5 and ≥31 for Mef2c, per experiment condition. Scale bar = 20 μm. B, Increased levels of Nkx2.5 and Mef2c 72 hours post‐transfection were detected by real‐time quantitative polymerase chain reaction (n = 3). C, Western blot confirmed nuclear, not cytoplasmic, translocation of Nkx2.5 (n = 4) and Mef2c (n = 4) in AMSC +T at 72 hours post‐transfection. Protein expression was compared based on nuclear to cytoplasmic ratio of protein levels using (AMSC +T nuclear/AMSC +T cytoplasmic)/(AMSC nuclear/AMSC cytoplasmic) normalized to AMSC. Total protein stain was used as loading control. *P < .05; **P < .01; ***P < .001 with Student's *t* test

Permutations of mesodermal and precardiac transcription factors, delivered by the M^3^RNA‐based gene transfer system,^28^ were tested either in isolation or in combination^43^ for their respective aptitude to engender the cardiopoiesis program in human AMSCs. The efficiency of the M^3^RNA delivery system at 24 hours ranged from 63% to 94% with single, and 39% for double, transfection. Out of all tested combinations, single transfection of the mesodermal transcription factor Brachyury (T) achieved the highest expression of the cardiopoietic markers Nkx2.5 and Mef2c,^30^ as visualized by immunofluorescence (Table S1, Figure S1). M^3^RNA enabled expression of T peaked at 24 hours post‐transfection (Figure S2) followed by subsequent Nkx2.5 and Mef2c induction (Figure 1). At 72 hours post‐transfection, induction of cardiopoiesis was validated by nuclear expression of Nkx2.5 and Mef2c on immunocytochemistry (Figure 1A), RT‐qPCR (Figure 1B), and Western blot (Figure 1C). Accordingly, Brachyury transfection into human AMSC engineered cardiopoietic stem cells (eCP) fulfilling established phenotype release criteria.

### 3.2 Cardiopoietic transition unmasks prioritized biological processes

**FIGURE 2:**
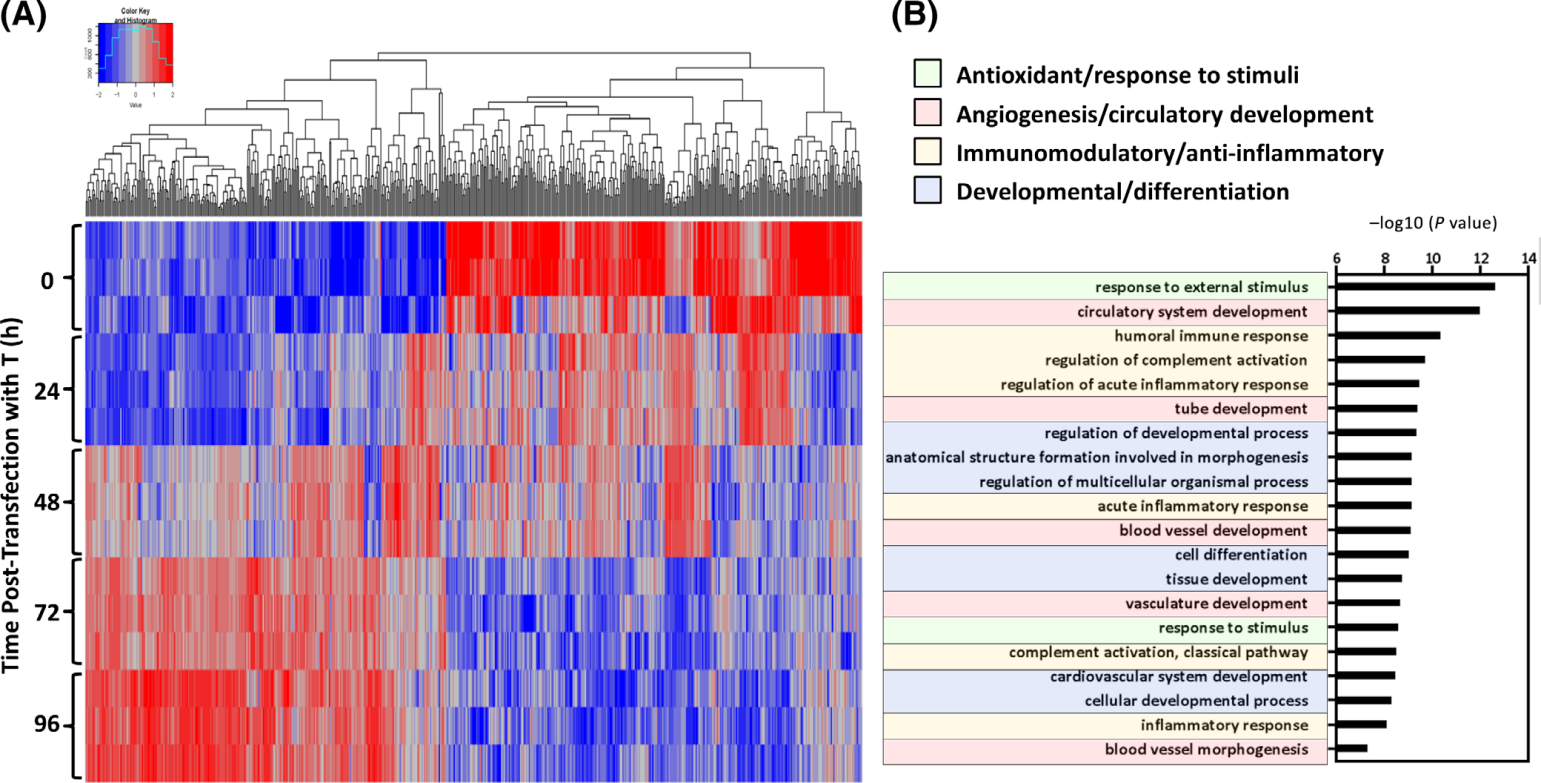
Brachyury (T) transforms the adipose‐derived mesenchymal stem cell transcriptome. A, Heat map was generated using RNA sequencing data from cell lysates at five time points; 0‐ (pre), 24‐, 48‐, 72‐, and 96‐hours after T‐transfection. Each time point consisted of biological triplicates (n = 3). Quantile‐adjusted conditional maximum likelihood method (qCML) was used for the initial bioinformatics analysis. B, Gene Ontology analysis of differentially expressed genes (upregulated at 72 hours vs 0 hour, based on *P* < .05 and log2‐fold change >1 cutoff) identified biological processes with enriched representation. T‐induced transcriptome prioritization included antioxidant/response to stimuli (green); angiogenesis/circulatory development (red); immunomodulatory/anti‐inflammatory response (yellow); cell, tissue, and organ development/differentiation (blue). These biological functions are all considered to be of potential benefit in cardiac repair

RNA sequencing, prospectively performed on cell lysates from time 0 and up to 96 hours following T transfection, revealed a progressive evolution of the gene expression pattern at global transcriptome level that stabilized by 72 hours post‐transfection (Figure 2A). Gene Ontology analysis, performed on differentially expressed genes upregulated at 72 hours (vs T naïve AMSC at 0 hour, Table S2), identified an enriched representation of several biological processes distinguishing nascent eCP from their unguided stem cell origin (Figure 2B). eCP prioritized biological processes were broadly categorized as antioxidant/response to stimuli, angiogenesis/circulatory development, immunomodulatory/anti‐inflammatory, and development/differentiation (Figure 2B), indicating a T induced imprint on the transcriptome underpinning eCP identity.

### 3.3 Antioxidant eCP capacity protects cardiomyocytes from oxidative stress

**FIGURE 3:**
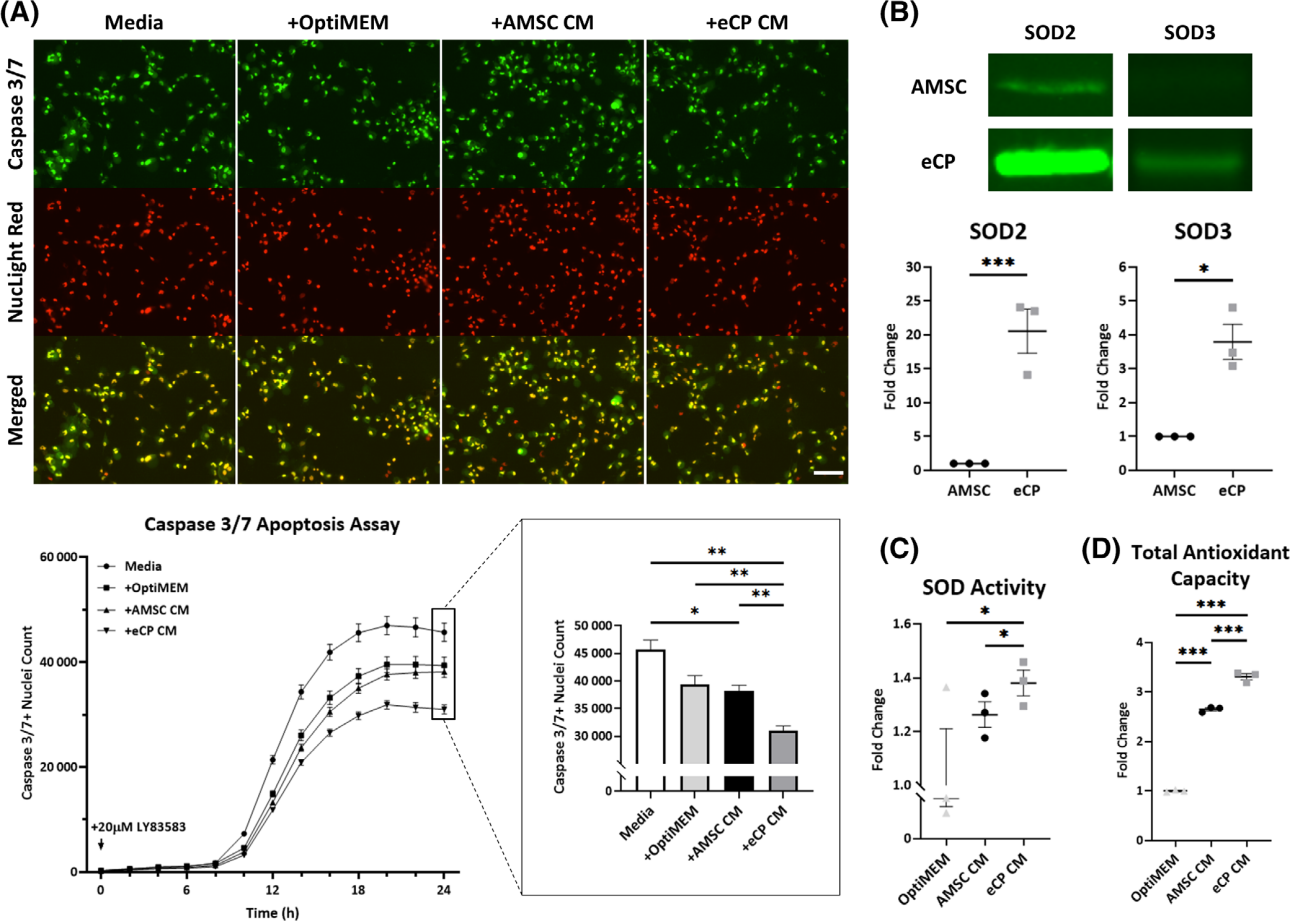
Engineered cardiopoietic stem cells (eCP) demonstrate elevated antioxidant capacity. A, Oxidative stress was induced in AC16 human cardiomyocytes using the free‐radical generator LY83583. Four treatment groups included; culture medium alone (Media), plus OptiMEM (+OptiMEM), plus adipose‐derived mesenchymal stem cell‐derived conditioned media (+AMSC CM), or plus Brachyury transfected engineered cardiopoietic stem cell‐derived conditioned media (+eCP CM). In each treatment group, 20 μL of conditioned media was added to 200 μL of regular media per well. Caspase 3/7 Green and NucLight Red identified apoptotic cells and cell nuclei, respectively (top panels). Significant decrease in apoptotic cardiomyocytes (bottom panels) indicated a protective effect of eCP conditioned media. Numbers of biological replicates (n) were nine per group at each time point. Scale bar = 100 μm. Antioxidant capacity was consistently detected as measured by: expression of antioxidant proteins SOD2 and SOD3 in total cell lysate (Western blot, n = 3; B); superoxide dismutase (SOD) activity in conditioned media (n = 3; C); and total antioxidant capacity in conditioned media (n = 3; D). OptiMEM served as control. *P < .05; **P < .01; ***P < .001 with one‐way ANOVA followed by a post hoc Bonferroni test (A, C, D) or Student's *t* test (B). ANOVA, analysis of variance

To test systems predicted functions intrinsic to the eCP phenotype, the free‐radical generator LY83583 was used to produce oxidative stress, and an apoptosis assay was used to probe the protective effects of eCP conditioned media (eCP CM). eCP CM significantly reduced apoptosis in stressed cultured cardiomyocytes, and showed superiority when compared to AMSC conditioned media (AMSC CM) or culture media controls (Figure 3A). eCP compared to AMSC displayed a significant increase in protein levels of superoxide dismutase 2 and 3 (SOD2/SOD3; Figure 3B), albeit not in SOD1, heme oxygenase 1 (HO1), or catalase (Figure S3). Beyond expression, eCP CM demonstrated increased SOD and total antioxidant activity compared to AMSC CM or culture media alone (Figure 3C,D). Thus, in vitro, eCP exhibit enhanced antioxidant protective capacity.

### 3.4 Angiogenic eCP capacity promotes vascular tube formation

**FIGURE 4:**
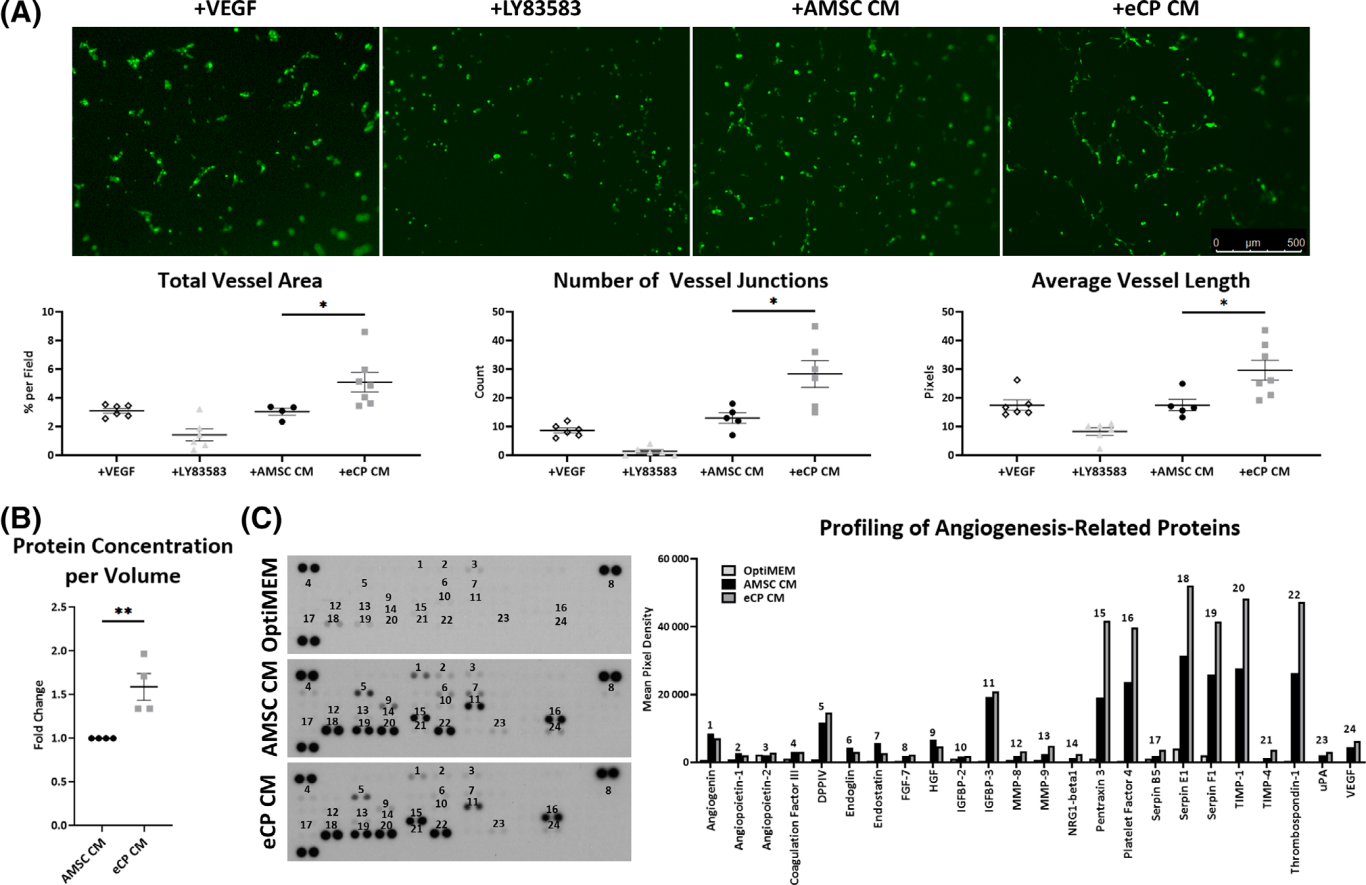
Angiogenicity and vasculogenic secretome. A, Green fluorescent protein (GFP)‐tagged human umbilical vein endothelial cells were treated with: vascular endothelial growth factor (+VEGF) as positive control, LY83583 as negative control (+LY83583), adipose‐derived mesenchymal stem cell conditioned media (+AMSC CM), or eCP conditioned media (+eCP CM). Conditioned media was extracted from the same starting volume (one confluent T75 flask, 10 mL volume) and 200 μL was added to 2 mL of media per well. Among four conditions, the +eCP CM group displayed superior vasculogenesis quantified by vessel area, junction, and length. Scale bar = 500 μm; *P < .05 with one‐way ANOVA followed by a post hoc Bonferroni test (biological replicates [n] ≥ 4 per group). B, eCP CM, compared to AMSC CM, contained a higher concentration of proteins. **P < .01 with Student's *t* test (n = 4 per group). C, Multiple angiogenesis‐related proteins were identified in eCP CM. After normalizing background signal and protein concentration of original blots (left panels) and their quantification, top 24 proteins from the 55‐protein array are depicted (n = 1 with technical duplicates; right panel). ANOVA, analysis of variance

Based on enriched pathways identified in Gene Ontology analysis, the angiogenic potential of eCP was further evaluated. In a matrigel tube formation assay, supplementing GFP‐tagged HUVECs with eCP CM increased total vessel area (70%), number of vessel junctions (more than doubled), and average vessel length (70%) compared to AMSC CM, VEGF (positive control) or LY83583^44^ (negative control) treatment (Figure 4A). A higher concentration of proteins was measured in eCP CM compared to AMSC CM (59%, Figure 4B), associated with increased quantity of angiogenic factors documented on an angiogenesis‐related proteome profiler array (Figure 4C). Thus, eCP are endowed with angiogenic capacity.

### 3.5 Enhanced immunomodulatory eCP property

**FIGURE 5:**
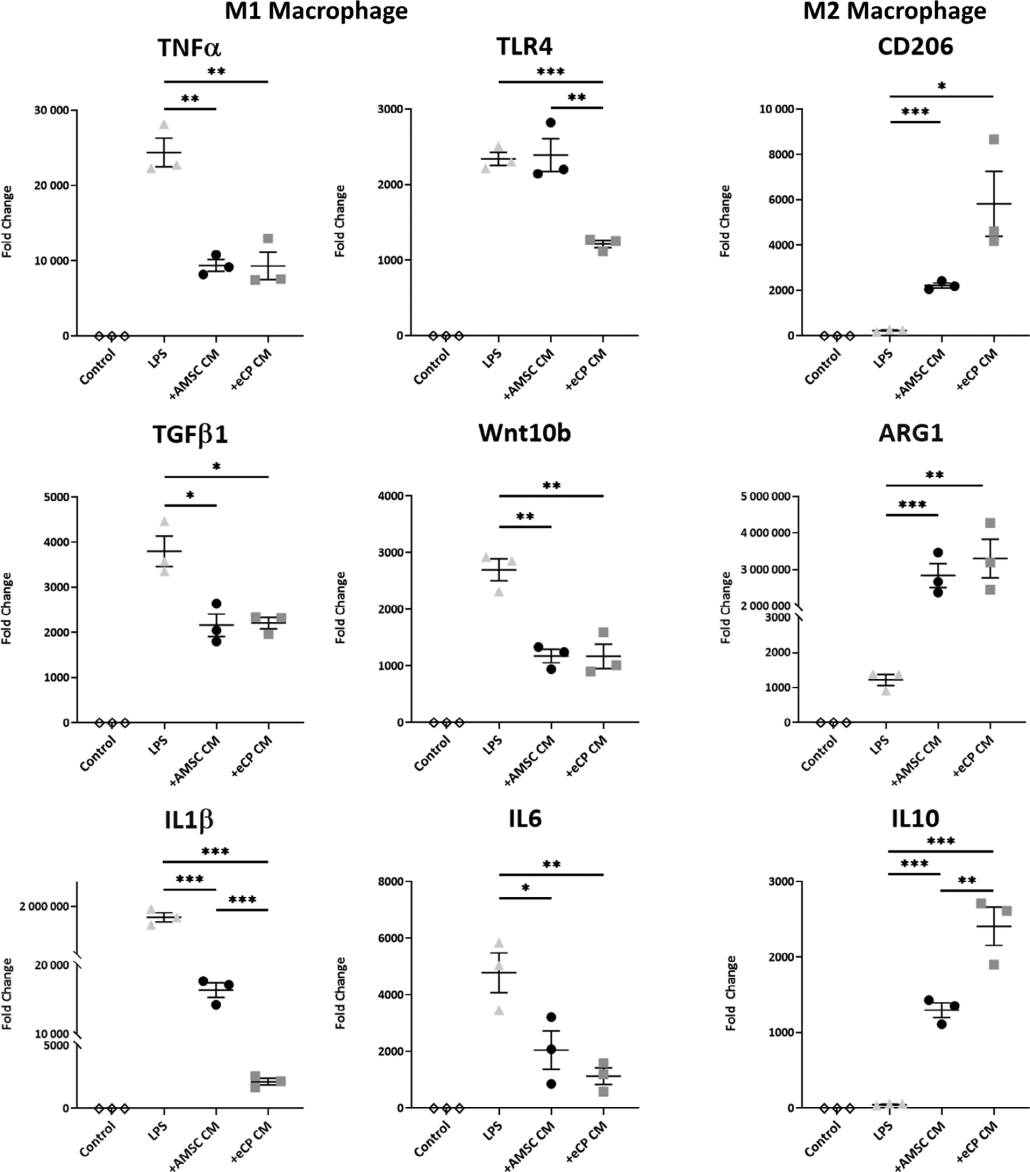
Enhanced immunomodulatory capacity. Macrophages, derived from mouse bone marrow, were cultured with: PBS (Control), lipopolysaccharides (LPS), adipose‐derived mesenchymal stem cell‐derived conditioned media (+AMSC CM), or eCP‐derived conditioned media (+eCP CM). Conditioned media was extracted from the same starting volume (one confluent T75 flask, 10 mL volume) and 200 μL was added to 2 mL of media per well. LPS‐induced inflammation, characterized by a surge in M1 macrophage markers, was reversed by +eCP CM treatment. Notably, anti‐inflammatory M2 macrophage markers were concomitantly activated by the +eCP CM treatment. Fold change was normalized to PBS (Control). *P < .05; **P < .01; ***P < .001 with one‐way ANOVA followed by a post hoc Bonferroni test (three biological replicates, with technical duplicates, per group). ANOVA, analysis of variance; PBS, phosphate buffered saline

The immunomodulatory property of eCP was evaluated using macrophage polarization assays. A range of known macrophage polarization markers was probed using RT‐qPCR (Figure 5) following treatment of mouse bone marrow‐derived macrophages. While both AMSC CM and eCP CM treatments reduced M1 markers (tumor necrosis factor α [TNFα]; transforming growth factor β1 [TGFβ1]; Wnt family member 10B [Wnt10b]; interleukin 1β [IL1β]; and interleukin 6 [IL6]), eCP CM showed significant reduction in Toll like receptor 4 (TLR4, *P* < .01) and IL1β (*P* < .001) transcript levels compared to AMSC CM, suggestive of a larger shift away from the M1 phenotype. Conversely, both treatments increased M2 expression, with a significant upregulation of interleukin 10 (IL10) in eCP CM (*P* < .01 vs AMSC CM; Figure 5, right bottom), indicative of a shift toward the M2 phenotype. As M1 and M2 macrophages are associated respectively with pro‐ and anti‐inflammatory activity,^45^, ^46^ M2 polarization suggests the heightened immunomodulatory effect of eCP.

### 3.6 Pro‐vasculogenic and antifibrotic effects in infarcted hearts

**FIGURE 6:**
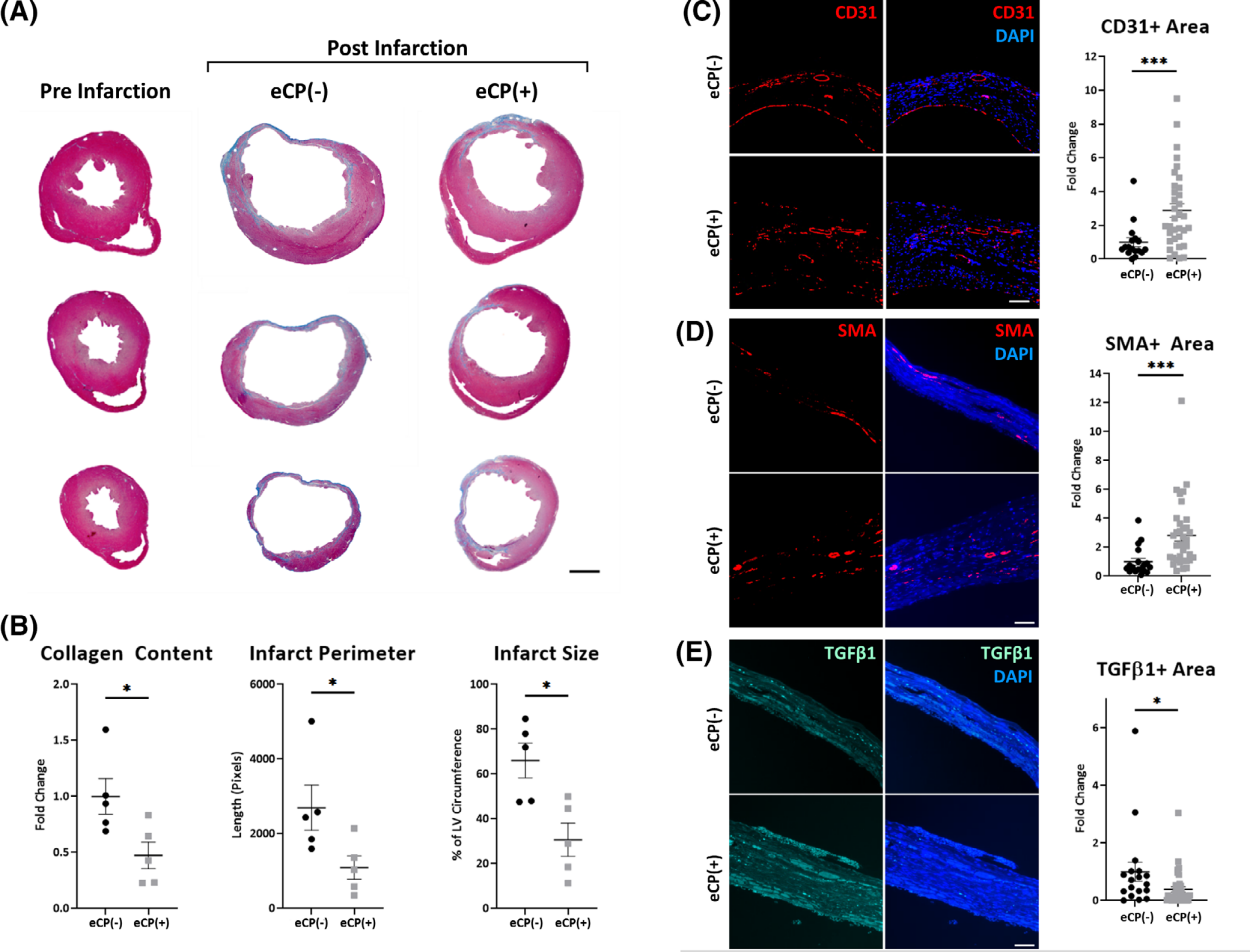
Tissue repair of infarcted hearts. A,B, Masson's trichrome staining of ventricular chamber dilatation and wall thinning, with collagen deposition (blue), in representative infarcted myocardial sections without cell treatment (eCP[−], 2 months postinfarction, right panels in A). Intramyocardial delivery of eCP cells (eCP[+], 600 000 cells per heart), following myocardial infarction, blunted cardiomyopathic features. Scale bar = 1 mm. Numbers of animals were five per group. C‐E, Immunohistochemistry of infarcted heart regions showed significant increase in CD31 and SMA staining in eCP treated mice, respective markers of endothelial and smooth muscle cells, and decrease in TGFβ1 staining, a marker of collagen synthesis and fibrotic processes. Fold change was normalized to eCP(−) infarcted heart counterparts. Scale bar = 100 μm; *P < .05; **P < .01; ***P < .001 with Mann‐Whitney test (≥17 left ventricular sections per group with five animals per group and ≥3 sections per animal). TGFβ1, transforming growth factor β1. SMA, smooth muscle actin

Based on in vitro findings, the in vivo potential of eCP was evaluated in a murine model of myocardial infarction. Gross examination of sectioned hearts at matching levels demonstrated structural and morphological differences between vehicle control and eCP treated groups using Masson's trichrome staining (Figure 6A). Quantitative assessment of heart sections revealed reduction in collagen content (*P* < .05) suggestive of decreased fibrosis and reduction in infarct perimeter (*P* < .05) and size (*P* < .05) following eCP treatment (Figure 6B). Significant increase in the endothelial marker CD31 (*P* < .001, Figure 6C) and smooth muscle actin (*P* < .001, Figure 6D) were noted in eCP treated infarcted heart regions. Concomitantly, TGFβ1, a marker of collagen synthesis and fibrotic processes,^47^ was reduced following eCP treatment (*P* < .05, Figure 6E). Of note, neither the human specific marker Ku80 nor Edu‐positive cardiomyocytes were noticeable within infarcted mouse hearts at 1 month post‐eCP therapy, suggesting a likely absence of a long term cell engraftment and new cardiomyocyte generation. Overall, eCP actions are consistent with an aptitude to promote vascularization and limit fibrosis in infarcted hearts.

### 3.7 Rescue of ischemic heart failure syndrome

**FIGURE 7:**
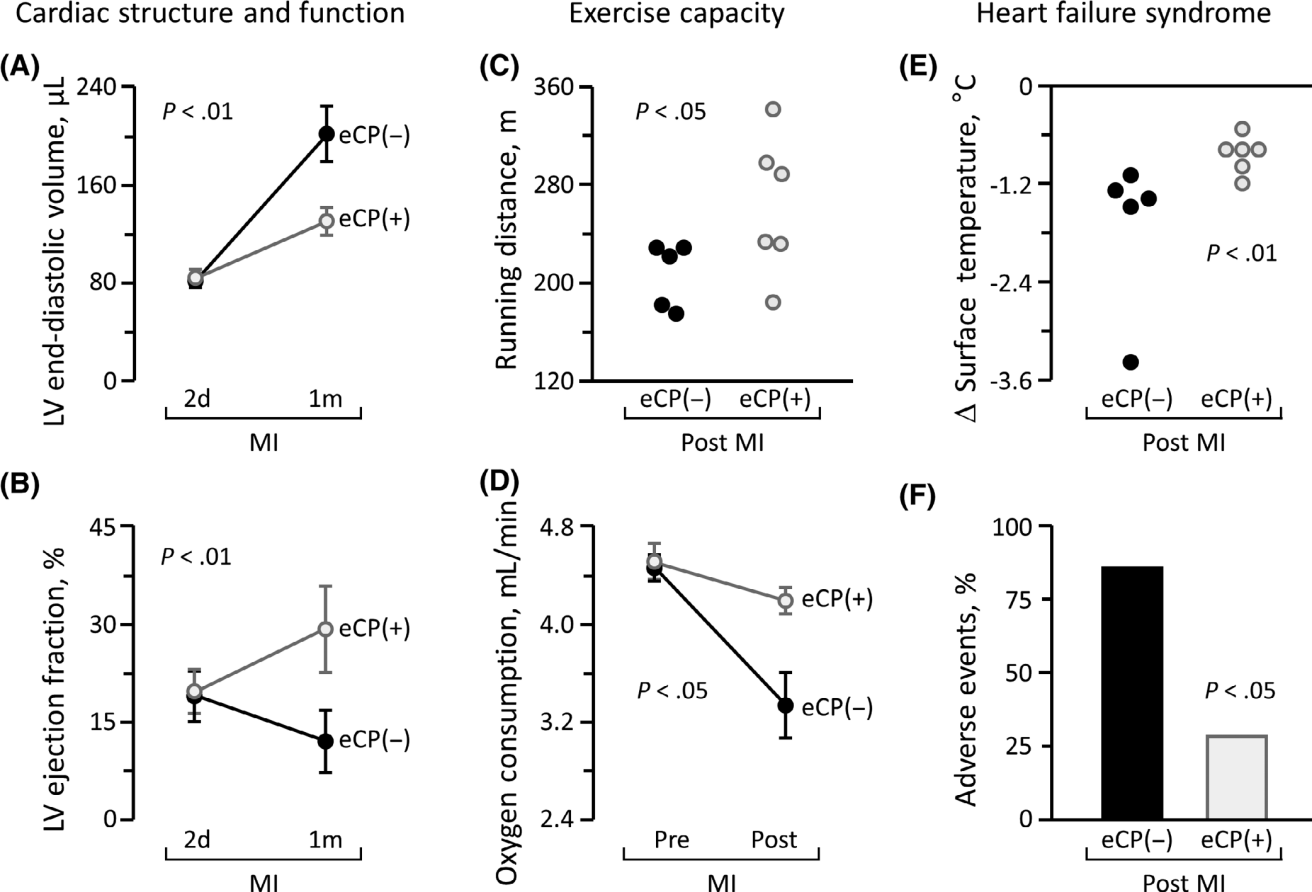
Therapeutic benefit in ischemic heart failure syndrome. Age and sex‐matched mice underwent myocardial infarction (MI) followed by randomization to vehicle control (eCP[−], n = 7) or eCP (eCP[+], 600 000 cells per heart, n = 7) treatment. Multiparametric validation of efficacy included monitoring of cardiac structure and function on echocardiography (A, B), exercise capacity on treadmill (C), whole body metabolism (D), vital sign (E), and cardiac events (F). Despite similar deterioration 2 days (2d) post‐MI induction, eCP(−) and eCP(+) cohorts diverged over time. Within 1 month (1m) of follow‐up, infarcted animals assigned to the eCP(−) group developed progressive cardiac dilatation and pump failure (A, B), reduced exercise performance (C, D), and poor circulation (E), resulting in a high incidence of adverse events (F). In contrast, eCP treatment prevented left ventricular (LV) enlargement (A), improved LV contractility (B), extended running distance (C), augmented maximum oxygen consumption (D), and maintained systemic circulation (E), protecting from severe decompensated heart failure (F). Animal numbers (n) with statistical analysis were; in (A, B, D), n = 5 in eCP(−) and n = 6 in eCP(+) with two‐way repeated measures ANOVA; in (C, E), n = 5 in eCP(−) and n = 6 in eCP(+) with Mann‐Whitney test; in (F), n = 7 in eCP(−) and n = 7 in eCP(+) with Fisher's exact test, respectively. The number declines in (A‐E), from the original cohorts (seven per group), is reflective of mortality during follow‐up. ANOVA, analysis of variance

The therapeutic impact on symptomatic heart failure postmyocardial infarction was prospectively evaluated in cohorts randomized (1:1) to receive vehicle control (eCP[−]) or eCP treatment (eCP[+]; Figure 7). Despite equivalent compromise in function and structure documented on echocardiography at 2 days (2d) after infarction, divergent outcomes at 1‐month (1m) between eCP(−) and eCP(+) hearts were observed (Figure 7A,B). Reflecting malignant progression, the eCP(−) cohort was characterized by exaggerated cardiac dilatation and reduced contractile performance within the 1m follow‐up. In contrast, eCP treatment counteracted LV dilatation and improved LVEF, indicating favorable action on infarction‐induced remodeling. Specifically, at 2d vs 1m postinfarction, the LV end‐diastolic volume more than doubled in the eCP(−) group, from 82 ± 5 μL to 202 ± 23 μL, while in the eCP(+) group, the increase was more moderate, from 84 ± 7 μL to 131 ± 11 μL (*P* < .01 vs eCP[−], Figure 7A). In parallel, LVEF continued to decline in eCP(−), from 19% ± 4% at 2d postinfarction to 12% ± 5% at 1m postinfarction. In contrast, in the eCP(+) group, LVEF recovered between 2d to 1m postinfarction from 20% ± 3% to 29% ± 7% (*P* < .01 vs eCP[−]; Figure 7B). Pathological gain in LV mass was minimized by eCP therapy (155 ± 12 mg in eCP[−] group vs 130 ± 4 mg in eCP[+] group, *P* < .05). eCP protection of LV geometry and pump function was associated with improved exercise capacity (Figure 7C,D), with rescue from heart failure symptoms and associated adverse events (death, severe decompensated heart failure, or LVEF <10%; Figure 7E,F). One month survivorship was documented in four out of seven animals without eCP cell treatment, and in six out seven with eCP cell treatment. At the 2 months follow‐up (Figure 7F), adverse events occurred in 6 eCP(−) infarcted animals (86%, 3 deaths, 3 severe decompensated heart failure with LVEF <10%), contrasting with 2 events in the eCP(+) group (29%, 2 deaths, 0 severe decompensated heart failure or LVEF<10%, *P* < .05 vs eCP[−]). Thus, eCP therapy shows benefit in ischemic heart failure.

## 4 DISCUSSION

Derivation of stem cells enhanced for functional organ repair is an area of intense investigation. Within the clinically tested regenerative armamentarium, cardiopoietic guidance exemplifies the promise of an optimization process, yet exposes the complexity of scalable implementation. In this study, heart repair proficient stem cells were engineered in a streamlined manner leveraging a single gene transfection approach, thereby overcoming the taxing nature of the currently available protein cocktail‐based cardiopoiesis. Brachyury, a mesoderm transcription factor critical for heart development, was here sufficient to convert a naïve mesenchymal stem cell into a cardiopoietic phenotype. Equipped with tissue protective traits, transplantation of engineered human cardiopoietic stem cells was found beneficial in myocardial infarction. Thus, recombinant protein‐independent induction of cardiopoiesis is achievable through single gene engineering to yield therapeutically competent cells validated for translational readiness.

RNA‐based strategies, free of genomic integration, have an accelerated developmental path and have reached FDA‐sanctioned clinical trials.^48^, ^49^ Accordingly, to ensure safe lineage commitment, mesodermal or precardiac transcription factors were here expressed using a M^3^RNA strategy, established for transient induction of target genes into the infarcted heart.^28^ Among pro‐cardiogenic transcription factors tested, individually or in combination, Brachyury was most proficient in inducing the expression and nuclear translocation of Nkx2.5 and Mef2c, qualifying cardiopoietic cell release.^18^, ^23^ Indeed, a recognized master regulator of mesoderm development and patterning, Brachyury is primordial in earliest cardiovascular differentiation programs controlling downstream cardiopoietic transcription factors.^50^, ^51^, ^52^ Multiple transfections with M^3^RNAs did not yield higher expression of cardiopoietic markers. This may be in part due to increased cytotoxicity associated with cationic liposome‐based gene delivery methods^48^, ^53^ or negative feedback from downstream effectors of cardiogenesis.^54^ A single transfection of M^3^RNA‐facilitated Brachyury afforded a means to achieve the cardiopoietic state within 72 hours, suggesting an expeditious production strategy.^8^

The dynamic pathobiology of progressive heart failure postinfarction is multifaceted, involving a cascade encompassing ischemic damage, inflammation, and tissue death.^55^ Stem cell‐based therapies, in principle, may readjudicate the broadly compromised molecular substrate.^18^, ^42^ Transformation of the mesenchymal stem cell transcriptome, following M^3^RNA‐encoded Brachyury transfection, boosted the antioxidant capacity, angiogenic potency, and immunomodulatory aptitude of derived cardiopoietic progeny. Acquisition of enhanced cardioreparative features translated into disease rescue with engineered cardiopoietic stem cell therapeutic efficacy validated for the ischemic heart failure syndrome. Intramyocardial delivery of Brachyury‐engineered cardiopoietic stem cells achieved cardiac and systemic functional rescue with vasculogenic and antifibrotic tissue repair in the apparent absence of long‐term cell engraftment and de novo cardiomyogenesis. Accordingly, the present study suggests myocardial salvage through paracrine action of engineered cardiopoietic stem cells in the setting of acute myocardial infarction.

## 5 CONCLUSION

The concept of stem cell guidance to achieve improved clinical outcome, previously implemented using a demanding recombinant protein cocktail‐based protocol,^56^, ^57^, ^58^, ^59^, ^60^ has been streamlined in a protein‐free manner by leveraging RNA‐based single gene delivery. Engineered cardiopoiesis enabled derivation of a cardioreparative biotherapeutic for ischemic heart disease. Prototyped in this study with cardiopoietic stem cells, this scalable platform has the potential to ease production and facilitate the advancement of regenerative therapies.

## CONFLICT OF INTEREST

S.Y., A.T., and A.B. are coinventors on regenerative sciences related intellectual property disclosed to Mayo Clinic. Previously, Mayo Clinic has administered research grants from Celyad. Mayo Clinic, A.T. and A.B. have interests in Rion LLC. The other authors declared no potential conflicts of interest.

## AUTHOR CONTRIBUTIONS

M.L., A.S.: conception and design, collection and assembly of data, data analysis and interpretation, manuscript writing, final approval of manuscript; S.Y.: conception and design, financial support, collection and assembly of data, data analysis and interpretation, manuscript writing, final approval of manuscript; R.D.S., T.J.R.: conception and design, collection and assembly of data, data analysis and interpretation, final approval of manuscript; R.J., N.L., L.S.: collection and assembly of data, data analysis and interpretation, final approval of manuscript; A.T., A.B.: conception and design, financial support, administrative support, data analysis and interpretation, manuscript writing, final approval of manuscript.

## Supporting information


**Appendix** **S1**: Supporting Information

**Figure S1** Probing M^3^RNA‐based transcription factor delivery on cardiopoietic markers induction. A total of 10 transcription factor permutations, either single or in combination, were tested (see also the Table S1). Expression of cardiopoietic markers, Nkx2.5 and Mef2, was measured at 72 hours post‐transfection using human AMSC as the starting cell source. Representative immunofluorescent images, from four independent experiments, illustrate that single gene transfection with Brachyury (T) induces Nkx2.5 and Mef2 (second panels from the top in the screening experiment 1 and bottom panels in the screening experiments 2 and 3). AMSC, adipose‐derived mesenchymal stem cells; Gata4, GATA binding protein 4; Mef2c, myocyte enhancer factor 2C; Mesp1, mesoderm posterior bHLH transcription factor 1; M^3^RNA, microencapsulated‐modified‐mRNA; Nkx2.5, NK2 homeobox 5; Oct4, octamer‐binding transcription factor 4; Tbx5, T‐box transcription factor 5. Scale bars indicate 20 μm for the screening experiments 1‐3, and 50 μm for the screening experiment 4.

**Figure S2** Time course of Brachyury‐induced cardiopoiesis. Representative immunofluorescent images depict increased expression of Brachyury (T) in AMSC transfected using the M^3^RNA delivery system with a peak level recorded at 24 hours post‐transfection. Induction of cardiopoietic markers, Nkx2.5 and Mef2c, was detected by 72 hours. Fluorescence was normalized to non‐transfected AMSCs for each time point. Numbers of biological replicates (n) were ≥ 17 for T, ≥ 15 for Nkx2.5, and ≥ 25 for Mef2c, per time point. *, *P* < .05; **, *P* < .01; ***, *P* < .001 with Student's *t*‐test. Scale bar = 100 μm

**Figure S3** Time course of antioxidant protein expression by Western blot in cell lysates of Brachyury (T) transfected AMSCs. Transfection of AMSCs with T increased levels of superoxide dismutase 2 and 3 (SOD2 (n = 3), SOD3 (n = 3)), but not of heme oxygenase 1 (HO1, n = 3), catalase (n = 2) or SOD1 (n = 2), at the 72‐hours time point. **, *P* < .01; ***, *P* < .001 with one‐way ANOVA followed by a post‐hoc Bonferroni test.

**Supplemental Table 1** 

**Supplemental Table 2** 

## Data Availability

The authors declare that all data supporting the findings of this study are available within the article and its Supporting Information files, or from the corresponding author upon reasonable request.
