# Supplementary material for: Brachyury engineers cardiac repair competent stem cells
Source: Stem Cells Transl Med. 2020 Oct 24;10(3):385–97. doi: 10.1002/sctm.20-0193 (PMC7900595; doi:10.1002/sctm.20-0193)
Supplement: Supplementary file 1 — Appendix S1: Supporting Information [file SCT3-10-385-s001.docx]

**Supplemental Materials and Methods**

**Antibodies Used for Immunocytochemistry, Immunohistochemistry, and Western Blotting**

|  | Target | Dilution | Company | Location | Catalog Number |
| --- | --- | --- | --- | --- | --- |
| Immunocytochemistry | Brachyury | 1:200 | R&D | Minneapolis, MN | AF2085 |
|  | Gata4 | 1:250 | R&D | Minneapolis, MN | AF2606 |
|  | Mef2c | 1:1500 | LifeSpan | Seattle, WA | LS-C356188-100 |
|  | Mesp1 | 1:400 | Abcam | Cambridge, UK | ab129387 |
|  | Nkx2.5 | 1:250 | Santa Cruz | Dallas, TX | Sc-376565 |
|  | Oct4 | 1:400 | Cell Signaling Technology | Danvers, MA | 2750 |
|  | Tbx5 | 1:500 | Abcam | Cambridge, UK | ab123665 |
| Immunohistochemistry | CD31 | 1:50 | R&D | Minneapolis, MN | AF3628 |
|  | SMA | 1:1000 | Abcam | Cambridge, UK | ab7817 |
|  | TGFβ1 | 1:100 | Abcam | Cambridge, UK | ab92486 |
|  | Ku80 | 1:250 | Abcam | Cambridge, UK | ab80592 |
| Western Blotting | Catalase | 1:1000 | R&D | Minneapolis, MN | MAB3398 |
|  | HO1 | 1:1000 | MilliporeSigma | Burlington, MA | 374090 |
|  | Mef2c | 1:1000 | Invitrogen | Carlsbad, CA | MA5-17119 |
|  | Nkx2.5 | 1:1000 | Novus Biologicals | Littleton, CO | NBP1-31558 |
|  | SOD1 | 1:1000 | Cell Signaling Technology | Danvers, MA | 2770S |
|  | SOD2 | 1:1000 | R&D | Minneapolis, MN | MAB3419 |
|  | SOD3 | 1:1000 | R&D | Minneapolis, MN | MAB34201 |

Gata4, GATA binding protein 4; HO1, heme oxygenase 1; Mef2c, myocyte enhancer factor 2C; Mesp1, mesoderm posterior bHLH transcription factor 1; Nkx2.5, NK2 homeobox 5; Oct4, octamer-binding transcription factor 4; SMA, smooth muscle actin; SOD1, SOD2, SOD3, superoxide dismutase 1, 2, 3; Tbx5, T-box transcription factor 5; TGFβ1, transforming growth factor β 1.

**Primers used for RT-qPCR**

| Species | Target | Sense | Antisense |
| --- | --- | --- | --- |
| Human | ACTB | GGA TCA GCA AGC AGG AGT ATG | AGA AAG GGT GTA ACG CAA CTA A |
|  | GAPDH | GGT GTG AAC CAT GAG AAG TAT GA | GAG TCC TTC CAC GAT ACC AAA G |
|  | MEF2C | GCA GAA GCC AAG AGA CCT TAT | GGC AGG CTA GCA TCC TTT AT |
|  | NKX2.5 | CGC ACC CAC CCG TAT TTA T | GGG TCA ACG CAC TCT CTT T |
| Mouse | ACTB | GAG GTA TCC TGA CCC TGA AGT A | CAC ACG CAG CTC ATT GTA GA |
|  | ARG1 | ACA GCA AAG CAG ACA GAA CTA | GAA AGG AAC TGC TGG GAT ACA |
|  | CD206 | GGA ATC AAG GGC ACA GAG TTA | TTC CAT CTG CTC CAC AAT CC |
|  | IL10 | TTG AAT TCC CTG GGT GAG AAG | TCC ACT GCC TTG CTC TTA TTT |
|  | IL1β | ATG GGC AAC CAC TTA CCT ATT T | GTT CTA GAG AGT GCT GCC TAA TG |
|  | IL6 | GTC TGT AGC TCA TTC TGC TCT G | GAA GGC AAC TGG ATG GAA GT |
|  | TGFβ1 | GGT GGT ATA CTG AGA CAC CTT G | CCC AAG GAA AGG TAG GTG ATA G |
|  | TLR4 | AGT ATC GAG AGG CTC AGG TAT AG | TAC AGG ATG CAG GAC AAG TAA TC |
|  | TNFα | CTA CCT TGT TGC CTC CTC TTT | GAG CAG AGG TTC AGT GAT GTA G |
|  | WNT10B | CCT CGG GTG ACA ATA ATG AGA G | ACA ACT GAA CGG AAG GAG AAG |

ACTB, actin beta; ARG1, arginase 1; CD206, macrophage mannose receptor cluster of differentiation 20; GAPDH, glyceraldehyde 3-phosphate dehydrogenase; IL1β, interleukin 1 β; IL6, interleukin 6; IL10, interleukin 10; MEF2C, myocyte enhancer factor 2C; NKX2.5, NK2 homeobox 5; TGFβ1, transforming growth factor β 1; TLR4, Toll like receptor 4; TNFα, tumor necrosis factor α; WNT10B, Wnt family member 10B.
