# Supplementary figures and images for: Brachyury engineers cardiac repair competent stem cells
Source: Stem Cells Transl Med. 2020 Oct 24;10(3):385–97. doi: 10.1002/sctm.20-0193 (PMC7900595; doi:10.1002/sctm.20-0193)

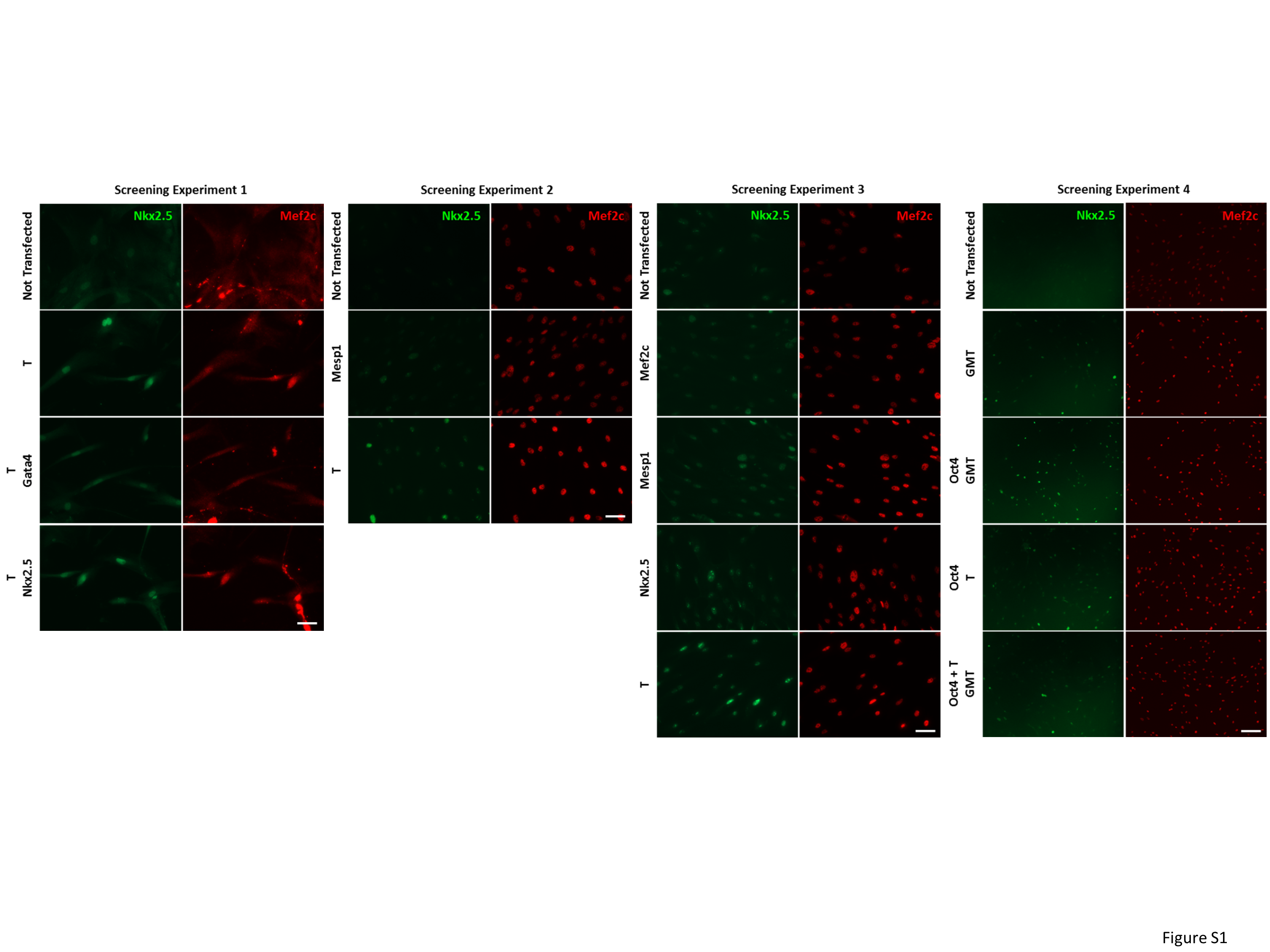

Supplement: Supplementary file 2 — Figure S1 Probing M3RNA‐based transcription factor delivery on cardiopoietic markers induction. A total of 10 transcription factor permutations, either single or in combination, were tested (see also the Table S1). Expression of cardiopoietic markers, Nkx2.5 and Mef2, was measured at 72 hours post‐transfection using human AMSC as the starting cell source. Representative immunofluorescent images, from four independent experiments, illustrate that single gene transfection with Brachyury (T) induces Nkx2.5 and Mef2 (second panels from the top in the screening experiment 1 and bottom panels in the screening experiments 2 and 3). AMSC, adipose‐derived mesenchymal stem cells; Gata4, GATA binding protein 4; Mef2c, myocyte enhancer factor 2C; Mesp1, mesoderm posterior bHLH transcription factor 1; M3RNA, microencapsulated‐modified‐mRNA; Nkx2.5, NK2 homeobox 5; Oct4, octamer‐binding transcription factor 4; Tbx5, T‐box transcription factor 5. Scale bars indicate 20 μm for the screening experiments 1‐3, and 50 μm for the screening experiment 4. [file SCT3-10-385-s002.TIF]

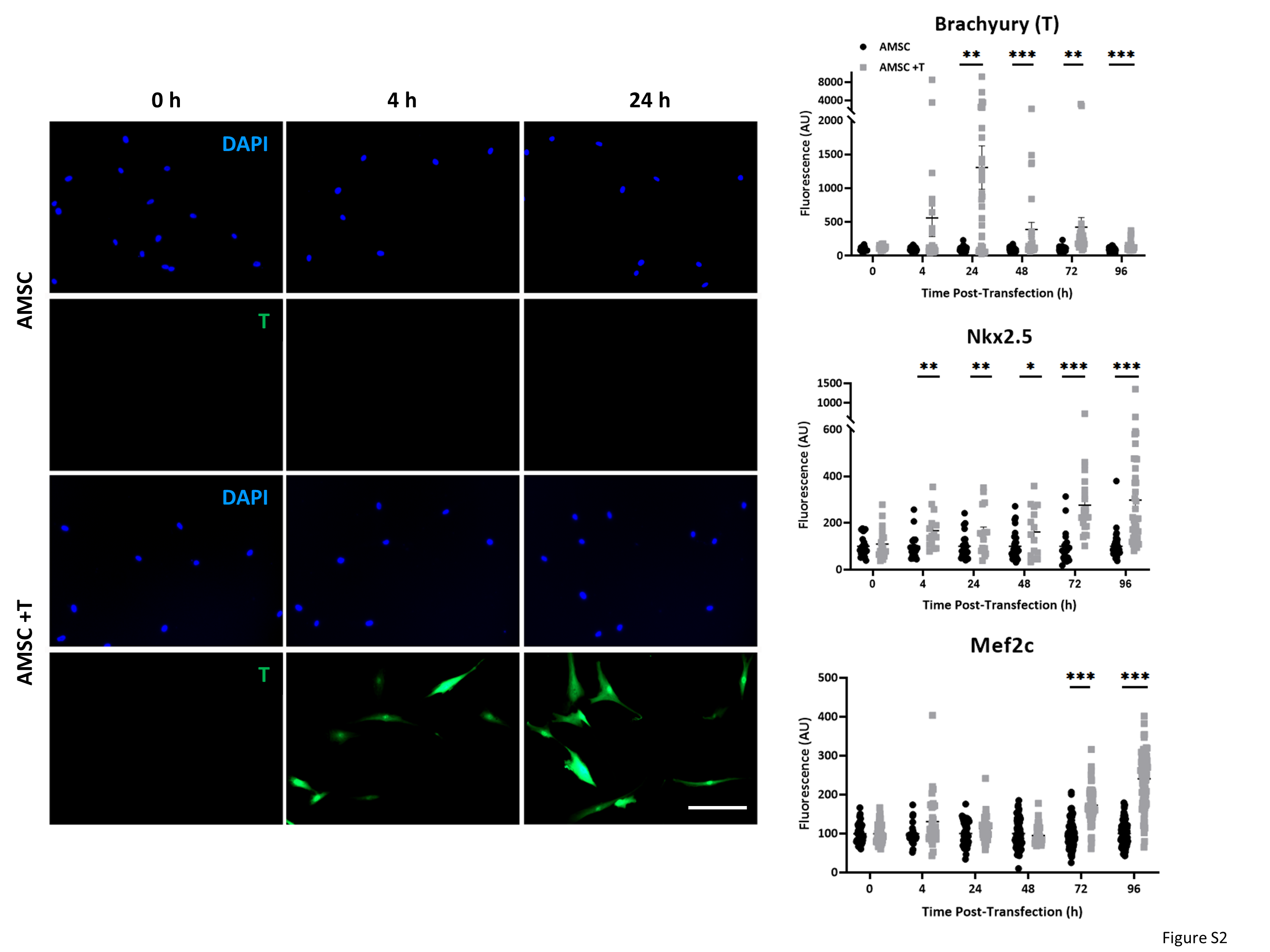

Supplement: Supplementary file 3 — Figure S2 Time course of Brachyury‐induced cardiopoiesis. Representative immunofluorescent images depict increased expression of Brachyury (T) in AMSC transfected using the M3RNA delivery system with a peak level recorded at 24 hours post‐transfection. Induction of cardiopoietic markers, Nkx2.5 and Mef2c, was detected by 72 hours. Fluorescence was normalized to non‐transfected AMSCs for each time point. Numbers of biological replicates (n) were ≥ 17 for T, ≥ 15 for Nkx2.5, and ≥ 25 for Mef2c, per time point. *, P < .05; **, P < .01; ***, P < .001 with Student's t‐test. Scale bar = 100 μm [file SCT3-10-385-s003.TIF]

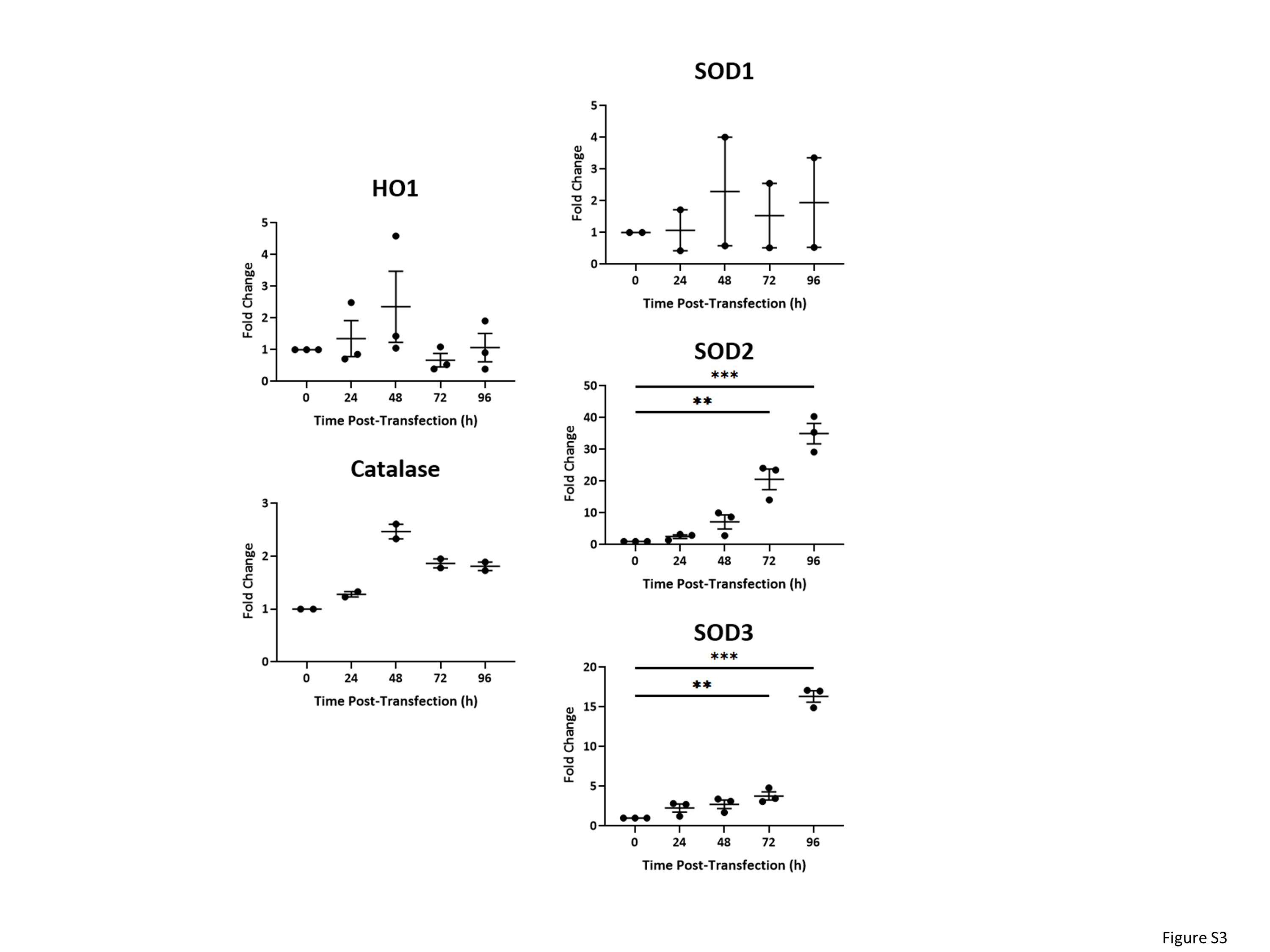

Supplement: Supplementary file 4 — Figure S3 Time course of antioxidant protein expression by Western blot in cell lysates of Brachyury (T) transfected AMSCs. Transfection of AMSCs with T increased levels of superoxide dismutase 2 and 3 (SOD2 (n = 3), SOD3 (n = 3)), but not of heme oxygenase 1 (HO1, n = 3), catalase (n = 2) or SOD1 (n = 2), at the 72‐hours time point. **, P < .01; ***, P < .001 with one‐way ANOVA followed by a post‐hoc Bonferroni test. [file SCT3-10-385-s004.TIF]
