# Supplementary material for: Brachyury engineers cardiac repair competent stem cells
Source: Stem Cells Transl Med. 2020 Oct 24;10(3):385–97. doi: 10.1002/sctm.20-0193 (PMC7900595; doi:10.1002/sctm.20-0193)
Supplement: Supplementary file 5 — Supplemental Table 1 [file SCT3-10-385-s005.docx]

**Supplemental Table 1**

**
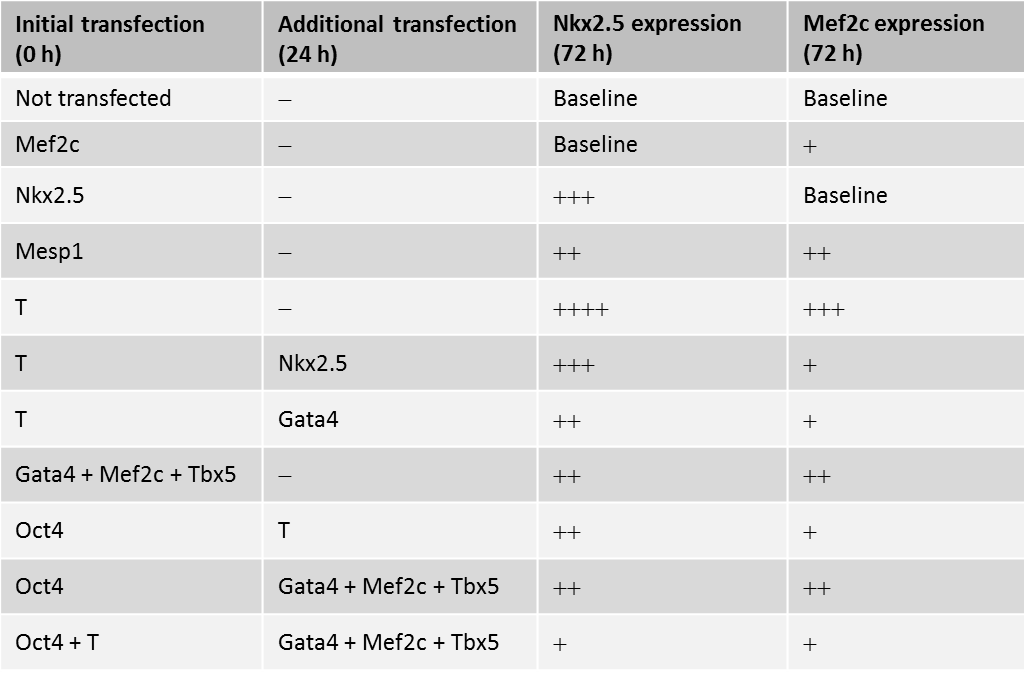
**

Impact of M^3^RNA delivered transcription factors, transfected individually or in combination, on expression of cardiopoietic markers, Nkx2.5 and Mef2c, at 72 h post-transfection in AMSC determined by immunocytochemistry. Single gene Brachyury (T) transfection was the simplest, most effective approach to induce cardiopoiesis. AMSC, human adipose-derived mesenchymal stem cells; Gata4, GATA binding protein 4; Mef2c, myocyte enhancer factor 2C; Mesp1, mesoderm posterior bHLH transcription factor 1; M^3^RNA, microencapsulated-modified-mRNA; Nkx2.5, NK2 homeobox 5; Oct4, octamer-binding transcription factor 4; Tbx5, T-box transcription factor 5.
