# Supplementary material for: Brachyury engineers cardiac repair competent stem cells
Source: Stem Cells Transl Med. 2020 Oct 24;10(3):385–97. doi: 10.1002/sctm.20-0193 (PMC7900595; doi:10.1002/sctm.20-0193)
Supplement: Supplementary file 6 — Supplemental Table 2 [file SCT3-10-385-s006.pdf]

gene_id	logFC	logCPM	P Value	FDR	gene_name	gene_biotype	diff_median_len
ENSG00000206073	5.955721901	-1.430702088	4.99E-06	0.00010549	SERPINB4	protein_coding	912.5
ENSG00000225826	5.527510627	-2.349950395	7.74E-05	0.001117988	LINC00626	lincRNA	955
ENSG00000164458	5.442818032	6.567654347	1.34E-109	9.25E-106	T	protein_coding	1873.5
ENSG00000174576	5.106922045	-1.97155833	0.00221149	0.016988414	NPAS4	protein_coding	2454
ENSG00000069122	4.963062124	-2.291568856	0.003152417	0.022301726	ADGRF5	protein_coding	3138.5
ENSG00000198400	4.959976084	-2.155937257	0.000978742	0.008905439	NTRK1	protein_coding	2462
ENSG00000206965	4.802774994	-2.820777958	0.003177845	0.022437771	RNU6-5P	snRNA	107
ENSG00000279322	4.649767831	-2.82139134	0.004629573	0.030158489	RP4-738P15.6	TEC	288
ENSG00000162951	4.024738423	-2.080393427	4.38E-05	0.000690771	LRRTM1	protein_coding	2126
ENSG00000112559	4.001803792	0.626282507	7.63E-22	1.74E-19	MDFI	protein_coding	703
ENSG00000064300	3.819564443	2.445177477	1.93E-49	3.08E-46	NGFR	protein_coding	1840
ENSG00000117318	3.765414715	7.154623954	1.51E-206	3.13E-202	ID3	protein_coding	892
ENSG00000198523	3.679184206	-0.991917926	2.70E-08	9.87E-07	PLN	protein_coding	2001
ENSG00000262477	3.575018297	-2.631657123	0.002697042	0.019770986	AC021224.1	lincRNA	857
ENSG00000226012	3.56622789	-2.249047824	0.001147279	0.010088745	AP001434.2	lincRNA	287
ENSG00000175899	3.472501947	0.121939944	2.23E-14	2.35E-12	A2M	protein_coding	590
ENSG00000119283	3.453286285	-2.072757948	0.001910016	0.015166614	TRIM67	protein_coding	3936
ENSG00000136244	3.400728848	3.87873956	2.93E-72	1.21E-68	IL6	protein_coding	765
ENSG00000230699	3.314916107	-0.442990936	1.28E-10	7.34E-09	RP11-54O7.1	lincRNA	3043
ENSG00000172201	3.277212657	1.46001106	3.36E-27	1.29E-24	ID4	protein_coding	2344
ENSG00000123243	3.204375049	-1.129035707	6.30E-07	1.69E-05	ITIH5	protein_coding	2651
ENSG00000260633	3.197814265	-2.608091597	0.006359426	0.038451779	RP11-375I20.6	lincRNA	1946
ENSG00000231231	3.172511296	-1.710638464	0.000358782	0.003960563	LINC01423	lincRNA	515.5
ENSG00000066468	3.17157946	0.331121876	1.40E-18	2.30E-16	FGFR2	protein_coding	2621.5
ENSG00000147689	3.09577051	-2.153455095	0.000339895	0.003786002	FAM83A	protein_coding	1131
ENSG00000127588	3.095634573	-0.978372922	0.00037228	0.004076973	GNG13	protein_coding	984
ENSG00000101425	3.036827968	-1.162979515	5.78E-07	1.56E-05	BPI	protein_coding	654.5
ENSG00000128262	3.021872616	-0.025771606	3.35E-09	1.52E-07	POM121L9P	transcribed_unprocessed_pseudogene	1285
ENSG00000184160	3.008320941	0.471894865	1.61E-15	2.01E-13	ADRA2C	protein_coding	1864.5
ENSG00000017427	3.007734793	0.410550846	2.53E-12	1.92E-10	IGF1	protein_coding	761
ENSG00000184845	2.918193004	-2.530770543	0.000765035	0.007297611	DRD1	protein_coding	4054
ENSG00000165694	2.917883621	-1.996928982	0.001420518	0.01201848	FRMD7	protein_coding	2864
ENSG00000115468	2.885471189	-1.101453689	8.19E-05	0.001173686	EFHD1	protein_coding	1490
ENSG00000166920	2.831390547	-2.233196049	0.001542243	0.01277532	C15orf48	protein_coding	547
ENSG00000125968	2.825508385	5.728884926	2.78E-115	2.87E-111	ID1	protein_coding	1113.5
ENSG00000183691	2.795421611	-2.439437053	0.001056949	0.009450932	NOG	protein_coding	1892
ENSG00000187398	2.795002413	-1.313953975	1.25E-07	3.97E-06	LUZP2	protein_coding	2288
ENSG00000170382	2.771550843	-1.493332645	8.61E-07	2.23E-05	LRRN2	protein_coding	3337
ENSG00000211448	2.753104371	-0.171011843	1.37E-12	1.07E-10	DIO2	protein_coding	776
ENSG00000163295	2.733527159	-1.52520917	2.86E-05	0.00047462	ALPI	protein_coding	2216
ENSG00000217027	2.721153971	-2.119454092	0.004265977	0.028261072	TPT1P4	processed_pseudogene	519
ENSG00000261573	2.697101582	0.030223028	2.85E-11	1.80E-09	RP11-553K8.5	antisense	416
ENSG00000115844	2.670313309	-2.41993972	0.000294673	0.00337148	DLX2	protein_coding	2152.5
ENSG00000132854	2.636326891	-1.315557144	4.11E-06	8.87E-05	KANK4	protein_coding	2563
ENSG00000173406	2.635105069	1.635535326	1.49E-27	6.05E-25	DAB1	protein_coding	1150
ENSG00000146674	2.634747716	10.04796803	4.23E-77	2.19E-73	IGFBP3	protein_coding	810
ENSG00000103196	2.630956822	6.855524916	1.15E-35	9.19E-33	CRISPLD2	protein_coding	1123
ENSG00000157570	2.624219453	2.578897388	3.43E-31	2.15E-28	TSPAN18	protein_coding	827
ENSG00000103888	2.585768789	7.86240396	3.94E-37	3.54E-34	CEMIP	protein_coding	3536
ENSG00000225674	2.582763307	-2.485161086	0.006559039	0.039359934	IPO7P2	processed_pseudogene	1750
ENSG00000158050	2.544660343	1.843196216	1.39E-21	3.05E-19	DUSP2	protein_coding	1230.5
ENSG00000138759	2.541492206	1.900757746	3.91E-18	6.23E-16	FRAS1	protein_coding	2257
ENSG00000165507	2.537327031	2.317120468	6.71E-37	5.55E-34	C10orf10	protein_coding	868
ENSG00000133048	2.503984481	3.417343192	2.00E-26	6.91E-24	CHI3L1	protein_coding	921
ENSG00000129824	2.500268531	-2.356443222	0.001875368	0.01495456	RPS4Y1	protein_coding	904.5
ENSG00000164684	2.444440108	-1.077753306	9.63E-06	0.000186752	ZNF704	protein_coding	566.5
ENSG00000257496	2.427853615	-2.680597523	0.006359426	0.038451779	RP11-474P2.4	lincRNA	562
ENSG00000146197	2.407166251	7.526711083	1.39E-64	4.10E-61	SCUBE3	protein_coding	7356
ENSG00000162552	2.373947339	-1.74394983	0.000630996	0.006187166	WNT4	protein_coding	680
ENSG00000162267	2.369286664	-0.407974587	3.61E-08	1.30E-06	ITIH3	protein_coding	824
ENSG00000187634	2.367883918	4.837978055	1.30E-38	1.28E-35	SAMD11	protein_coding	2159
ENSG00000145423	2.34792377	2.301215188	9.80E-19	1.66E-16	SFRP2	protein_coding	2032
ENSG00000215218	2.343026135	1.48393491	1.73E-18	2.79E-16	UBE2QL1	protein_coding	4317
ENSG00000250056	2.340983535	1.348434127	1.75E-16	2.41E-14	LINC01018	lincRNA	1405
ENSG00000124216	2.331284709	2.915341158	2.44E-23	6.48E-21	SNAI1	protein_coding	1686
ENSG00000124772	2.324649151	-2.330613531	0.00708039	0.041514696	CPNE5	protein_coding	1567
ENSG00000137745	2.321049193	-1.46324991	0.000240165	0.002837183	MMP13	protein_coding	1470
ENSG00000241644	2.302005051	0.09408158	3.57E-11	2.22E-09	INMT	protein_coding	930
ENSG00000171385	2.287851607	3.418082578	7.72E-23	2.00E-20	KCND3	protein_coding	2716
ENSG00000011201	2.26648865	-1.285604422	4.79E-05	0.000746651	ANOS1	protein_coding	797
ENSG00000003989	2.246415694	-2.068005113	0.002560307	0.019038206	SLC7A2	protein_coding	7560
ENSG00000185345	2.206208859	-0.115136709	5.68E-08	1.96E-06	PARK2	protein_coding	1157
ENSG00000184557	2.197315359	3.899808015	1.84E-54	3.17E-51	SOCS3	protein_coding	1547.5
ENSG00000117643	2.174661628	1.824165471	4.01E-19	7.03E-17	MAN1C1	protein_coding	2544
ENSG00000260941	2.170592459	-1.591103217	0.000111161	0.001514849	LINC00622	sense_overlapping	1570
ENSG00000134198	2.168518827	3.948479418	1.74E-38	1.64E-35	TSPAN2	protein_coding	732
ENSG00000164694	2.159593694	3.655239542	2.13E-29	1.10E-26	FNDC1	protein_coding	6034
ENSG00000160161	2.150063453	3.724768175	4.45E-24	1.26E-21	CILP2	protein_coding	4199
ENSG00000103044	2.130759409	1.292620809	2.37E-14	2.46E-12	HAS3	protein_coding	1163

ENSG00000167157	2.130239097	3.593300213	1.84E-41	2.00E-38	PRRX2	protein_coding	1311
ENSG00000038295	2.125583853	-1.617987521	0.000104618	0.00144147	TLL1	protein_coding	3308
ENSG00000125730	2.116614007	4.940443752	1.57E-56	3.22E-53	C3	protein_coding	580.5
ENSG00000072274	2.114757155	7.203768667	1.22E-08	4.88E-07	TFRC	protein_coding	560
ENSG00000128045	2.09647996	-1.05835416	1.14E-06	2.88E-05	RASL11B	protein_coding	1733
ENSG00000149596	2.087923389	0.57352884	7.07E-12	5.07E-10	JPH2	protein_coding	3355
ENSG00000115594	2.087296789	7.191757224	5.15E-18	8.08E-16	IL1R1	protein_coding	1678
ENSG00000050628	2.080753392	0.106986244	8.59E-08	2.85E-06	PTGER3	protein_coding	1904.5
ENSG00000099994	2.078563743	3.123241041	3.08E-27	1.20E-24	SUSD2	protein_coding	4171
ENSG00000111913	2.04904549	-1.134519733	0.000307081	0.003494145	FAM65B	protein_coding	3614
ENSG00000254204	2.033578515	-2.417276648	0.008628225	0.047904	RP11-400K9.3	lincRNA	469
ENSG00000099998	2.028912056	3.117631901	1.40E-26	4.93E-24	GGT5	protein_coding	2371
ENSG00000168874	1.98144227	4.365832107	3.99E-20	7.72E-18	ATOH8	protein_coding	1893
ENSG00000174325	1.979307825	-0.713114315	6.65E-05	0.000980717	DIRC1	protein_coding	1037
ENSG00000233901	1.979236079	3.320112924	6.07E-30	3.22E-27	LINC01503	lincRNA	664
ENSG00000206052	1.978101956	0.02710769	8.18E-09	3.43E-07	DOK6	protein_coding	506
ENSG00000126010	1.977105045	-1.428079793	0.002310356	0.017553736	GRPR	protein_coding	1929
ENSG00000146477	1.963064769	-0.889561038	4.00E-05	0.000637701	SLC22A3	protein_coding	3348
ENSG00000130600	1.959822196	7.215560677	1.27E-18	2.11E-16	H19	processed_transcript	1546.5
ENSG00000137834	1.954361341	3.199416997	2.16E-21	4.56E-19	SMAD6	protein_coding	1401
ENSG00000116039	1.945195991	-0.664653328	4.30E-05	0.000680474	ATP6V1B1	protein_coding	718.5
ENSG00000072952	1.941398822	3.575970348	1.46E-25	4.64E-23	MRVI1	protein_coding	2468
ENSG00000272970	1.936970445	-1.06695824	0.000286335	0.003288799	RP11-329B9.4	lincRNA	701
ENSG00000187955	1.934903994	7.520335974	7.27E-11	4.33E-09	COL14A1	protein_coding	3244
ENSG00000106538	1.934052833	4.262779053	8.23E-28	3.55E-25	RARRES2	protein_coding	740
ENSG00000221852	1.926731215	-1.350852325	0.008411404	0.047104318	KRTAP1-5	protein_coding	1177
ENSG00000206932	1.919276182	-2.004104491	0.003295406	0.023040025	RNU6-4P	snRNA	107
ENSG00000265018	1.919158312	-0.672870838	4.50E-05	0.000706507	AGAP12P	transcribed_unprocessed_pseudogene	2250
ENSG00000161249	1.913010741	-0.045941618	2.42E-08	8.98E-07	DMKN	protein_coding	678.5
ENSG00000155792	1.90221575	5.069914963	4.57E-20	8.77E-18	DEPTOR	protein_coding	1397
ENSG00000111341	1.888523554	3.730799641	5.02E-15	5.94E-13	MGP	protein_coding	1005
ENSG00000149131	1.887909035	5.876088016	1.25E-21	2.78E-19	SERPING1	protein_coding	956
ENSG00000182329	1.880639883	-1.385470994	6.76E-05	0.000994589	KIAA2012	protein_coding	3088.5
ENSG00000148677	1.871823673	0.759438473	4.32E-07	1.21E-05	ANKRD1	protein_coding	1979
ENSG00000144802	1.864989226	4.027182774	3.38E-07	9.74E-06	NFKBIZ	protein_coding	877
ENSG00000115738	1.86301273	5.706203915	1.71E-56	3.22E-53	ID2	protein_coding	992.5
ENSG00000163017	1.861207176	-0.495870977	4.87E-06	0.000103328	ACTG2	protein_coding	927
ENSG00000010932	1.85844363	2.058167479	1.43E-11	9.42E-10	FMO1	protein_coding	2003
ENSG00000155511	1.852174836	0.373723693	1.72E-11	1.12E-09	GRIA1	protein_coding	2069
ENSG00000120693	1.85080365	3.63679782	1.08E-44	1.39E-41	SMAD9	protein_coding	5418
ENSG00000164056	1.849523152	1.247974604	1.46E-09	7.16E-08	SPRY1	protein_coding	1680
ENSG00000076716	1.848455478	2.32369804	6.26E-18	9.68E-16	GPC4	protein_coding	4960
ENSG00000125657	1.842449768	1.679190624	1.36E-18	2.25E-16	TNFSF9	protein_coding	1665
ENSG00000109625	1.794021409	-0.532022327	0.00043679	0.004624485	CPZ	protein_coding	906
ENSG00000167680	1.789781083	1.503188932	4.89E-12	3.58E-10	SEMA6B	protein_coding	2034
ENSG00000132329	1.787222137	1.177494482	1.54E-10	8.77E-09	RAMP1	protein_coding	795.5
ENSG00000177283	1.784727048	4.726770518	1.99E-15	2.45E-13	FZD8	protein_coding	2055.5
ENSG00000122176	1.775604672	3.781316037	5.48E-39	5.67E-36	FMOD	protein_coding	717
ENSG00000177679	1.774729552	0.470556627	4.73E-07	1.31E-05	SRRM3	protein_coding	2150
ENSG00000184185	1.770467304	0.743671264	5.68E-09	2.47E-07	KCNJ12	protein_coding	3811.5
ENSG00000197361	1.766407246	1.24674848	2.66E-08	9.75E-07	FBXL22	protein_coding	1526
ENSG00000140285	1.758026619	6.836128598	1.69E-63	4.38E-60	FGF7	protein_coding	1388.5
ENSG00000204174	1.754223347	-0.849949235	0.002339583	0.017727927	NPY4R	protein_coding	1878
ENSG00000118849	1.747375503	3.543617739	1.96E-33	1.45E-30	RARRES1	protein_coding	532
ENSG00000107796	1.746807607	8.631987801	9.38E-31	5.40E-28	ACTA2	protein_coding	820
ENSG00000104213	1.746151416	1.10497186	5.52E-09	2.42E-07	PDGFRL	protein_coding	1901
ENSG00000130751	1.732197192	1.15471412	1.36E-09	6.69E-08	NPAS1	protein_coding	1052.5
ENSG00000176928	1.721588674	-0.884616098	0.000967961	0.008815083	GCNT4	protein_coding	5554
ENSG00000267565	1.720451996	-1.222203039	0.000872032	0.008105435	CTC-559E9.8	antisense	403
ENSG00000027644	1.71877155	-1.298986951	0.008949681	0.049305388	INSRR	protein_coding	5101
ENSG00000177839	1.715108116	-1.225877291	0.002555659	0.019017297	PCDHB9	protein_coding	616
ENSG00000198948	1.712100614	4.273834962	3.65E-35	2.80E-32	MFAP3L	protein_coding	564
ENSG00000166979	1.71117009	-0.676501074	0.005534145	0.034592997	EVA1C	protein_coding	1558
ENSG00000224081	1.709865364	0.360800686	1.23E-07	3.91E-06	LINC01057	transcribed_processed_pseudogene	893
ENSG00000234975	1.707739885	-1.002048508	0.002672698	0.01966663	FTH1P2	processed_pseudogene	412
ENSG00000109610	1.704594665	3.317848076	1.36E-22	3.40E-20	SOD3	protein_coding	567
ENSG00000205403	1.700066176	3.642620458	2.03E-21	4.34E-19	CFI	protein_coding	1914
ENSG00000108691	1.696204577	4.203729722	1.74E-25	5.39E-23	CCL2	protein_coding	866.5
ENSG00000258667	1.695779223	-1.336816876	0.000558262	0.005625813	HIF1A-AS2	lincRNA	533
ENSG00000196562	1.681539321	6.632721996	4.40E-18	6.96E-16	SULF2	protein_coding	1066.5
ENSG00000116990	1.674368163	0.07997233	3.78E-07	1.07E-05	MYCL	protein_coding	1816
ENSG00000115457	1.669636201	1.092542795	8.83E-09	3.66E-07	IGFBP2	protein_coding	846
ENSG00000082196	1.665150458	2.013670958	1.00E-11	6.85E-10	C1QTNF3	protein_coding	1474.5
ENSG00000221869	1.662550787	5.888372528	9.20E-17	1.29E-14	CEBPD	protein_coding	2178
ENSG00000228221	1.660453101	-1.315387831	0.003384436	0.023541393	LINC00578	lincRNA	530.5
ENSG00000254510	1.651475161	-0.889902743	0.00061647	0.006079272	RP11-867G23.10	processed_transcript	637
ENSG00000115598	1.648700184	0.304838707	6.05E-07	1.63E-05	IL1RL2	protein_coding	1485.5
ENSG00000180914	1.636753532	5.112832957	2.38E-16	3.22E-14	OXTR	protein_coding	671.5
ENSG00000233098	1.633909313	-0.492596638	9.28E-05	0.001298283	CCDC144NL-AS1	antisense	821
ENSG00000116690	1.628689435	5.619013608	1.15E-13	1.05E-11	PRG4	protein_coding	4840

ENSG00000144810	1.621302496	8.28135144	5.61E-46	7.75E-43	COL8A1	protein_coding	894.5
ENSG00000166402	1.618703618	3.10568719	3.19E-29	1.57E-26	TUB	protein_coding	2019
ENSG00000130303	1.615429183	0.25236156	7.14E-06	0.000144198	BST2	protein_coding	459
ENSG00000049540	1.596370106	4.799776283	9.79E-28	4.14E-25	ELN	protein_coding	733
ENSG00000157554	1.596162878	0.391569289	3.92E-07	1.11E-05	ERG	protein_coding	3259.5
ENSG00000224389	1.595023061	-0.691297071	0.000179948	0.002240857	C4B	protein_coding	605.5
ENSG00000104881	1.585184885	4.883425486	5.13E-26	1.73E-23	PPP1R13L	protein_coding	648
ENSG00000167244	1.584352668	1.018730987	7.84E-07	2.06E-05	IGF2	protein_coding	1583
ENSG00000183196	1.575691945	3.819886483	1.05E-27	4.35E-25	CHST6	protein_coding	5025
ENSG00000182575	1.569105704	-1.572162588	0.002725879	0.019940032	NXPH3	protein_coding	2179
ENSG00000104043	1.566561736	-0.619346743	0.000129821	0.001716773	ATP8B4	protein_coding	887
ENSG00000107317	1.558403825	-0.48750841	0.000203315	0.002462251	PTGDS	protein_coding	661
ENSG00000149970	1.558033283	1.112180293	3.70E-07	1.05E-05	CNKSR2	protein_coding	4355
ENSG00000115380	1.557348099	10.08293743	2.39E-57	5.50E-54	EFEMP1	protein_coding	580
ENSG00000159403	1.541401261	7.976939058	2.78E-22	6.62E-20	C1R	protein_coding	614.5
ENSG00000135917	1.541369814	-0.637081027	0.000471001	0.004918324	SLC19A3	protein_coding	707.5
ENSG00000131471	1.53969952	-0.52060375	0.004392925	0.028891192	AOC3	protein_coding	2392
ENSG00000091879	1.539644447	2.083141239	1.79E-09	8.66E-08	ANGPT2	protein_coding	2799.5
ENSG00000170381	1.538428793	-1.5548506	0.008341423	0.046839079	SEMA3E	protein_coding	1520
ENSG00000106823	1.533058642	4.319073466	4.62E-07	1.28E-05	ECM2	protein_coding	2127
ENSG00000263176	1.530437272	-1.590439982	0.006017371	0.036857066	RP11-893F2.15	lincRNA	578
ENSG00000132003	1.528733376	2.967381455	8.64E-17	1.22E-14	ZSWIM4	protein_coding	2921
ENSG00000254343	1.513299535	1.539068235	5.16E-07	1.42E-05	RP11-760H22.2	lincRNA	506
ENSG00000132821	1.511829058	0.957740105	1.35E-06	3.34E-05	VSTM2L	protein_coding	831
ENSG00000134508	1.511084496	3.677810624	1.96E-20	3.94E-18	CABLES1	protein_coding	594
ENSG00000105664	1.503929649	6.901654497	6.40E-11	3.88E-09	COMP	protein_coding	2376.5
ENSG00000237412	1.502432476	-0.332458581	7.30E-05	0.001063367	PRSS56	protein_coding	2157
ENSG00000101670	1.499908448	3.380547193	2.11E-17	3.10E-15	LIPG	protein_coding	1760
ENSG00000282849	1.498328253	1.846031099	2.97E-12	2.23E-10	RP11-121P12.1	lincRNA	638
ENSG00000102683	1.492358134	2.456316685	5.14E-13	4.34E-11	SGCG	protein_coding	1624
ENSG00000169116	1.492031161	-1.630491707	0.0032739	0.022947272	PARM1	protein_coding	4659.5
ENSG00000144893	1.490713946	-0.568218502	0.000419489	0.004487188	MED12L	protein_coding	749
ENSG00000111907	1.487198133	2.762894286	1.89E-12	1.45E-10	TPD52L1	protein_coding	999
ENSG00000071575	1.485869018	5.244292326	2.12E-15	2.60E-13	TRIB2	protein_coding	1804
ENSG00000185760	1.484627706	0.649296541	0.000259952	0.003036292	KCNQ5	protein_coding	6236
ENSG00000163431	1.483311041	4.832192179	1.96E-10	1.09E-08	LMOD1	protein_coding	3514
ENSG00000133321	1.482682523	0.906200764	3.99E-07	1.13E-05	RARRES3	protein_coding	696
ENSG00000127329	1.478226824	3.599385353	1.19E-06	2.97E-05	PTPRB	protein_coding	5291
ENSG00000168685	1.477424435	2.776465716	3.26E-09	1.49E-07	IL7R	protein_coding	619
ENSG00000175745	1.472426211	3.77774475	7.37E-27	2.68E-24	NR2F1	protein_coding	796
ENSG00000138771	1.471371419	4.01297694	3.99E-19	7.03E-17	SHROOM3	protein_coding	619.5
ENSG00000174348	1.469541011	6.162613356	2.16E-23	5.80E-21	PODN	protein_coding	2744
ENSG00000022267	1.467023463	5.606739531	2.81E-30	1.53E-27	FHL1	protein_coding	796
ENSG00000136153	1.463081677	7.375773964	3.90E-25	1.19E-22	LMO7	protein_coding	914
ENSG00000110900	1.448609669	0.210994795	3.40E-05	0.000554018	TSPAN11	protein_coding	1353.5
ENSG00000111452	1.447245099	2.482490044	1.50E-10	8.54E-09	ADGRD1	protein_coding	726.5
ENSG00000088882	1.446252714	3.554764124	1.96E-11	1.28E-09	CPXM1	protein_coding	2391
ENSG00000187678	1.445506563	3.092872499	6.64E-11	4.01E-09	SPRY4	protein_coding	575
ENSG00000179841	1.442003992	1.227370137	4.75E-06	0.000101101	AKAP5	protein_coding	4544
ENSG00000150907	1.441853267	1.173353543	1.05E-08	4.28E-07	FOXO1	protein_coding	1773
ENSG00000166278	1.436484571	1.021837619	1.61E-07	5.02E-06	C2	protein_coding	1609
ENSG00000118513	1.434253559	-0.31736746	0.00847792	0.047323244	MYB	protein_coding	2478.5
ENSG00000132182	1.43269086	-1.11678884	0.003672154	0.025064809	NUP210	protein_coding	2223
ENSG00000182326	1.430056875	8.661997581	3.95E-24	1.14E-21	C1S	protein_coding	667
ENSG00000182752	1.428186528	5.656599164	2.14E-22	5.29E-20	PAPPA	protein_coding	790
ENSG00000124302	1.416759847	0.510467651	3.76E-06	8.19E-05	CHST8	protein_coding	2133
ENSG00000100336	1.41560207	0.785756893	8.96E-09	3.69E-07	APOL4	protein_coding	844.5
ENSG00000259450	1.410773261	0.140061729	0.00032719	0.003668532	RP11-265N7.1	lincRNA	1961
ENSG00000153012	1.401891896	2.267228266	4.87E-10	2.54E-08	LGI2	protein_coding	3597.5
ENSG00000127472	1.398680621	-1.129339963	0.005424371	0.034112753	PLA2G5	protein_coding	1028
ENSG00000184347	1.397986202	8.074487476	1.62E-17	2.42E-15	SLIT3	protein_coding	3159
ENSG00000185885	1.396321819	4.531010865	3.92E-24	1.14E-21	IFITM1	protein_coding	787
ENSG00000262655	1.395510858	4.68766779	4.64E-28	2.04E-25	SPON1	protein_coding	2921
ENSG00000091129	1.38891039	2.404032235	3.67E-12	2.74E-10	NRCAM	protein_coding	1594
ENSG00000101115	1.386532016	-0.178463224	0.000698443	0.006748167	SALL4	protein_coding	1151
ENSG00000151090	1.385592682	2.707279781	2.16E-13	1.90E-11	THRB	protein_coding	728.5
ENSG00000101938	1.382347417	4.202336076	2.15E-15	2.62E-13	CHRDL1	protein_coding	1934
ENSG00000178882	1.382313697	-0.995741298	0.007800191	0.044672055	FAM101A	protein_coding	2148
ENSG00000198108	1.380304334	3.315751168	5.22E-14	5.10E-12	CHSY3	protein_coding	2243
ENSG00000113212	1.379269665	0.227109427	4.99E-06	0.00010549	PCDHB7	protein_coding	3765
ENSG00000185565	1.376456895	3.646420633	8.02E-15	9.03E-13	LSAMP	protein_coding	772
ENSG00000154864	1.375060982	3.077535095	5.65E-09	2.46E-07	PIEZO2	protein_coding	1846
ENSG00000175426	1.367789357	-0.586343867	5.98E-05	0.000896785	PCSK1	protein_coding	1857.5
ENSG00000165124	1.361757049	3.721506749	7.76E-17	1.11E-14	SVEP1	protein_coding	5124
ENSG00000143333	1.3563282	-0.325111091	0.004730502	0.030671245	RGS16	protein_coding	2427
ENSG00000184371	1.355089747	5.979666414	4.95E-37	4.27E-34	CSF1	protein_coding	1009
ENSG00000100342	1.354020813	5.591762324	1.54E-15	1.94E-13	APOL1	protein_coding	612
ENSG00000156466	1.349920377	5.337234059	2.43E-06	5.65E-05	GDF6	protein_coding	1179
ENSG00000164530	1.347100591	5.952320999	7.46E-22	1.74E-19	PI16	protein_coding	2083
ENSG00000047936	1.346982678	0.500505903	1.75E-05	0.000311542	ROS1	protein_coding	7417

ENSG00000103522	1.34546689	0.994545495	2.08E-05	0.00035943	IL21R	protein_coding	2953
ENSG00000162551	1.341755952	6.569286129	1.91E-42	2.19E-39	ALPL	protein_coding	2131
ENSG00000223764	1.339973202	2.021589743	0.001533768	0.012730581	RP11-54O7.3	lincRNA	1389
ENSG00000147234	1.337001403	-0.005927793	2.51E-06	5.81E-05	FRMPD3	protein_coding	7155
ENSG00000109063	1.33293937	0.839801744	5.50E-05	0.000835608	MYH3	protein_coding	583
ENSG00000099864	1.332790187	1.836267278	8.14E-07	2.12E-05	PALM	protein_coding	2137
ENSG00000267041	1.331799972	0.64682875	0.000138385	0.001814951	ZNF850	protein_coding	7625
ENSG00000149633	1.327164905	2.949756411	4.30E-11	2.66E-09	KIAA1755	protein_coding	2349
ENSG00000137809	1.326354381	8.411988027	4.93E-24	1.38E-21	ITGA11	protein_coding	670
ENSG00000162804	1.320767828	4.726025107	2.62E-14	2.70E-12	SNED1	protein_coding	1446.5
ENSG00000125414	1.314660248	0.394927353	0.00023075	0.002740021	MYH2	protein_coding	2453.5
ENSG00000064042	1.310439345	-0.336216074	0.000464331	0.004858932	LIMCH1	protein_coding	3456.5
ENSG00000118523	1.299920041	9.630004256	5.88E-19	1.02E-16	CTGF	protein_coding	2339
ENSG00000117525	1.297647941	5.444039883	8.72E-31	5.16E-28	F3	protein_coding	1536
ENSG00000171223	1.289410551	5.974929825	6.72E-26	2.21E-23	JUNB	protein_coding	1820
ENSG00000158079	1.284736405	3.81767423	6.34E-23	1.66E-20	PTPDC1	protein_coding	4417.5
ENSG00000119630	1.276313019	0.927890221	1.20E-05	0.000223796	PGF	protein_coding	1409
ENSG00000119280	1.275731126	7.128168606	5.90E-33	4.07E-30	C1orf198	protein_coding	1041
ENSG00000115419	1.274480028	8.615184104	5.29E-16	6.89E-14	GLS	protein_coding	693
ENSG00000184635	1.273452164	3.592116075	6.81E-17	9.79E-15	ZNF93	protein_coding	590
ENSG00000101680	1.270226042	6.548511787	2.23E-27	8.89E-25	LAMA1	protein_coding	2565.5
ENSG00000117152	1.269359692	5.041019573	4.98E-29	2.40E-26	RGS4	protein_coding	873.5
ENSG00000107957	1.269128166	9.053269131	2.31E-29	1.17E-26	SH3PXD2A	protein_coding	8374.5
ENSG00000183598	1.269070304	-0.708789479	0.001062108	0.009488864	HIST2H3D	protein_coding	411
ENSG00000248187	1.26553115	2.225267139	3.91E-06	8.50E-05	RP11-184M15.1	lincRNA	1623
ENSG00000105088	1.261029553	0.071147427	0.000119872	0.00160988	OLFM2	protein_coding	1226
ENSG00000184304	1.255965328	4.994259743	5.18E-26	1.73E-23	PRKD1	protein_coding	585
ENSG00000277758	1.253935891	0.870496349	5.38E-05	0.000822211	ABC7-42404400C24.1	protein_coding	1370
ENSG00000156140	1.253809246	1.504752377	3.23E-06	7.21E-05	ADAMTS3	protein_coding	2740.5
ENSG00000126803	1.252719354	1.789408064	1.39E-06	3.41E-05	HSPA2	protein_coding	2767
ENSG00000162614	1.250766173	6.29725958	2.75E-20	5.43E-18	NEXN	protein_coding	1714
ENSG00000227992	1.250447755	0.647571844	0.00044555	0.004702797	AC108463.2	processed_pseudogene	347
ENSG00000169418	1.24977598	-0.711409747	0.005413298	0.034094683	NPR1	protein_coding	1706
ENSG00000198028	1.245143216	-0.182106355	0.00096281	0.008778199	ZNF560	protein_coding	1585
ENSG00000151414	1.240824388	9.398866572	1.27E-15	1.61E-13	NEK7	protein_coding	586.5
ENSG00000223953	1.238715629	0.633402797	2.14E-05	0.000369118	C1QTNF5	protein_coding	814.5
ENSG00000109762	1.237477747	5.708599928	1.07E-26	3.82E-24	SNX25	protein_coding	1937
ENSG00000137124	1.235405194	6.800144057	1.36E-05	0.000250319	ALDH1B1	protein_coding	1825
ENSG00000182118	1.233498039	1.950860756	0.000578664	0.005791955	FAM89A	protein_coding	1239
ENSG00000197442	1.231381028	0.671999192	0.005423276	0.034112753	MAP3K5	protein_coding	2781
ENSG00000279118	1.231314397	3.015367821	4.59E-12	3.37E-10	RP11-517I3.2	TEC	2326
ENSG00000124225	1.230897517	3.421650317	2.97E-06	6.71E-05	PMEPA1	protein_coding	2689
ENSG00000060718	1.223952571	8.694449967	9.20E-20	1.73E-17	COL11A1	protein_coding	1709
ENSG00000151418	1.222284871	-0.909095852	0.004817602	0.031109192	ATP6V1G3	protein_coding	620.5
ENSG00000163661	1.220015337	8.873479369	9.00E-23	2.30E-20	PTX3	protein_coding	1940
ENSG00000147408	1.216741823	1.678024809	1.50E-07	4.70E-06	CSGALNACT1	protein_coding	706
ENSG00000226608	1.212033505	-0.500890576	0.001060326	0.00947704	FTLP3	processed_pseudogene	528
ENSG00000128422	1.208985843	-0.170510744	0.000250473	0.002933848	KRT17	protein_coding	697
ENSG00000028137	1.205618889	-0.475719146	0.003726517	0.025352314	TNFRSF1B	protein_coding	557
ENSG00000250899	1.205266431	2.033440635	0.004900073	0.031470457	RP11-253E3.3	lincRNA	3514
ENSG00000145681	1.204456003	4.24932646	3.93E-19	7.03E-17	HAPLN1	protein_coding	823
ENSG00000127399	1.203263179	-0.135075976	0.00159329	0.013098631	LRRC61	protein_coding	1456
ENSG00000055813	1.200084785	3.112085321	2.18E-13	1.91E-11	CCDC85A	protein_coding	3982
ENSG00000021645	1.199224581	2.347787695	3.57E-08	1.28E-06	NRXN3	protein_coding	2750.5
ENSG00000239617	1.196773483	-1.360628885	0.006946476	0.041052758	RP11-302B13.1	processed_pseudogene	396
ENSG00000113578	1.195003947	4.342285795	2.10E-07	6.39E-06	FGF1	protein_coding	975
ENSG00000169330	1.193648019	2.345917601	3.53E-13	3.04E-11	KIAA1024	protein_coding	6180.5
ENSG00000189001	1.186699045	2.899051123	4.83E-11	2.96E-09	SBSN	protein_coding	957
ENSG00000071282	1.18550995	6.731347024	2.78E-09	1.30E-07	LMCD1	protein_coding	1113
ENSG00000204389	1.182244361	4.027117595	2.70E-12	2.04E-10	HSPA1A	protein_coding	2196
ENSG00000099860	1.180729644	5.405514874	6.36E-15	7.28E-13	GADD45B	protein_coding	750
ENSG00000126804	1.179868327	5.66821387	2.95E-31	1.91E-28	ZBTB1	protein_coding	605.5
ENSG00000180139	1.179314942	1.312831505	9.63E-08	3.12E-06	ACTA2-AS1	antisense	810.5
ENSG00000159216	1.17785654	7.053257422	3.66E-20	7.15E-18	RUNX1	protein_coding	1582
ENSG00000135919	1.177537136	8.525630307	1.19E-30	6.64E-28	SERPINE2	protein_coding	1352
ENSG00000166741	1.175951739	8.235942632	1.19E-05	0.000221664	NNMT	protein_coding	590
ENSG00000184500	1.174615173	4.893975688	1.59E-21	3.43E-19	PROS1	protein_coding	806
ENSG00000070495	1.174143928	4.583602092	3.16E-09	1.45E-07	JMJD6	protein_coding	1748
ENSG00000227496	1.171425347	-1.135029236	0.008086392	0.045749972	RP11-145A3.1	antisense	1530
ENSG00000154175	1.169774466	8.69020915	4.07E-14	4.09E-12	ABI3BP	protein_coding	566
ENSG00000133110	1.169193106	12.73064478	1.01E-21	2.28E-19	POSTN	protein_coding	3092
ENSG00000064932	1.16775036	6.037295152	6.50E-27	2.40E-24	SBNO2	protein_coding	1061.5
ENSG00000272674	1.165605712	-0.140128749	0.000523267	0.005335468	PCDHB16	protein_coding	2947.5
ENSG00000142089	1.164353048	8.251067111	3.31E-28	1.49E-25	IFITM3	protein_coding	628
ENSG00000163520	1.162180836	8.922339597	2.56E-22	6.25E-20	FBLN2	protein_coding	584
ENSG00000148344	1.157685783	3.891368796	9.71E-17	1.35E-14	PTGES	protein_coding	1821
ENSG00000158258	1.15612227	2.219448431	3.40E-05	0.000553784	CLSTN2	protein_coding	8303.5
ENSG00000244242	1.152672627	-0.599387898	0.003243374	0.02277621	IFITM10	protein_coding	2634.5
ENSG00000111057	1.151644065	5.692460859	4.01E-05	0.000639339	KRT18	protein_coding	1420
ENSG00000254810	1.151332207	-0.272895903	0.001251933	0.010879679	RP11-672A2.4	lincRNA	651

ENSG00000150687	1.150074271	9.476630118	5.03E-17	7.28E-15	PRSS23	protein_coding	907
ENSG00000184258	1.149823069	1.135797587	1.75E-07	5.39E-06	CDR1	protein_coding	2467
ENSG00000175697	1.140999889	-0.238157718	0.000192226	0.002352722	GPR156	protein_coding	4154
ENSG00000151276	1.135707165	5.146053453	8.25E-14	7.66E-12	MAGI1	protein_coding	2646
ENSG00000133083	1.135470459	3.104329369	1.26E-11	8.44E-10	DCLK1	protein_coding	4929.5
ENSG00000170962	1.128979762	5.642890738	9.49E-09	3.89E-07	PDGFD	protein_coding	3801
ENSG00000157551	1.128228548	2.012254531	0.00339698	0.023614657	KCNJ15	protein_coding	715
ENSG00000185686	1.127317743	-0.719914405	0.006212364	0.037783215	PRAME	protein_coding	815.5
ENSG00000138669	1.122697622	-0.33393802	0.004840269	0.031197366	PRKG2	protein_coding	2623
ENSG00000237807	1.119759387	1.12474012	3.93E-06	8.53E-05	RP11-400K9.4	lincRNA	2387
ENSG00000117069	1.116018232	1.372209159	1.02E-05	0.000195804	ST6GALNAC5	protein_coding	787
ENSG00000153885	1.114459081	6.03372999	6.75E-08	2.29E-06	KCTD15	protein_coding	649
ENSG00000120324	1.114187417	-0.499078282	0.005448063	0.034217335	PCDHB10	protein_coding	3290
ENSG00000101463	1.111218098	2.387724739	3.68E-06	8.05E-05	SYNDIG1	protein_coding	1469.5
ENSG00000137959	1.109145352	3.094759881	1.20E-06	3.00E-05	IFI44L	protein_coding	869.5
ENSG00000170345	1.108716086	2.755228929	1.36E-06	3.36E-05	FOS	protein_coding	815
ENSG00000107821	1.1058806	3.529785052	1.48E-14	1.60E-12	KAZALD1	protein_coding	925
ENSG00000169174	1.10470094	4.12293994	4.40E-13	3.74E-11	PCSK9	protein_coding	3764
ENSG00000183578	1.10348856	2.548439344	0.003241963	0.022774021	TNFAIP8L3	protein_coding	2132
ENSG00000182389	1.100822366	2.355265698	1.13E-09	5.63E-08	CACNB4	protein_coding	3182.5
ENSG00000153162	1.096028322	1.058706626	1.08E-05	0.000205242	BMP6	protein_coding	2780
ENSG00000177694	1.09204164	3.489167544	3.11E-14	3.16E-12	NAALADL2	protein_coding	789.5
ENSG00000251085	1.084943698	-0.549185355	0.008420866	0.047131813	RP11-893F2.6	lincRNA	455
ENSG00000204941	1.080949772	2.97089614	1.07E-11	7.31E-10	PSG5	protein_coding	1064
ENSG00000233251	1.080932588	0.919565528	3.76E-06	8.19E-05	AC007743.1	antisense	1657
ENSG00000260604	1.079022532	2.845126599	8.93E-09	3.68E-07	RP1-140K8.5	lincRNA	7060
ENSG00000175084	1.078858613	1.095612654	9.71E-06	0.00018781	DES	protein_coding	733
ENSG00000121898	1.073996985	-0.205388435	0.002307903	0.017553736	CPXM2	protein_coding	2580
ENSG00000183801	1.073065123	3.680320282	6.19E-07	1.66E-05	OLFML1	protein_coding	1438
ENSG00000109339	1.070897413	0.021967306	0.001319866	0.011351959	MAPK10	protein_coding	595
ENSG00000250548	1.068592746	0.01664253	0.000540929	0.005475122	RP11-47I22.2	lincRNA	2786.5
ENSG00000142871	1.06418836	9.420096291	0.000626446	0.006157126	CYR61	protein_coding	1420.5
ENSG00000139304	1.063834072	1.364456804	7.76E-05	0.001120002	PTPRQ	protein_coding	1087.5
ENSG00000205710	1.060426028	2.06638305	8.89E-07	2.30E-05	C17orf107	protein_coding	2237
ENSG00000054967	1.058813486	1.952325125	9.59E-05	0.001332226	RELT	protein_coding	2669
ENSG00000171872	1.058599544	-0.090648225	0.003015468	0.021570751	KLF17	protein_coding	1913
ENSG00000112972	1.058420633	7.051063965	1.14E-28	5.35E-26	HMGCS1	protein_coding	708
ENSG00000126562	1.056592968	4.647030446	4.77E-07	1.32E-05	WNK4	protein_coding	1099.5
ENSG00000280143	1.051679959	3.770145861	2.78E-06	6.35E-05	AP000892.6	TEC	5326
ENSG00000111799	1.051303567	11.29421877	2.56E-18	4.10E-16	COL12A1	protein_coding	3135
ENSG00000164112	1.049279508	-0.196237634	0.004892589	0.031446503	TMEM155	protein_coding	1377.5
ENSG00000068078	1.048832775	-0.308176146	0.001628366	0.013318261	FGFR3	protein_coding	4041.5
ENSG00000101825	1.048311954	9.123279287	1.48E-25	4.64E-23	MXRA5	protein_coding	9793
ENSG00000256894	1.047315654	0.239955919	0.000459304	0.004828163	RP11-283G6.3	antisense	501
ENSG00000148655	1.046306882	0.874817227	2.68E-05	0.00044893	C10orf11	protein_coding	373
ENSG00000064547	1.045192822	0.379329336	0.006141037	0.037437365	LPAR2	protein_coding	595
ENSG00000178187	1.04155497	-0.304306434	0.006537996	0.03925641	ZNF454	protein_coding	2142
ENSG00000188766	1.041279635	2.53491548	1.10E-05	0.000208554	SPRED3	protein_coding	646
ENSG00000176697	1.04032189	3.618293158	1.44E-06	3.51E-05	BDNF	protein_coding	4030
ENSG00000165757	1.039436826	7.459796388	3.53E-13	3.04E-11	KIAA1462	protein_coding	684
ENSG00000140743	1.033413263	5.59559517	2.77E-22	6.62E-20	CDR2	protein_coding	711
ENSG00000140092	1.028245575	7.818086559	1.27E-22	3.20E-20	FBLN5	protein_coding	900
ENSG00000122335	1.024889922	5.293303881	3.59E-13	3.07E-11	SERAC1	protein_coding	2284
ENSG00000179348	1.023113723	1.789561908	0.00031517	0.003568554	GATA2	protein_coding	1655.5
ENSG00000134802	1.019770182	4.154350521	4.24E-10	2.24E-08	SLC43A3	protein_coding	588
ENSG00000231760	1.018464862	-0.425155623	0.003055128	0.021786723	RP11-350J20.5	antisense	882
ENSG00000108960	1.017741733	2.788665166	3.31E-06	7.36E-05	MMD	protein_coding	670
ENSG00000143162	1.0152292	5.317869672	1.57E-21	3.42E-19	CREG1	protein_coding	1384
ENSG00000095951	1.009772335	5.096169272	4.33E-16	5.68E-14	HIVEP1	protein_coding	1602
ENSG00000132465	1.007996039	1.71448112	7.34E-05	0.001066652	JCHAIN	protein_coding	586.5
ENSG00000260196	1.005319398	0.584747004	0.001797234	0.014470808	RP1-239B22.5	antisense	2883
ENSG00000151014	1.005151647	3.446607551	3.98E-06	8.62E-05	NOCT	protein_coding	1572
ENSG00000136383	1.003443201	1.312731398	0.001242363	0.010802079	ALPK3	protein_coding	5702
ENSG00000066248	-1.000709336	0.439293707	0.000652644	0.006360287	NGEF	protein_coding	590
ENSG00000136274	-1.001723004	1.683574405	2.27E-06	5.35E-05	NACAD	protein_coding	2554
ENSG00000164010	-1.002579094	3.914739168	0.0001267	0.001684101	ERMAP	protein_coding	3369
ENSG00000177406	-1.003690409	2.223251896	2.23E-05	0.000381703	RP11-218M22.1	antisense	2194
ENSG00000176826	-1.003955542	2.672297973	0.000202171	0.00245126	FKBP9P1	transcribed_unprocessed_pseudogene	1971
ENSG00000250510	-1.004287996	0.567601778	0.005165067	0.032820915	GPR162	protein_coding	1150
ENSG00000261327	-1.006282546	1.114556887	0.000139567	0.001825838	RP11-863P13.3	lincRNA	1034.5
ENSG00000240050	-1.007464398	-0.035351161	0.003083563	0.021949388	RP1-93H18.1	lincRNA	1055
ENSG00000166321	-1.008106452	0.721056373	0.00355211	0.024406319	NUDT13	protein_coding	2010.5
ENSG00000182621	-1.008293158	2.59303357	2.81E-10	1.54E-08	PLCB1	protein_coding	2055
ENSG00000161267	-1.011310271	0.859748311	0.000163739	0.002076463	BDH1	protein_coding	680
ENSG00000158813	-1.011859632	-0.132189834	0.007272754	0.042354181	EDA	protein_coding	1167
ENSG00000106948	-1.015374572	4.67406341	0.003725343	0.025352314	AKNA	protein_coding	3894
ENSG00000153234	-1.01616925	0.383568784	0.000529111	0.005382985	NR4A2	protein_coding	2000
ENSG00000167065	-1.020872408	2.721290177	9.50E-12	6.56E-10	DUSP18	protein_coding	1231.5
ENSG00000131019	-1.020995289	1.004393944	0.000106446	0.001463744	ULBP3	protein_coding	861.5
ENSG00000144730	-1.021535121	2.084304702	2.97E-07	8.73E-06	IL17RD	protein_coding	2167

ENSG00000234814	-1.024862327	1.136019943	0.00017565	0.002195248	SVILP1	transcribed_unprocessed_pseudogene	525
ENSG00000183762	-1.025560218	3.724385574	7.97E-14	7.43E-12	KREMEN1	protein_coding	997
ENSG00000187796	-1.026701215	0.842945625	0.00117877	0.010340146	CARD9	protein_coding	1912
ENSG00000257815	-1.027606617	0.007107253	0.001479051	0.012380628	LINC01481	antisense	512.5
ENSG00000091409	-1.02887334	2.066020366	3.51E-06	7.74E-05	ITGA6	protein_coding	970
ENSG00000262049	-1.03088375	0.225051169	0.001450298	0.012199117	RP13-1032I1.7	antisense	1977
ENSG00000132718	-1.033128142	5.207907069	1.15E-18	1.94E-16	SYT11	protein_coding	5240
ENSG00000072818	-1.035843874	0.524373138	0.004251068	0.028180333	ACAP1	protein_coding	603.5
ENSG00000010438	-1.03593902	0.074100352	0.002494855	0.01864524	PRSS3	protein_coding	704.5
ENSG00000159214	-1.036723308	1.063894045	0.001407046	0.011942018	CCDC24	protein_coding	860
ENSG00000140939	-1.037345321	1.904536911	0.000250345	0.002933848	NOL3	protein_coding	677
ENSG00000255857	-1.039000306	0.512543066	6.19E-05	0.000920065	PXN-AS1	antisense	767
ENSG00000168781	-1.039797441	2.067928512	3.96E-06	8.57E-05	PPIP5K1	protein_coding	777
ENSG00000137133	-1.040765412	3.079977742	3.48E-13	3.01E-11	HINT2	protein_coding	717
ENSG00000221887	-1.04114537	-0.281352179	0.004466692	0.02928165	HMSD	protein_coding	612.5
ENSG00000248587	-1.041920837	0.574306942	0.000289181	0.003312844	GDNF-AS1	antisense	1941
ENSG00000259353	-1.042072324	-0.62272832	0.006963461	0.041096127	RP11-30K9.5	antisense	450
ENSG00000187860	-1.042374869	0.372450843	0.006778466	0.040296932	CCDC157	protein_coding	1759
ENSG00000247400	-1.043183438	0.026197206	0.001000698	0.009073965	DNAJC3-AS1	lincRNA	1654.5
ENSG00000137727	-1.045630454	3.724748946	3.18E-07	9.22E-06	ARHGAP20	protein_coding	6032
ENSG00000185215	-1.045655904	4.447106379	2.35E-06	5.51E-05	TNFAIP2	protein_coding	1334
ENSG00000259721	-1.046564234	2.288838937	1.09E-10	6.32E-09	RP11-758N13.1	lincRNA	1156
ENSG00000247373	-1.046622972	0.295633198	0.006279283	0.038111863	RP11-486O12.2	lincRNA	8279
ENSG00000163945	-1.047426104	2.793128937	0.004169157	0.027779622	UVSSA	protein_coding	2132.5
ENSG00000163888	-1.052647639	-0.275402534	0.001887618	0.015013594	CAMK2N2	protein_coding	1362
ENSG00000272398	-1.053726445	0.785315781	0.002032011	0.015914127	CD24	protein_coding	813.5
ENSG00000237399	-1.056494972	-0.167510284	0.004011042	0.026881769	PITRM1-AS1	antisense	601
ENSG00000271646	-1.057390331	0.162528855	0.00224224	0.017172539	RP11-326I11.3	lincRNA	2503
ENSG00000271614	-1.058122792	0.320206538	0.000398044	0.004289553	LINC00936	lincRNA	3632
ENSG00000166292	-1.059706438	1.54716832	8.78E-08	2.90E-06	TMEM100	protein_coding	645
ENSG00000065060	-1.062738075	4.921475424	7.66E-12	5.42E-10	UHRF1BP1	protein_coding	7138
ENSG00000181218	-1.065256575	0.39634884	0.00083208	0.007811218	HIST3H2A	protein_coding	895
ENSG00000134574	-1.065257576	4.43331671	1.16E-08	4.69E-07	DDB2	protein_coding	928.5
ENSG00000104883	-1.065572192	0.671841072	0.008059392	0.045701517	PEX11G	protein_coding	949
ENSG00000159261	-1.066108273	0.271673714	0.007528446	0.043452225	CLDN14	protein_coding	1391.5
ENSG00000162999	-1.067420504	1.586789024	0.003130439	0.022201463	DUSP19	protein_coding	1117
ENSG00000161243	-1.070225938	0.619516458	0.000484894	0.005024367	FBXO27	protein_coding	771
ENSG00000196912	-1.070738361	0.834899474	5.22E-05	0.000802732	ANKRD36B	protein_coding	1504
ENSG00000123610	-1.070810512	3.508412844	1.48E-09	7.23E-08	TNFAIP6	protein_coding	962.5
ENSG00000110031	-1.07085702	4.892981138	1.45E-14	1.57E-12	LPXN	protein_coding	1833
ENSG00000260231	-1.071541218	0.943384407	0.000374869	0.004096652	JHDM1D-AS1	antisense	2380
ENSG00000223478	-1.07251579	-0.177740326	0.001574517	0.012980362	RP11-545E17.3	antisense	544
ENSG00000175832	-1.07327734	1.245682907	0.002876917	0.020798548	ETV4	protein_coding	1628
ENSG00000181195	-1.073971861	1.133195818	4.32E-05	0.000682844	PENK	protein_coding	606
ENSG00000273008	-1.074083038	-0.396729845	0.004932066	0.031611933	RP11-351D16.3	lincRNA	1511
ENSG00000048540	-1.074418683	1.444057738	0.000357154	0.003947618	LMO3	protein_coding	983
ENSG00000221926	-1.075092808	3.072704776	6.89E-12	4.95E-10	TRIM16	protein_coding	568
ENSG00000273729	-1.075407093	0.030327508	0.004218435	0.028044806	RP11-7F17.8	antisense	3487
ENSG00000140961	-1.076683405	0.890992743	9.66E-05	0.001339542	OSGIN1	protein_coding	833
ENSG00000235863	-1.077311704	1.315599382	0.000128786	0.001706352	B3GALT4	protein_coding	1247
ENSG00000274012	-1.077737319	3.582777206	1.44E-06	3.52E-05	RN7SL2	misc_RNA	300
ENSG00000279453	-1.078213975	1.369222412	5.32E-07	1.45E-05	RP3-425C14.4	TEC	2435
ENSG00000185862	-1.079330109	0.946141395	0.006209867	0.037779121	EVI2B	protein_coding	1798
ENSG00000160200	-1.080706275	1.936113694	0.000485814	0.005027844	CBS	protein_coding	828
ENSG00000225264	-1.085209181	0.239657817	0.006844332	0.040561384	ZNRF2P2	transcribed_processed_pseudogene	590
ENSG00000271780	-1.086600551	-0.814996151	0.008721175	0.048316428	RP11-1017G21.5	lincRNA	1079
ENSG00000188015	-1.088110914	1.32610277	5.68E-08	1.96E-06	S100A3	protein_coding	717.5
ENSG00000175040	-1.088443503	3.002393446	3.00E-09	1.39E-07	CHST2	protein_coding	4582
ENSG00000196917	-1.088857911	0.184374344	0.004822082	0.031109192	HCAR1	protein_coding	4768
ENSG00000214575	-1.089403917	2.340411809	5.30E-05	0.000811505	CPEB1	protein_coding	2038.5
ENSG00000152953	-1.090269859	2.080640357	1.22E-07	3.87E-06	STK32B	protein_coding	1481
ENSG00000187134	-1.092635772	4.055413164	1.29E-11	8.59E-10	AKR1C1	protein_coding	858
ENSG00000175643	-1.096115139	2.926505501	4.45E-07	1.24E-05	RMI2	protein_coding	934
ENSG00000198342	-1.097202452	0.550119676	0.000222714	0.002661387	ZNF442	protein_coding	1884
ENSG00000163701	-1.098594589	-0.032358274	0.000818486	0.007703628	IL17RE	protein_coding	2011
ENSG00000227946	-1.099149835	0.264803316	0.000571121	0.005732201	AC007383.3	lincRNA	617
ENSG00000185567	-1.099169275	6.703288468	1.25E-15	1.60E-13	AHNAK2	protein_coding	10845
ENSG00000227199	-1.099867468	-0.160222142	0.000500975	0.005153846	ST7-AS1	antisense	1889
ENSG00000104419	-1.100831013	6.739280914	4.57E-32	3.05E-29	NDRG1	protein_coding	584
ENSG00000266709	-1.101482799	1.165887678	0.000183722	0.0022769	RP11-214O1.2	lincRNA	1652
ENSG00000247809	-1.101901601	1.057024719	5.11E-05	0.000789249	NR2F2-AS1	antisense	549
ENSG00000112137	-1.102924387	-0.109069218	0.001297434	0.011199899	PHACTR1	protein_coding	776
ENSG00000256594	-1.103764727	2.077112054	2.11E-07	6.39E-06	RP11-705C15.2	transcribed_unprocessed_pseudogene	805
ENSG00000008300	-1.104692826	0.562043249	0.001887924	0.015013594	CELSR3	protein_coding	3633
ENSG00000099875	-1.106441204	5.969060633	3.62E-09	1.64E-07	MKNK2	protein_coding	1135
ENSG00000259807	-1.10948653	0.74412369	0.005159954	0.032798493	RP11-426C22.4	lincRNA	2099
ENSG00000197696	-1.110139642	0.888851029	0.000398423	0.00429066	NMB	protein_coding	1012
ENSG00000143344	-1.112444533	4.675233885	8.21E-24	2.27E-21	RGL1	protein_coding	4912
ENSG00000157514	-1.115006699	4.410408573	2.47E-05	0.000418713	TSC22D3	protein_coding	581
ENSG00000163393	-1.115651086	3.212166494	0.001823652	0.014637992	SLC22A15	protein_coding	1230

ENSG00000266265	-1.117101209	-0.508394261	0.001796716	0.014470808	KLF14	protein_coding	2827
ENSG00000269906	-1.11718602	-0.452167564	0.004236952	0.028137397	RP11-248J18.2	sense_intronic	668
ENSG00000057657	-1.118512221	3.057737265	6.75E-09	2.88E-07	PRDM1	protein_coding	649
ENSG00000278530	-1.120058454	-0.013256476	0.003781762	0.025618747	CHMP1B2P	transcribed_unitary_pseudogene	3705
ENSG00000133460	-1.120726613	1.67090166	0.000709025	0.006843104	SLC2A11	protein_coding	1048
ENSG00000165171	-1.124260744	0.892830698	0.00036644	0.004025784	WBSCR27	protein_coding	823
ENSG00000176909	-1.124712588	1.1996824	9.68E-06	0.000187408	MAMSTR	protein_coding	1693
ENSG00000158406	-1.128883133	0.532121252	0.000179442	0.002237054	HIST1H4H	protein_coding	1034
ENSG00000266865	-1.129400191	1.619997178	0.000584848	0.005828495	RP11-848P1.9	transcribed_unprocessed_pseudogene	534
ENSG00000089091	-1.12999342	0.803044589	0.001071269	0.009551072	DZANK1	protein_coding	3156
ENSG00000228451	-1.131236643	1.323014487	8.24E-05	0.001178807	SDAD1P1	transcribed_processed_pseudogene	2612.5
ENSG00000261499	-1.131850276	-0.729729568	0.009108867	0.049956441	CH17-260O16.1	unprocessed_pseudogene	1168
ENSG00000140876	-1.1354117	-0.599621748	0.00568246	0.035222407	NUDT7	protein_coding	899
ENSG00000128510	-1.136851256	2.247277161	4.38E-05	0.000690771	CPA4	protein_coding	568
ENSG00000249572	-1.137848054	-0.523679042	0.003554078	0.024411739	CTD-2203K17.1	antisense	537
ENSG00000165105	-1.140686348	0.608934857	0.000473418	0.004934075	RASEF	protein_coding	3670.5
ENSG00000283689	-1.142788523	0.663401895	0.000384992	0.00418081	RP11-434E6.5	antisense	1064
ENSG00000231445	-1.143804769	-0.139423177	0.006420827	0.038766444	TIMM8AP1	processed_pseudogene	295
ENSG00000171246	-1.145240699	0.200959689	0.002769396	0.020187052	NPTX1	protein_coding	2256.5
ENSG00000149582	-1.148160378	1.599509725	2.61E-05	0.000439249	TMEM25	protein_coding	701.5
ENSG00000232818	-1.148415597	-0.242698245	0.000207612	0.002506964	RPS2P32	processed_pseudogene	892
ENSG00000254473	-1.149903406	0.270985774	0.001972409	0.015556053	RP11-522I20.3	antisense	753
ENSG00000151617	-1.150293562	1.765263907	1.27E-10	7.27E-09	EDNRA	protein_coding	1569
ENSG00000242147	-1.150966657	0.12298628	0.00451903	0.029577939	RP13-463N16.6	lincRNA	697
ENSG00000223797	-1.15169556	-0.332987887	0.002685797	0.019723463	ENTPD3-AS1	antisense	757.5
ENSG00000246763	-1.15340424	1.39371781	0.002516877	0.018769998	RGMB-AS1	antisense	533
ENSG00000174125	-1.153518425	-0.92939593	0.007990138	0.045483166	TLR1	protein_coding	612
ENSG00000213888	-1.153800868	0.281960911	0.000351129	0.003890598	LINC01521	lincRNA	1232
ENSG00000205683	-1.154667067	2.814887711	2.10E-05	0.000363094	DPF3	protein_coding	853.5
ENSG00000235954	-1.155579142	0.308295092	0.003830974	0.025909744	TTC28-AS1	processed_transcript	706
ENSG00000235865	-1.156201202	0.142684032	0.008965134	0.049377385	GSN-AS1	antisense	4763
ENSG00000232973	-1.156755792	0.73084897	0.000288414	0.003307177	CYP1B1-AS1	antisense	612
ENSG00000259802	-1.164891715	-0.959397769	0.005198129	0.033010749	CTD-2256P15.2	antisense	901
ENSG00000187650	-1.168032071	1.365420865	0.001070555	0.009551072	VMAC	protein_coding	2254
ENSG00000172824	-1.175002902	0.546944619	0.002282013	0.017432022	CES4A	protein_coding	1825
ENSG00000161653	-1.175680689	-0.378827414	0.00253139	0.018857031	NAGS	protein_coding	1995
ENSG00000067606	-1.176053748	0.294165033	5.67E-05	0.000854864	PRKCZ	protein_coding	702
ENSG00000272994	-1.17606568	-0.128019829	0.002094389	0.016268832	RP11-332H14.2	lincRNA	3449
ENSG00000204428	-1.177678223	-0.894067676	0.008021559	0.045549345	LY6G5C	protein_coding	674
ENSG00000105327	-1.178406969	3.619492351	7.59E-11	4.50E-09	BBC3	protein_coding	1538
ENSG00000198835	-1.178608441	0.487135291	0.000182961	0.002269788	GJC2	protein_coding	2243
ENSG00000237489	-1.182464402	0.745332313	0.000575823	0.005769099	LINC00959	protein_coding	1834.5
ENSG00000215790	-1.18247417	1.455719639	0.000378437	0.004120423	SLC35E2	protein_coding	1430
ENSG00000275765	-1.183731222	-0.098973984	0.002172093	0.01674679	RP11-54C4.3	lincRNA	726
ENSG00000196812	-1.183762642	-0.700909376	0.007314555	0.042537805	ZSCAN16	protein_coding	1328
ENSG00000145536	-1.184057111	0.844518919	0.001989974	0.015651492	ADAMTS16	protein_coding	2954.5
ENSG00000221821	-1.185940532	1.332704824	2.74E-06	6.27E-05	C6orf226	protein_coding	552
ENSG00000265817	-1.18673342	-0.490020713	0.007487401	0.043287711	FSBP	protein_coding	1815
ENSG00000168646	-1.187291062	3.201046075	6.10E-15	7.06E-13	AXIN2	protein_coding	1034
ENSG00000137198	-1.188052084	-0.032558036	0.000539722	0.005473488	GMPR	protein_coding	771.5
ENSG00000149798	-1.191815024	4.244719595	1.45E-23	3.96E-21	CDC42EP2	protein_coding	1651
ENSG00000059377	-1.193506367	-0.34499285	0.000726043	0.006977088	TBXAS1	protein_coding	1435
ENSG00000116584	-1.194267659	7.061549984	6.77E-06	0.000138293	ARHGEF2	protein_coding	792
ENSG00000267100	-1.195397938	2.167503509	3.33E-10	1.77E-08	ILF3-AS1	lincRNA	1983
ENSG00000275993	-1.195572357	1.301684442	0.000340044	0.003786002	CH507-42P11.8	protein_coding	2588
ENSG00000164185	-1.195730108	-0.762031251	0.003985299	0.0267352	ZNF474	protein_coding	572
ENSG00000144218	-1.200394517	1.202194771	0.002010876	0.015785909	AFF3	protein_coding	591
ENSG00000135069	-1.200871755	7.751751344	7.52E-12	5.34E-10	PSAT1	protein_coding	1799.5
ENSG00000247092	-1.20089152	1.021477172	0.000223438	0.002666362	SNHG10	antisense	704.5
ENSG00000257027	-1.202353882	0.467732389	3.82E-05	0.000613675	RP11-705C15.3	sense_intronic	2508
ENSG00000213920	-1.202489543	-0.062110449	0.001426352	0.012055087	MDP1	protein_coding	578
ENSG00000184786	-1.210368101	-0.653400345	0.002352932	0.017816039	TCTE3	protein_coding	1423.5
ENSG00000260563	-1.212027132	-0.623620266	0.008462051	0.047285651	RP13-516M14.1	lincRNA	1195
ENSG00000260114	-1.212155401	0.018728135	0.007317707	0.042544185	CTD-2574D22.4	sense_intronic	2272
ENSG00000256073	-1.216268973	0.617288384	1.75E-05	0.000311202	URB1-AS1	lincRNA	831
ENSG00000231711	-1.216614812	0.821892658	0.000737995	0.007059186	LINC00899	processed_transcript	2584
ENSG00000175567	-1.220520756	0.365144983	0.000194869	0.002382254	UCP2	protein_coding	552.5
ENSG00000169282	-1.221825523	2.795231797	5.80E-12	4.21E-10	KCNAB1	protein_coding	580
ENSG00000196754	-1.222364111	-0.326846244	0.002304383	0.017544445	S100A2	protein_coding	626.5
ENSG00000157873	-1.222703027	2.360835609	0.000962782	0.008778199	TNFRSF14	protein_coding	884
ENSG00000214756	-1.223364209	1.152780406	8.62E-07	2.23E-05	METTL12	protein_coding	1702.5
ENSG00000261737	-1.226065686	-0.598988788	0.00084539	0.007925389	RP4-612B15.3	antisense	961
ENSG00000129194	-1.23125843	-0.806570508	0.003530396	0.024305506	SOX15	protein_coding	1093
ENSG00000235374	-1.238528532	0.580700282	0.00184534	0.014751212	SSR4P1	transcribed_processed_pseudogene	967
ENSG00000230918	-1.240338378	-0.026775592	0.005455923	0.034238394	AC008063.2	antisense	640
ENSG00000106336	-1.243582375	-0.871244307	0.00721083	0.04208858	FBXO24	protein_coding	1330
ENSG00000186364	-1.2441723	0.95498896	4.20E-05	0.00066659	NUDT17	protein_coding	873
ENSG00000229124	-1.244677973	-0.269923766	0.000189737	0.002326386	VIM-AS1	antisense	1134.5
ENSG00000232442	-1.245850562	0.613697096	2.17E-05	0.00037283	CTD-3184A7.4	antisense	691.5
ENSG00000235109	-1.251580328	1.727048454	0.000956717	0.008743447	ZSCAN31	protein_coding	706

ENSG00000174611	-1.251917528	-0.150896254	0.001008448	0.009107697	KY	protein_coding	2345
ENSG00000130943	-1.252857521	-0.867162093	0.008417183	0.047123936	PKDREJ	protein_coding	7693
ENSG00000174370	-1.257905381	1.431705985	5.38E-06	0.000112688	C11orf45	protein_coding	609
ENSG00000176438	-1.261122726	3.86441469	1.87E-08	7.16E-07	SYNE3	protein_coding	3275
ENSG00000221890	-1.262111921	0.831876716	4.73E-06	0.000100834	NPTXR	protein_coding	5784
ENSG00000105514	-1.263455564	1.596197417	8.22E-08	2.75E-06	RAB3D	protein_coding	2598.5
ENSG00000205791	-1.267682022	-0.2278238	0.001446142	0.0121691	LOH12CR2	lincRNA	1540
ENSG00000168916	-1.267878477	1.330231817	0.005237569	0.033210294	ZNF608	protein_coding	2645.5
ENSG00000261455	-1.268098785	0.663104015	5.15E-07	1.41E-05	LINC01003	lincRNA	1764
ENSG00000214193	-1.269618036	1.38542925	0.003904776	0.026297237	SH3D21	protein_coding	2043
ENSG00000153879	-1.269654814	5.309420291	2.34E-06	5.49E-05	CEBPG	protein_coding	3977
ENSG00000168621	-1.270966714	3.154577387	2.26E-11	1.45E-09	GDNF	protein_coding	817
ENSG00000280187	-1.271953968	1.257817949	3.61E-07	1.03E-05	CTC-351M12.1	TEC	3094
ENSG00000228544	-1.274163714	-0.210493435	0.001141197	0.010048069	CCDC183-AS1	antisense	2386
ENSG00000159917	-1.274782752	0.24466868	0.001472714	0.012337551	ZNF235	protein_coding	955
ENSG00000196517	-1.277935998	4.659403436	1.43E-05	0.000259887	SLC6A9	protein_coding	1881
ENSG00000272523	-1.278308416	-0.045292702	0.000289228	0.003312844	LINC01023	lincRNA	436
ENSG00000267216	-1.279267457	-1.213024691	0.007210888	0.04208858	AC010642.1	protein_coding	4227
ENSG00000224086	-1.279780408	2.301176109	2.73E-06	6.25E-05	LL22NC03-86G7.1	antisense	37852
ENSG00000132623	-1.280463903	-0.250641234	0.000378846	0.004122716	ANKEF1	protein_coding	2849.5
ENSG00000172061	-1.280767313	3.121749265	2.04E-13	1.81E-11	LRRC15	protein_coding	4565.5
ENSG00000197935	-1.281055384	0.437513761	0.004433381	0.02910012	ZNF311	protein_coding	2878.5
ENSG00000254531	-1.283753686	1.529983746	1.34E-07	4.22E-06	FLJ20021	lincRNA	616
ENSG00000188064	-1.285240606	3.092264888	0.002296113	0.017504685	WNT7B	protein_coding	2202
ENSG00000223745	-1.287868282	1.754477377	0.009083545	0.049870396	CCDC18-AS1	processed_transcript	513.5
ENSG00000106123	-1.287998602	-0.391810631	0.004829554	0.031141587	EPHB6	protein_coding	3224
ENSG00000274712	-1.288147204	-0.300596159	0.007999809	0.045494205	RP11-147L13.15	lincRNA	2005
ENSG00000168528	-1.288156843	3.95986454	3.98E-19	7.03E-17	SERINC2	protein_coding	2067
ENSG00000231672	-1.28883034	-0.357637813	0.001344125	0.011496726	DIRC3	protein_coding	1621
ENSG00000074935	-1.2898145	4.379101366	0.000472348	0.004927882	TUBE1	protein_coding	726
ENSG00000197119	-1.291062081	1.953544622	7.73E-06	0.000153243	SLC25A29	protein_coding	776.5
ENSG00000170891	-1.291215852	-0.247266611	0.002746047	0.020052148	CYTL1	protein_coding	553
ENSG00000279086	-1.291525136	-0.259717785	0.000723323	0.006954173	RP11-667F14.1	TEC	2278
ENSG00000280138	-1.293155817	-0.221268967	0.000581983	0.005811129	RP11-463O12.5	TEC	18662
ENSG00000164850	-1.29349166	3.617667423	8.07E-08	2.70E-06	GPER1	protein_coding	1900
ENSG00000166046	-1.295250034	3.326397363	4.19E-05	0.000664318	TCP11L2	protein_coding	805
ENSG00000188596	-1.297146937	0.035102923	0.000282755	0.003256349	CFAP54	protein_coding	2323
ENSG00000259877	-1.30287559	0.351483526	2.50E-05	0.000422292	RP11-46C24.7	antisense	2443
ENSG00000162997	-1.304324496	-0.91350356	0.00902726	0.049679918	PRORSD1P	transcribed_unitary_pseudogene	626
ENSG00000115257	-1.305016648	-0.292576113	0.002862296	0.020725623	PCSK4	protein_coding	720
ENSG00000188825	-1.306283565	0.66342708	0.000425262	0.004537225	LINC00910	lincRNA	1672.5
ENSG00000263874	-1.308455593	-0.492993876	0.002736627	0.019997464	LINC00672	protein_coding	3692
ENSG00000114626	-1.316927391	3.031015957	2.66E-07	7.89E-06	ABTB1	protein_coding	818
ENSG00000174343	-1.320225643	-1.138859149	0.003115415	0.022110052	CHRNA9	protein_coding	622
ENSG00000182796	-1.320490259	2.613211258	0.003414966	0.023715805	TMEM198B	transcribed_unitary_pseudogene	633
ENSG00000232977	-1.320700725	-0.233442008	0.000139267	0.001823056	LINC00327	lincRNA	1308
ENSG00000173253	-1.320833732	-0.9632969	0.008551887	0.047633412	DMRT2	protein_coding	1965
ENSG00000167191	-1.321034704	3.576613952	1.79E-05	0.0003174	GPRC5B	protein_coding	818
ENSG00000172927	-1.322531472	0.578833743	3.37E-05	0.000550052	MYEOV	protein_coding	616
ENSG00000183840	-1.322537686	0.485966632	2.65E-06	6.10E-05	GPR39	protein_coding	2013
ENSG00000134242	-1.322720084	1.655127532	4.57E-12	3.36E-10	PTPN22	protein_coding	2258
ENSG00000114251	-1.323962614	7.14582391	6.82E-31	4.15E-28	WNT5A	protein_coding	894
ENSG00000244586	-1.324406953	2.267182692	1.83E-09	8.83E-08	WNT5A-AS1	antisense	500
ENSG00000137098	-1.32500418	-0.946018525	0.006689908	0.039900622	SPAG8	protein_coding	920.5
ENSG00000165879	-1.32722613	0.402864777	2.04E-06	4.84E-05	FRAT1	protein_coding	1641
ENSG00000122432	-1.327756217	-0.878031001	0.004448088	0.029187403	SPATA1	transcribed_unprocessed_pseudogene	776
ENSG00000260236	-1.327842812	-0.460729399	0.000946966	0.008677308	RP11-708J19.1	antisense	1911
ENSG00000126950	-1.329940021	0.574092006	0.001685985	0.013740679	TMEM35A	protein_coding	1469
ENSG00000156689	-1.330377296	-0.100504574	4.30E-05	0.000680474	GLYATL2	protein_coding	1488
ENSG00000189014	-1.33230967	-1.013939531	0.008103416	0.045800667	FAM35DP	unprocessed_pseudogene	2712
ENSG00000162522	-1.332440626	4.524340263	9.48E-25	2.84E-22	KIAA1522	protein_coding	5293
ENSG00000196611	-1.332457064	3.793443826	7.29E-15	8.29E-13	MMP1	protein_coding	1970
ENSG00000182359	-1.333940093	0.944106125	0.000750088	0.007164936	KBTBD3	protein_coding	874
ENSG00000107611	-1.334150875	2.140166546	1.39E-06	3.41E-05	CUBN	protein_coding	831.5
ENSG00000271882	-1.337118984	-0.182233902	0.001080009	0.009611473	KB-1410C5.5	lincRNA	411
ENSG00000102048	-1.346891473	-0.556677142	0.000481345	0.004996583	ASB9	protein_coding	1153
ENSG00000245522	-1.348043175	0.942809428	7.96E-05	0.001143173	RP11-540A21.2	lincRNA	2239.5
ENSG00000138678	-1.348075198	-0.762715107	0.000538511	0.005466676	GPAT3	protein_coding	647
ENSG00000255364	-1.348945911	-0.680801868	0.001825303	0.014645562	RP11-94A24.1	lincRNA	553
ENSG00000256591	-1.352974665	-1.013756245	0.007835644	0.044837895	RP11-286N22.8	protein_coding	585.5
ENSG00000138030	-1.355688266	-0.801100043	0.001913104	0.015185308	KHK	protein_coding	1349.5
ENSG00000259605	-1.356396037	-0.123449822	0.000280127	0.003228242	AC074212.5	processed_transcript	1411
ENSG00000226696	-1.356915326	-0.125078125	0.004427748	0.029072363	LENG8-AS1	antisense	582
ENSG00000270021	-1.362655688	-0.505558959	0.001573729	0.012979034	CTC-203F4.2	antisense	2017
ENSG00000184363	-1.364474914	1.24287443	1.04E-06	2.65E-05	PKP3	protein_coding	687
ENSG00000246273	-1.375411375	1.411791031	9.31E-08	3.03E-06	SBF2-AS1	antisense	716.5
ENSG00000184368	-1.375883299	0.236320158	2.17E-05	0.000373466	MAP7D2	protein_coding	2476
ENSG00000198576	-1.376516231	0.122266704	0.000173457	0.002175728	ARC	protein_coding	1677.5
ENSG00000247950	-1.382529521	-0.915699646	0.003206334	0.022583159	SEC24B-AS1	antisense	878.5
ENSG00000245317	-1.383690103	-0.844051008	0.00469767	0.030506133	CTC-241N9.1	lincRNA	1454

ENSG00000231652	-1.38371323	-0.328154589	3.00E-05	0.000495585	RP11-553A21.3	antisense	1973
ENSG00000154133	-1.393533628	1.114410418	2.25E-05	0.000384694	ROBO4	protein_coding	752.5
ENSG00000175611	-1.397096236	0.675924829	8.44E-07	2.19E-05	LINC00476	processed_transcript	966.5
ENSG00000260329	-1.397612138	0.160894911	1.61E-05	0.000289224	RP11-412D9.4	antisense	1469
ENSG00000250222	-1.404273772	-0.965620179	0.00859528	0.047797974	CTC-338M12.5	antisense	340.5
ENSG00000167550	-1.409055085	-0.900628496	0.007984135	0.045473994	RHEBL1	protein_coding	1136.5
ENSG00000163814	-1.410146508	3.141694172	7.37E-06	0.000147552	CDCP1	protein_coding	1416
ENSG00000269403	-1.411156633	-1.139815602	0.00318214	0.022460443	CTD-2616J11.11	protein_coding	551
ENSG00000273253	-1.412251785	-0.940483358	0.002959852	0.021305379	RP3-402G11.26	antisense	1001
ENSG00000166165	-1.412784316	2.019876111	1.64E-07	5.08E-06	CKB	protein_coding	746
ENSG00000272501	-1.41318515	-0.269764068	0.007622926	0.043887457	XXbac-BPG299F13.17	antisense	2838
ENSG00000146733	-1.413778112	4.998196828	1.07E-12	8.49E-11	PSPH	protein_coding	741.5
ENSG00000268996	-1.416978896	0.812110393	2.93E-06	6.66E-05	MAN1B1-AS1	antisense	1872
ENSG00000103241	-1.417382314	1.772002777	7.61E-06	0.000151643	FOXF1	protein_coding	3516
ENSG00000090661	-1.419019781	-0.755856588	0.007527268	0.043452225	CERS4	protein_coding	763
ENSG00000141682	-1.419976841	3.373378995	4.17E-06	8.97E-05	PMAIP1	protein_coding	1262
ENSG00000260018	-1.42029116	-0.871474057	0.000812909	0.007662241	RP11-505K9.1	antisense	521
ENSG00000259744	-1.421957413	-1.653919168	0.008233025	0.046419196	RP11-138H8.6	sense_intronic	755
ENSG00000177076	-1.422048269	-0.19935105	6.19E-05	0.000920065	ACER2	protein_coding	2238
ENSG00000164136	-1.430369014	0.979474778	1.82E-06	4.37E-05	IL15	protein_coding	1417.5
ENSG00000258057	-1.433672139	-0.464829552	0.003385304	0.023541393	BCDIN3D-AS1	antisense	813
ENSG00000101342	-1.435901313	-1.079925053	0.004856186	0.031270757	TLDC2	protein_coding	1448
ENSG00000221990	-1.438366714	1.116779042	7.62E-10	3.91E-08	EXOC3-AS1	antisense	1638.5
ENSG00000268575	-1.44241764	0.481854281	0.003135136	0.022222891	RP1-283E3.8	processed_transcript	5079
ENSG00000226314	-1.44423097	0.189038838	0.004983886	0.031865175	ZNF192P1	transcribed_unprocessed_pseudogene	962
ENSG00000187605	-1.444830684	3.002499052	1.99E-13	1.78E-11	TET3	protein_coding	3391
ENSG00000231908	-1.449820725	-1.208224148	0.002392621	0.018024292	IDH1-AS1	antisense	476.5
ENSG00000232415	-1.452966498	-1.373680499	0.00576848	0.035617013	CTB-51J22.1	antisense	532
ENSG00000235813	-1.453849557	-0.274886375	0.006605798	0.039560284	RP11-308B5.2	lincRNA	938
ENSG00000069482	-1.455252818	-0.213556344	0.001497144	0.012521951	GAL	protein_coding	732
ENSG00000113369	-1.458350402	5.34232051	3.05E-15	3.68E-13	ARRDC3	protein_coding	564
ENSG00000145358	-1.461384991	1.676595206	0.000107466	0.00147482	DDIT4L	protein_coding	852
ENSG00000139597	-1.465378602	-0.090724879	0.004305819	0.028460234	N4BP2L1	protein_coding	1953
ENSG00000102349	-1.467011198	-0.604607702	0.001986048	0.015638665	KLF8	protein_coding	4821
ENSG00000228798	-1.468145315	-0.760592986	0.005700213	0.035269108	AP000473.5	lincRNA	526.5
ENSG00000254876	-1.469028886	0.153961062	0.003475339	0.024030314	RP11-23J9.5	processed_transcript	2368
ENSG00000146950	-1.469700237	0.856172482	0.000148247	0.001910416	SHROOM2	protein_coding	3445.5
ENSG00000124875	-1.471500211	1.973716441	4.90E-14	4.83E-12	CXCL6	protein_coding	557
ENSG00000250067	-1.477872908	0.103200763	0.00607506	0.037131202	YJEFN3	protein_coding	995
ENSG00000146592	-1.478201805	2.260921178	2.74E-14	2.81E-12	CREB5	protein_coding	752
ENSG00000126246	-1.480872813	-1.383495654	0.004470461	0.029297081	IGFLR1	protein_coding	593
ENSG00000172901	-1.489465336	-1.482273551	0.006758849	0.040232551	LVRN	protein_coding	2391
ENSG00000255046	-1.491648387	-1.058767124	0.004699545	0.03050874	RP11-297N6.4	lincRNA	641
ENSG00000139725	-1.493857402	-0.091035222	9.54E-05	0.001326667	RHOF	protein_coding	1219
ENSG00000257038	-1.496421356	-0.150077395	6.12E-05	0.000913811	RP11-800A3.7	antisense	1695
ENSG00000135437	-1.502103044	0.227065156	7.69E-05	0.001111892	RDH5	protein_coding	971
ENSG00000171346	-1.503094836	-0.524947141	0.000164268	0.002081427	KRT15	protein_coding	931
ENSG00000115138	-1.503533451	-0.732519853	0.001004442	0.009087372	POMC	protein_coding	1074
ENSG00000153404	-1.506685871	-1.390871069	0.001003067	0.009086032	PLEKHG4B	protein_coding	6840
ENSG00000280132	-1.506922941	-0.398407705	0.000119537	0.00160789	RP11-452L6.6	TEC	1905
ENSG00000226754	-1.507090582	-0.903493714	0.001369391	0.011675061	RP5-1024G6.5	antisense	1181
ENSG00000213057	-1.507176821	-0.256035772	0.00858718	0.047773526	C1orf220	lincRNA	1576.5
ENSG00000224116	-1.508510197	-1.375033779	0.008968991	0.049385492	INHBA-AS1	antisense	2312
ENSG00000173269	-1.508868119	0.844493778	1.20E-08	4.84E-07	MMRN2	protein_coding	829
ENSG00000237840	-1.50984656	-1.028177754	0.004173812	0.027801694	FAM21FP	transcribed_unprocessed_pseudogene	752
ENSG00000243406	-1.512084288	-0.720273925	0.00320716	0.022583159	MRPS31P5	transcribed_unprocessed_pseudogene	564
ENSG00000187957	-1.515213505	-0.96672219	0.000884831	0.008213338	DNER	protein_coding	2051
ENSG00000205336	-1.515221602	-0.648887878	0.004553159	0.029726249	ADGRG1	protein_coding	579
ENSG00000143061	-1.516209271	-0.915768701	0.000721194	0.006939969	IGSF3	protein_coding	5548
ENSG00000267279	-1.516271348	-0.824443034	0.002286778	0.01745554	RP11-879F14.2	lincRNA	694
ENSG00000160097	-1.517705295	1.682999072	8.94E-05	0.001262452	FNDC5	protein_coding	622
ENSG00000254614	-1.518622981	0.420557778	5.67E-06	0.000117794	AP003068.23	antisense	1069.5
ENSG00000268573	-1.518954027	-0.830247608	0.00011379	0.00154624	RP11-158H5.7	lincRNA	4382
ENSG00000258938	-1.526608935	-0.632637484	0.007898456	0.045116825	RP11-317N8.5	antisense	2867
ENSG00000283050	-1.528559867	0.642529658	0.0014291	0.012055087	GTF2IP12	transcribed_unprocessed_pseudogene	939
ENSG00000144821	-1.529222756	1.645008036	3.40E-07	9.77E-06	MYH15	protein_coding	3207
ENSG00000116761	-1.530396263	3.973170716	7.30E-07	1.92E-05	CTH	protein_coding	1196
ENSG00000260001	-1.531324336	-1.041789368	0.0043279	0.02855247	TGFBR3L	protein_coding	719
ENSG00000002745	-1.532570232	0.212980636	0.003636658	0.024847095	WNT16	protein_coding	2188.5
ENSG00000273702	-1.535203761	-1.243694316	0.009051842	0.049788735	RP11-758H9.2	lincRNA	1162
ENSG00000245281	-1.53544989	-1.092512369	0.001369967	0.011675162	CTD-2547L16.1	antisense	646
ENSG00000227540	-1.537458677	-1.06358351	0.002296597	0.017504685	DNAJC9-AS1	antisense	635
ENSG00000213145	-1.538017426	-0.539388758	0.00022338	0.002666362	CRIP1	protein_coding	994
ENSG00000204291	-1.538235817	3.81900434	1.75E-12	1.35E-10	COL15A1	protein_coding	1412
ENSG00000253878	-1.541261671	-1.240880254	0.00746787	0.043198918	RP11-347C18.3	sense_intronic	579
ENSG00000110455	-1.542165376	2.447554635	0.001308382	0.011284998	ACCS	protein_coding	884
ENSG00000249992	-1.549260531	4.324125841	4.50E-05	0.000706575	TMEM158	protein_coding	1813
ENSG00000130766	-1.558579465	5.826500673	6.05E-08	2.08E-06	SESN2	protein_coding	3453
ENSG00000154153	-1.562488426	-1.234028569	0.00630357	0.038203287	FAM134B	protein_coding	930.5
ENSG00000146054	-1.562791143	-0.729609339	0.001660897	0.013546878	TRIM7	protein_coding	2320

ENSG00000204228	-1.570275054	-0.176063513	7.14E-05	0.001042967	HSD17B8	protein_coding	961.5
ENSG00000134463	-1.574833311	-0.669569346	0.000420475	0.00449309	ECHDC3	protein_coding	854
ENSG00000260868	-1.575867237	-1.636252364	0.002574868	0.019112168	RP11-394I13.1	lincRNA	1884
ENSG00000042062	-1.579037684	0.459103502	0.004462287	0.029262034	FAM65C	protein_coding	1892
ENSG00000212694	-1.579553798	1.534621147	0.000377337	0.00411278	LINC01089	lincRNA	944.5
ENSG00000196932	-1.580595558	1.415927581	0.000387742	0.004200889	TMEM26	protein_coding	2520
ENSG00000178726	-1.581134518	0.855232255	3.47E-05	0.000564339	THBD	protein_coding	4109
ENSG00000200279	-1.585943581	-1.625897481	0.008245065	0.046449141	SNORD114-10	snoRNA	72
ENSG00000139946	-1.587494333	1.129716297	1.42E-12	1.11E-10	PELI2	protein_coding	774
ENSG00000168490	-1.58906489	-0.520741479	0.00019989	0.002427868	PHYHIP	protein_coding	847
ENSG00000168003	-1.589790122	6.569211777	1.24E-14	1.36E-12	SLC3A2	protein_coding	597
ENSG00000273142	-1.591761913	1.032855944	0.003042037	0.021709303	RP11-458F8.4	lincRNA	3441
ENSG00000225511	-1.59177289	0.732921982	0.000153363	0.001964126	LINC00475	lincRNA	589.5
ENSG00000232679	-1.592817671	0.670128747	1.08E-07	3.47E-06	RP11-400N13.3	lincRNA	562
ENSG00000214279	-1.592904401	-0.16692613	0.008843808	0.048821507	SCART1	transcribed_unitary_pseudogene	4080
ENSG00000261879	-1.59389798	-0.516325402	0.003079772	0.021932256	RP11-333E1.1	antisense	554
ENSG00000181885	-1.595481822	-0.268808158	0.000566777	0.005694997	CLDN7	protein_coding	807
ENSG00000234028	-1.595588072	-0.370774499	0.000227427	0.00270366	AC062029.1	antisense	2632.5
ENSG00000172361	-1.596644828	-1.185536208	0.007047492	0.041415016	CFAP53	protein_coding	1851
ENSG00000091137	-1.598828442	0.012860921	0.000580011	0.005802632	SLC26A4	protein_coding	596
ENSG00000258311	-1.601310884	-0.265561618	0.000182918	0.002269788	RP11-644F5.10	protein_coding	884.5
ENSG00000274565	-1.604042035	1.488118668	9.39E-05	0.001310215	CTD-3035K23.7	antisense	1154
ENSG00000234779	-1.606361031	0.235871563	3.11E-09	1.43E-07	RP11-62F24.2	antisense	428
ENSG00000275880	-1.608506227	0.280326754	8.79E-09	3.65E-07	RP11-90L1.8	antisense	3272
ENSG00000261371	-1.608537946	0.347671765	2.23E-09	1.06E-07	PECAM1	protein_coding	580
ENSG00000105339	-1.609915286	1.226608507	6.69E-08	2.27E-06	DENND3	protein_coding	1104.5
ENSG00000262468	-1.61263916	-0.671518573	0.003199552	0.022558144	LINC01569	lincRNA	753
ENSG00000130702	-1.612791347	3.903625887	3.50E-11	2.18E-09	LAMA5	protein_coding	637
ENSG00000275713	-1.616974331	-1.315247017	0.001837083	0.0147059	HIST1H2BH	protein_coding	2060
ENSG00000275719	-1.6198264	-1.116651901	0.001710903	0.013894544	CTB-147N14.6	antisense	1227
ENSG00000223658	-1.620280247	-0.506343137	5.14E-05	0.000792205	C1GALT1C1L	protein_coding	1172
ENSG00000221995	-1.621318366	0.688804475	0.000117644	0.001587166	TIAF1	protein_coding	5337
ENSG00000256124	-1.624700816	-0.568238264	0.006858873	0.04061779	LINC01152	lincRNA	639
ENSG00000275494	-1.631506325	-1.389167447	0.001858283	0.014835459	RP11-266L9.8	sense_intronic	534
ENSG00000159713	-1.633356194	-0.258421197	0.004655313	0.030259533	TPPP3	protein_coding	1116
ENSG00000070669	-1.633789334	6.92907987	4.98E-14	4.88E-12	ASNS	protein_coding	1588
ENSG00000129467	-1.638155495	-0.602775477	0.006357503	0.038451779	ADCY4	protein_coding	897
ENSG00000277969	-1.638842774	-0.878916067	0.000152575	0.001957666	CTB-58E17.1	lincRNA	2296
ENSG00000166106	-1.641210156	2.210231968	3.33E-10	1.77E-08	ADAMTS15	protein_coding	5673
ENSG00000134955	-1.642715478	0.457253689	1.80E-08	6.92E-07	SLC37A2	protein_coding	2642.5
ENSG00000280254	-1.646867135	-0.645626895	1.10E-05	0.000208554	RP11-81A22.4	TEC	2106
ENSG00000256028	-1.647000489	-1.155892055	0.006300279	0.03819452	RP11-197N18.2	processed_transcript	5618
ENSG00000163659	-1.648694318	4.731451915	3.04E-13	2.66E-11	TIPARP	protein_coding	2700
ENSG00000121454	-1.654099064	-0.479590256	0.008107651	0.045812097	LHX4	protein_coding	721
ENSG00000234432	-1.654151427	-0.610544078	1.04E-05	0.000199016	RP11-1275H24.1	lincRNA	2651
ENSG00000205790	-1.656368258	-1.386068601	0.00521015	0.033076947	DPP9-AS1	antisense	3071
ENSG00000111981	-1.657026566	4.030873379	4.94E-11	3.02E-09	ULBP1	protein_coding	3142
ENSG00000272975	-1.660471946	-0.10853249	0.006281708	0.03811541	MYHAS	antisense	2274
ENSG00000167536	-1.660927682	-0.634790434	0.002087518	0.016233729	DHRS13	protein_coding	1569
ENSG00000110799	-1.661023401	-0.773974697	0.000571916	0.005732725	VWF	protein_coding	688
ENSG00000102760	-1.661380978	1.592660788	4.01E-06	8.66E-05	RGCC	protein_coding	601.5
ENSG00000260083	-1.665372292	-0.793849072	0.000117917	0.001589809	MIR762HG	antisense	1125
ENSG00000235530	-1.667212014	-1.784650907	0.006440235	0.038862407	AC087294.2	antisense	819.5
ENSG00000230513	-1.667234878	0.433313688	5.97E-08	2.05E-06	THAP7-AS1	antisense	1720
ENSG00000127863	-1.670281995	2.592618119	2.85E-11	1.80E-09	TNFRSF19	protein_coding	3276
ENSG00000164236	-1.672389214	3.928901924	1.44E-20	2.93E-18	ANKRD33B	protein_coding	5389.5
ENSG00000149809	-1.673809797	0.06154488	0.000129665	0.001715802	TM7SF2	protein_coding	792.5
ENSG00000272277	-1.67445761	-0.993386509	0.000124999	0.001665771	RP1-40E16.12	antisense	850
ENSG00000257135	-1.675143749	-0.269679593	6.98E-05	0.001022624	RP11-320M2.1	lincRNA	1234.5
ENSG00000139289	-1.679330699	4.948815835	1.83E-42	2.19E-39	PHLDA1	protein_coding	5913
ENSG00000246731	-1.680308593	-1.427567893	0.002907769	0.020996162	MGC16275	antisense	588
ENSG00000226200	-1.681234146	0.702495788	8.47E-05	0.001204598	SGMS1-AS1	antisense	6664.5
ENSG00000263843	-1.684755827	-1.636073775	0.006780417	0.040296932	RP11-649A18.12	antisense	1536.5
ENSG00000213693	-1.685884803	-0.215001362	2.50E-06	5.81E-05	SEC14L1P1	processed_pseudogene	2181
ENSG00000279382	-1.686064084	0.716263016	0.006656341	0.039816915	RP11-449J21.3	TEC	1362
ENSG00000108984	-1.687629128	2.005398502	1.96E-06	4.66E-05	MAP2K6	protein_coding	1512
ENSG00000278500	-1.688165634	-0.491608385	0.004863941	0.031310959	AC009336.19	protein_coding	3521
ENSG00000274292	-1.69394501	-0.445948998	0.007860473	0.044955133	RP11-347I19.7	lincRNA	2607
ENSG00000260260	-1.700136957	2.013974365	3.45E-15	4.11E-13	SNHG19	lincRNA	342
ENSG00000253210	-1.701366586	-1.057245148	0.005826297	0.035952559	RP11-809O17.1	antisense	494
ENSG00000133392	-1.703734828	-1.124921884	0.005453134	0.03423127	MYH11	protein_coding	4087
ENSG00000166793	-1.716315432	0.035774007	0.004521653	0.029585756	YPEL4	protein_coding	1263.5
ENSG00000273888	-1.720328719	0.139588855	4.02E-08	1.42E-06	FRMD6-AS1	antisense	2229
ENSG00000118407	-1.722381842	-1.333967348	0.008377316	0.04697694	FILIP1	protein_coding	4315.5
ENSG00000078814	-1.72805559	-0.447512366	0.00249604	0.018647365	MYH7B	protein_coding	699
ENSG00000213904	-1.729755615	-1.149510125	0.000637217	0.006235774	LIPE-AS1	antisense	747.5
ENSG00000131242	-1.740988799	-0.415223441	1.67E-05	0.000299195	RAB11FIP4	protein_coding	581.5
ENSG00000100784	-1.744675046	0.205245198	1.85E-05	0.000325422	RPS6KA5	protein_coding	2118
ENSG00000081059	-1.750174257	3.317150811	7.48E-14	7.10E-12	TCF7	protein_coding	782.5
ENSG00000261123	-1.750256554	-0.495568587	0.004191733	0.027903121	RP11-304L19.3	sense_intronic	596

ENSG00000274220	-1.7511525	-1.199779342	0.008100788	0.045798311	RP11-77K12.9	lincRNA	2557
ENSG00000265190	-1.752496057	-1.109851774	0.005477818	0.034323793	ANXA8	protein_coding	1873
ENSG00000101096	-1.753881823	0.670833149	4.73E-09	2.10E-07	NFATC2	protein_coding	2974
ENSG00000223820	-1.756093723	-1.586024179	0.008867935	0.048907073	CFL1P1	transcribed_unprocessed_pseudogene	447
ENSG00000163739	-1.760097787	1.126650306	1.65E-14	1.77E-12	CXCL1	protein_coding	835.5
ENSG00000279631	-1.770554635	0.047828367	9.10E-07	2.35E-05	RP11-573G6.4	TEC	2518
ENSG00000114270	-1.771359306	5.221900835	4.15E-27	1.56E-24	COL7A1	protein_coding	709
ENSG00000213213	-1.783019201	-0.715058051	0.004900875	0.031470457	CCDC183	protein_coding	1496.5
ENSG00000278611	-1.787127531	-1.514321727	0.002662675	0.019609293	CTC-543D15.8	lincRNA	727
ENSG00000259370	-1.788920451	0.395883291	5.96E-08	2.05E-06	RP11-1069G10.1	antisense	556
ENSG00000259523	-1.791869374	-0.524002828	4.92E-06	0.000104261	RP11-680F8.3	antisense	1451
ENSG00000280161	-1.793289245	-1.59222865	0.001888571	0.015013594	CTC-205M6.1	TEC	984
ENSG00000163734	-1.805178009	-1.07982245	0.000913924	0.008453085	CXCL3	protein_coding	929.5
ENSG00000188710	-1.80572842	-1.418751636	0.006503311	0.039116194	QRFP	protein_coding	1034.5
ENSG00000100292	-1.806562895	4.39847692	2.80E-46	4.14E-43	HMOX1	protein_coding	751.5
ENSG00000270419	-1.807022471	-1.552134965	0.000801013	0.007567597	CAHM	lincRNA	896
ENSG00000276791	-1.807727387	-1.101692677	7.80E-05	0.001124691	CTD-2270P14.5	lincRNA	3250
ENSG00000085741	-1.809527005	-0.024950064	4.06E-07	1.14E-05	WNT11	protein_coding	625
ENSG00000266947	-1.812384192	-1.256046306	0.000776147	0.007366283	RP11-799D4.4	antisense	1595
ENSG00000203546	-1.81285655	-0.853532926	1.77E-05	0.00031428	RP11-176H8.1	protein_coding	749
ENSG00000117394	-1.818065496	5.306851705	1.02E-25	3.30E-23	SLC2A1	protein_coding	591
ENSG00000265972	-1.820636755	7.769738291	6.43E-13	5.37E-11	TXNIP	protein_coding	1367
ENSG00000100036	-1.821994167	3.365403058	2.07E-09	9.86E-08	SLC35E4	protein_coding	2081
ENSG00000198113	-1.823168226	1.059814487	2.87E-10	1.57E-08	TOR4A	protein_coding	4148
ENSG00000146966	-1.824366961	2.940593705	1.06E-20	2.18E-18	DENND2A	protein_coding	2081
ENSG00000230359	-1.828421308	-0.44173467	2.92E-05	0.000482989	TPI1P2	transcribed_processed_pseudogene	1384.5
ENSG00000128285	-1.836111928	-1.592595888	0.003680762	0.025107013	MCHR1	protein_coding	1243
ENSG00000228536	-1.83681444	-1.089461154	0.007987501	0.045480662	RP11-392O17.1	lincRNA	811
ENSG00000196747	-1.839542329	-1.944850434	0.005306453	0.033544362	HIST1H2AI	protein_coding	503
ENSG00000265321	-1.841420622	-1.175339309	0.001319982	0.011351959	MIR4263	miRNA	83
ENSG00000157111	-1.846796258	1.670965223	1.85E-14	1.96E-12	TMEM171	protein_coding	1391
ENSG00000272143	-1.848687937	-1.528836157	0.003937131	0.026437761	FGF14-AS2	lincRNA	1074
ENSG00000246898	-1.855783991	-1.00025278	6.50E-05	0.00096067	LINC00920	lincRNA	2147
ENSG00000063438	-1.856264469	3.774350421	5.46E-18	8.49E-16	AHRR	protein_coding	646
ENSG00000151062	-1.860453515	-0.802586564	0.000415455	0.004448633	CACNA2D4	protein_coding	923.5
ENSG00000260588	-1.863577875	-0.538149326	5.48E-06	0.000114425	RP11-930P14.2	lincRNA	1770
ENSG00000271576	-1.863839153	-0.936823047	0.000150925	0.001940109	RP11-486G15.2	lincRNA	1601
ENSG00000068615	-1.874777414	-2.262589628	0.007235593	0.042185219	REEP1	protein_coding	958
ENSG00000165474	-1.875166172	0.466977824	4.50E-08	1.58E-06	GJB2	protein_coding	2279.5
ENSG00000158423	-1.875742939	-1.291090145	0.005384354	0.033995299	RIBC1	protein_coding	1281
ENSG00000236871	-1.875822147	0.202713096	0.008528134	0.04755227	LINC00106	lincRNA	363
ENSG00000166592	-1.877609699	0.902400906	3.28E-13	2.86E-11	RRAD	protein_coding	681
ENSG00000254639	-1.883323129	-1.420441163	5.16E-05	0.000794934	CTD-2589M5.5	lincRNA	653
ENSG00000123612	-1.883328962	-0.891720557	0.000465646	0.004870234	ACVR1C	protein_coding	1643.5
ENSG00000126860	-1.890556252	3.361804604	1.53E-07	4.80E-06	EVI2A	protein_coding	1854
ENSG00000101265	-1.893267222	4.060894298	7.35E-06	0.000147428	RASSF2	protein_coding	5282
ENSG00000184524	-1.897334453	-0.528139731	5.88E-07	1.58E-05	CEND1	protein_coding	1060.5
ENSG00000124116	-1.898801436	-1.364236498	0.000992972	0.009019059	WFDC3	protein_coding	704
ENSG00000269313	-1.900815694	-0.641615675	6.73E-06	0.000137691	MAGIX	protein_coding	1057
ENSG00000272702	-1.902757465	-1.158211917	0.000140372	0.001833056	RP11-44N22.3	processed_transcript	1582
ENSG00000216775	-1.903005957	2.478356033	5.76E-09	2.50E-07	RP1-152L7.5	transcribed_unprocessed_pseudogene	1737.5
ENSG00000224165	-1.904903296	-1.215559506	0.000871416	0.008103351	DNAJC27-AS1	antisense	488
ENSG00000173531	-1.906353662	0.892215872	0.000751085	0.007171151	MST1	protein_coding	859
ENSG00000240875	-1.906695655	0.710157714	7.97E-09	3.35E-07	LINC00886	lincRNA	601
ENSG00000100889	-1.91062696	5.693134962	1.17E-14	1.29E-12	PCK2	protein_coding	1350.5
ENSG00000189120	-1.914815532	-1.209169036	3.46E-05	0.000563378	SP6	protein_coding	3824
ENSG00000149922	-1.915759453	-0.783962213	0.00013524	0.001780471	TBX6	protein_coding	1796
ENSG00000136826	-1.916183138	5.321462382	5.97E-15	6.95E-13	KLF4	protein_coding	2174.5
ENSG00000279491	-1.919237697	-1.753876319	0.003294769	0.023040025	RP11-810P12.7	TEC	1697
ENSG00000228463	-1.921072736	-0.890574237	0.000266404	0.00309594	AP006222.2	lincRNA	1292
ENSG00000101255	-1.933248367	6.104510031	6.17E-15	7.10E-13	TRIB3	protein_coding	1301.5
ENSG00000235706	-1.934427123	-0.933798723	0.001279295	0.011071011	DICER1-AS1	antisense	1259.5
ENSG00000103257	-1.935810653	7.373804141	6.90E-22	1.62E-19	SLC7A5	protein_coding	3983
ENSG00000221883	-1.953003107	-0.239485755	3.54E-06	7.77E-05	ARIH2OS	protein_coding	1598
ENSG00000205041	-1.957315364	-1.57850839	0.007268712	0.042342547	CTC-425O23.2	sense_intronic	1462
ENSG00000102554	-1.958698699	2.619422187	1.00E-14	1.11E-12	KLF5	protein_coding	984
ENSG00000149506	-1.962155067	-1.498305588	0.001842109	0.014734736	ZP1	protein_coding	536
ENSG00000263412	-1.971954825	-1.645517821	0.003036603	0.021684488	RP5-890E16.2	processed_transcript	1724.5
ENSG00000246477	-1.982539694	-1.614659934	0.001701427	0.013844739	AF131216.6	antisense	5856
ENSG00000137868	-1.98461069	0.672430634	8.75E-14	8.09E-12	STRA6	protein_coding	2237.5
ENSG00000180044	-1.986485722	2.147286644	8.46E-09	3.52E-07	C3orf80	protein_coding	2372
ENSG00000141574	-1.988372841	1.687344809	8.45E-07	2.19E-05	SECTM1	protein_coding	712
ENSG00000118094	-1.991841302	-1.34429476	0.000537188	0.005455921	TREH	protein_coding	1718
ENSG00000227268	-1.994102633	0.630062052	2.71E-11	1.72E-09	KLLN	protein_coding	4277
ENSG00000235513	-1.994183594	1.534278906	1.01E-06	2.58E-05	RP4-756G23.5	antisense	1640
ENSG00000135605	-1.997639508	-0.276245716	1.04E-05	0.0001997	TEC	protein_coding	814
ENSG00000236404	-2.000055905	-0.170268595	0.001209651	0.010552092	VLDLR-AS1	antisense	887
ENSG00000270177	-2.00518118	-1.689833928	0.001041471	0.009344813	CTD-2410N18.3	lincRNA	1418
ENSG00000154928	-2.006303289	0.15735287	3.06E-09	1.41E-07	EPHB1	protein_coding	566.5
ENSG00000272223	-2.007539038	-1.947347479	0.006764254	0.040241578	RP1-20C7.6	antisense	509

ENSG00000260285	-2.008267791	-0.738683796	1.16E-05	0.000218319	RP11-600F24.7	antisense	4063
ENSG00000231663	-2.008377883	-1.368418609	0.000859408	0.008025501	RP5-827C21.4	antisense	575
ENSG00000268218	-2.011396752	-1.076482433	0.007833631	0.044837895	AC137932.4	antisense	3660
ENSG00000272030	-2.020530166	-0.862409241	0.000309302	0.00351555	RP1-178F15.4	antisense	691.5
ENSG00000092068	-2.028315323	2.920721017	3.26E-09	1.49E-07	SLC7A8	protein_coding	1625.5
ENSG00000258130	-2.03274395	-1.29165799	0.002897794	0.020931433	RP11-347C12.3	protein_coding	569
ENSG00000125864	-2.032881779	-1.261796063	0.001744317	0.014110569	BFSP1	protein_coding	1384
ENSG00000141294	-2.046782001	-1.228502334	0.00091387	0.008453085	LRRC46	protein_coding	872
ENSG00000262251	-2.050713067	-1.692626487	0.000327973	0.003673075	RP11-199F11.2	sense_intronic	1112
ENSG00000243819	-2.058502767	-1.452705998	0.001740527	0.014096431	RN7SL832P	lincRNA	1756
ENSG00000277287	-2.063440133	-0.883901811	0.000535064	0.00543702	RP4-794I6.4	lincRNA	5678
ENSG00000204396	-2.066295631	-1.418946044	0.003147328	0.022298141	VWA7	protein_coding	853
ENSG00000231784	-2.069929498	-1.130605333	2.94E-06	6.68E-05	DBIL5P	transcribed_unitary_pseudogene	672
ENSG00000124440	-2.073071093	-0.138967079	0.001116776	0.009879241	HIF3A	protein_coding	1031
ENSG00000168517	-2.077143564	0.635715661	1.88E-06	4.49E-05	HEXIM2	protein_coding	799.5
ENSG00000243056	-2.079368112	-0.383781967	0.00012608	0.001678012	EIF4EBP3	protein_coding	691
ENSG00000224934	-2.07957242	-0.988218589	0.003513536	0.024229709	RP11-441O15.3	lincRNA	2998
ENSG00000267416	-2.080835693	-1.706714638	0.007035687	0.041380871	CTD-2319I12.2	lincRNA	699
ENSG00000260274	-2.085172092	-1.044869644	0.000271761	0.003149354	RP11-817O13.8	lincRNA	1430
ENSG00000171368	-2.085568968	-1.952734561	0.000582685	0.005813806	TPPP	protein_coding	6022
ENSG00000010327	-2.088864906	-1.113218339	0.000933361	0.008598302	STAB1	protein_coding	2160
ENSG00000188385	-2.091412706	-0.88954755	0.003228512	0.022694924	JAKMIP3	protein_coding	6033
ENSG00000145911	-2.09527979	-0.857265086	1.50E-05	0.000271302	N4BP3	protein_coding	6080
ENSG00000064687	-2.098121243	1.796560082	3.84E-08	1.36E-06	ABCA7	protein_coding	591
ENSG00000257390	-2.103823533	-1.397607003	0.000259464	0.0030323	RP11-762I7.5	protein_coding	782
ENSG00000273084	-2.10630552	-1.199397727	5.03E-05	0.000779092	RP11-1275H24.3	lincRNA	942
ENSG00000225950	-2.119360675	-1.944942915	0.009101038	0.049939954	NTF4	protein_coding	813
ENSG00000167476	-2.12286087	-1.285239202	0.004976169	0.031845326	JSRP1	protein_coding	843
ENSG00000277449	-2.12729305	-1.307850689	0.000393749	0.004255823	CEBPB-AS1	antisense	2648
ENSG00000253948	-2.155227942	-0.878049007	1.05E-06	2.67E-05	RP11-410L14.2	lincRNA	380
ENSG00000111087	-2.155314672	3.005776306	1.56E-09	7.58E-08	GLI1	protein_coding	1990
ENSG00000166924	-2.156015219	-1.607830145	0.005627308	0.0350188	NYAP1	protein_coding	1786.5
ENSG00000175264	-2.160977904	-0.385541084	5.64E-07	1.53E-05	CHST1	protein_coding	1361
ENSG00000267013	-2.163746876	0.929928404	1.33E-13	1.21E-11	CTD-2171N6.1	lincRNA	1821.5
ENSG00000138061	-2.165354224	6.733458408	2.46E-33	1.76E-30	CYP1B1	protein_coding	745
ENSG00000255153	-2.173658056	-1.13972885	3.81E-05	0.000612353	TOLLIP-AS1	antisense	939
ENSG00000154856	-2.174954206	1.836868214	1.20E-11	8.07E-10	APCDD1	protein_coding	788.5
ENSG00000267194	-2.179464106	-0.454175816	0.000482396	0.005004975	RP1-193H18.2	lincRNA	2504
ENSG00000049249	-2.183198554	1.779023757	1.53E-09	7.47E-08	TNFRSF9	protein_coding	1253.5
ENSG00000188042	-2.187705817	5.695030507	6.76E-66	2.33E-62	ARL4C	protein_coding	3699
ENSG00000184792	-2.192852268	1.972052324	7.51E-10	3.86E-08	OSBP2	protein_coding	2468
ENSG00000160111	-2.195998151	-1.44551011	0.008607824	0.047842036	CPAMD8	protein_coding	643.5
ENSG00000185065	-2.198565268	-2.301665035	0.00644684	0.038878163	AC000068.5	antisense	2097
ENSG00000138316	-2.201213482	4.213303569	2.33E-16	3.18E-14	ADAMTS14	protein_coding	5264.5
ENSG00000256802	-2.203941041	-0.792771634	6.61E-08	2.25E-06	RP11-680F8.1	antisense	1470
ENSG00000228363	-2.206045857	-1.711530567	0.005148155	0.032735609	AC015971.2	antisense	982.5
ENSG00000273062	-2.209533643	-1.868455218	0.0048512	0.03125809	RP11-428K3.1	antisense	1980
ENSG00000122877	-2.210283292	4.047992521	2.12E-28	9.73E-26	EGR2	protein_coding	2824
ENSG00000133863	-2.213768332	-2.225178066	0.002628825	0.019415241	TEX15	protein_coding	1520
ENSG00000102981	-2.213823733	-1.491787132	0.002053298	0.016033842	PARD6A	protein_coding	1254
ENSG00000164161	-2.216004867	-0.883136551	2.27E-05	0.000387598	HHIP	protein_coding	704.5
ENSG00000273619	-2.218257901	-0.430909954	3.10E-07	9.08E-06	RP5-908M14.9	antisense	668
ENSG00000259230	-2.21903715	-2.147901651	0.001723081	0.01397153	CTD-2555C10.3	lincRNA	1603
ENSG00000153094	-2.220668024	2.87469973	1.69E-14	1.81E-12	BCL2L11	protein_coding	936
ENSG00000225138	-2.221181382	1.848727289	2.76E-05	0.000460067	CTD-2228K2.7	processed_transcript	859
ENSG00000271855	-2.226424613	-1.932947998	0.001656353	0.013515135	RP11-214N9.1	lincRNA	877
ENSG00000130783	-2.234317841	-1.666623873	0.004682785	0.030419008	CCDC62	protein_coding	2707.5
ENSG00000273783	-2.23537757	-1.370449115	3.89E-05	0.000624287	CTD-2506P8.6	antisense	1130
ENSG00000144230	-2.244586591	-1.734119106	0.002406363	0.01808834	GPR17	protein_coding	1651.5
ENSG00000162526	-2.245239675	-1.63044811	0.001035986	0.009299624	TSSK3	protein_coding	494
ENSG00000160781	-2.255628002	-1.380929026	0.005686497	0.035225033	PAQR6	protein_coding	1799
ENSG00000261485	-2.255848944	-0.90884163	5.29E-07	1.45E-05	PAN3-AS1	antisense	1351
ENSG00000265485	-2.256781244	-0.890483149	0.001224656	0.010669503	RP11-449D8.1	lincRNA	1458.5
ENSG00000152154	-2.26207573	-1.598227981	0.008435197	0.047173775	TMEM178A	protein_coding	703.5
ENSG00000204055	-2.265674924	-1.595696977	0.000135993	0.001788193	RP11-247A12.2	antisense	1303
ENSG00000243024	-2.265972379	-1.601290649	0.00044724	0.00471823	RPS11P6	transcribed_processed_pseudogene	1242
ENSG00000111644	-2.266344636	-1.857743946	0.007178564	0.041953984	ACRBP	protein_coding	811
ENSG00000272463	-2.269682575	-1.978708264	0.000772634	0.007343039	RP11-532F6.3	lincRNA	2814
ENSG00000229980	-2.273617301	-1.493961636	0.001001882	0.009080077	TOB1-AS1	processed_transcript	618.5
ENSG00000108551	-2.277304478	-0.90769595	7.09E-05	0.001036682	RASD1	protein_coding	1575
ENSG00000101188	-2.277928398	1.790185482	9.89E-09	4.04E-07	NTSR1	protein_coding	3541
ENSG00000228626	-2.279426474	-0.868844296	0.004996125	0.031913869	RP11-495P10.9	unprocessed_pseudogene	285
ENSG00000154319	-2.282909861	1.749586497	7.58E-22	1.74E-19	FAM167A	protein_coding	1325
ENSG00000196468	-2.290390777	-1.121301077	6.78E-06	0.00013843	FGF16	protein_coding	1650
ENSG00000272986	-2.290947944	-2.1648361	0.001732941	0.014040481	RP11-46J23.1	antisense	745
ENSG00000162620	-2.297702837	-1.993753335	0.004585039	0.029896592	LRRIQ3	protein_coding	1338
ENSG00000130518	-2.299631644	-0.598682089	0.001616269	0.013234996	KIAA1683	protein_coding	2684
ENSG00000275964	-2.329146953	0.382328208	4.13E-13	3.52E-11	RP11-61K9.3	lincRNA	1191
ENSG00000185112	-2.331046731	1.880497858	2.27E-15	2.75E-13	FAM43A	protein_coding	2494
ENSG00000129757	-2.33242889	2.136081449	2.75E-08	1.00E-06	CDKN1C	protein_coding	1570

ENSG00000273802	-2.335918117	-1.067867687	1.75E-07	5.39E-06	HIST1H2BG	protein_coding	1534
ENSG00000275198	-2.339087408	-1.245904889	5.51E-05	0.000835916	RP11-471B22.3	lincRNA	4609
ENSG00000155980	-2.339441244	-0.882771427	8.27E-05	0.001182121	KIF5A	protein_coding	3527
ENSG00000186301	-2.34873077	-0.400448066	0.003657186	0.024970879	MST1P2	unprocessed_pseudogene	2291
ENSG00000272172	-2.350943476	-1.947395254	0.006508862	0.039126859	RP13-582O9.7	antisense	223
ENSG00000277383	-2.351801666	-1.968314035	0.003208928	0.022587932	CTD-3001H11.2	antisense	582
ENSG00000251417	-2.353080674	-1.780139831	0.00298796	0.021448066	RP11-1348G14.4	lincRNA	1963
ENSG00000277715	-2.358931423	-1.579800979	0.001968767	0.015543729	RP11-651L5.3	antisense	2028
ENSG00000182366	-2.367559388	-1.174609417	1.58E-06	3.84E-05	FAM87A	lincRNA	1221
ENSG00000264107	-2.368845478	-1.210622594	0.001111682	0.009846806	RP11-848P1.5	antisense	492
ENSG00000174564	-2.374293287	2.226943417	2.58E-07	7.68E-06	IL20RB	protein_coding	1787
ENSG00000229867	-2.375194586	-1.465176917	1.88E-05	0.000330572	STEAP3-AS1	antisense	3218
ENSG00000185338	-2.379750991	1.418924365	9.27E-13	7.44E-11	SOCS1	protein_coding	1225
ENSG00000125378	-2.379877074	-1.083812076	0.000249882	0.002931668	BMP4	protein_coding	1112
ENSG00000223473	-2.38349358	-1.30548062	0.002132198	0.016506798	GS1-124K5.3	lincRNA	714
ENSG00000172508	-2.385848933	-1.874137781	0.003861982	0.026068376	CARNS1	protein_coding	2968
ENSG00000123329	-2.39324617	-1.294327202	0.002474158	0.018544094	ARHGAP9	protein_coding	673
ENSG00000270605	-2.39943242	-1.124929408	0.001427909	0.012055087	RP5-1092A3.4	antisense	1945
ENSG00000198417	-2.401058531	-1.297976213	0.001120581	0.009904447	MT1F	protein_coding	553
ENSG00000167992	-2.402200708	0.91440438	1.12E-05	0.000211761	VWCE	protein_coding	1256.5
ENSG00000168062	-2.403679568	-2.166665348	0.004595829	0.029948089	BATF2	protein_coding	1949
ENSG00000274213	-2.405684816	-1.55319514	9.93E-05	0.001374146	RP11-670E13.6	lincRNA	348
ENSG00000247157	-2.412700249	-1.756052874	0.008320058	0.046744462	LINC01252	lincRNA	502
ENSG00000225335	-2.419247912	-1.861442677	0.001854185	0.014814164	XXbac-B476C20.9	antisense	805
ENSG00000165197	-2.4236825	0.474650134	2.58E-06	5.97E-05	VEGFD	protein_coding	1318.5
ENSG00000251257	-2.426201022	-2.079265685	0.000841118	0.007888906	CTD-2263F21.1	antisense	397.5
ENSG00000130513	-2.428469186	3.708152361	1.84E-10	1.03E-08	GDF15	protein_coding	562
ENSG00000178977	-2.43554721	-1.725849506	0.004416113	0.029027987	LINC00324	lincRNA	2082
ENSG00000100027	-2.441486722	-1.770047981	0.0020694	0.016132511	YPEL1	protein_coding	1319
ENSG00000272734	-2.455303318	1.782392406	8.15E-07	2.12E-05	ADIRF-AS1	processed_transcript	2416
ENSG00000260088	-2.466686223	-2.297527403	0.007533702	0.043470448	RP11-92G12.3	lincRNA	736
ENSG00000262075	-2.468251324	-2.088782857	0.007730128	0.044356669	DKFZP434A062	lincRNA	3968.5
ENSG00000163884	-2.471231771	-1.45428329	0.004745661	0.030759905	KLF15	protein_coding	1560.5
ENSG00000198208	-2.474339677	-1.221979968	5.64E-05	0.000853825	RPS6KL1	protein_coding	1436.5
ENSG00000251127	-2.48798228	-2.42749385	0.008036025	0.045606479	RP11-280G9.1	lincRNA	2750
ENSG00000230454	-2.493063493	-1.045898353	0.001094585	0.009728651	U73166.2	lincRNA	430
ENSG00000283078	-2.493770591	-1.98007971	0.002794497	0.020327094	RP11-11M20.4	lincRNA	1608
ENSG00000232739	-2.495232797	-2.338507593	0.007571918	0.04364232	RP11-25G10.2	antisense	794
ENSG00000066230	-2.503964949	-0.677737947	0.001682986	0.013721636	SLC9A3	protein_coding	2504
ENSG00000185271	-2.504250956	-1.085514893	9.77E-06	0.000188676	KLHL33	protein_coding	2394
ENSG00000255201	-2.517057118	-0.950313888	0.002330755	0.017686921	RP11-350N15.4	antisense	534
ENSG00000256508	-2.517675977	-1.478556658	0.003977011	0.026688246	MRGPRF-AS1	antisense	542
ENSG00000258708	-2.518796849	-1.742840141	0.000375407	0.00409821	SLC25A21-AS1	antisense	1924
ENSG00000227953	-2.523595104	-1.814314759	0.002504323	0.018689019	LINC01341	processed_transcript	595
ENSG00000171094	-2.535450335	-0.72390747	8.32E-10	4.24E-08	ALK	protein_coding	2190
ENSG00000117425	-2.539998576	0.752791037	7.99E-06	0.000157472	PTCH2	protein_coding	4225
ENSG00000254607	-2.544155965	-2.076259394	0.001784801	0.014398097	RP11-115C10.1	antisense	403.5
ENSG00000267546	-2.5448325	-2.131957146	0.008158687	0.04603767	RP11-666A8.8	antisense	554
ENSG00000145777	-2.548953773	-0.115146839	6.46E-09	2.78E-07	TSLP	protein_coding	2411
ENSG00000225756	-2.551582684	-0.942216771	0.00026411	0.003072731	DBH-AS1	antisense	2139
ENSG00000196220	-2.55761635	2.242397646	1.94E-06	4.62E-05	SRGAP3	protein_coding	1799
ENSG00000237940	-2.562497393	-0.906665028	0.002305202	0.017544445	AC093642.3	lincRNA	594.5
ENSG00000125538	-2.565068426	-1.049901179	0.000123496	0.001648917	IL1B	protein_coding	617.5
ENSG00000137825	-2.570615869	-0.412918973	6.85E-09	2.91E-07	ITPKA	protein_coding	853
ENSG00000167178	-2.572879489	1.393217949	1.44E-19	2.67E-17	ISLR2	protein_coding	1471
ENSG00000214796	-2.575052548	-2.132423474	0.00145268	0.012209236	RP11-480I12.5	transcribed_unprocessed_pseudogene	961
ENSG00000177173	-2.584295307	-2.168272352	0.003010569	0.021559843	NAP1L4P1	processed_pseudogene	1157
ENSG00000123689	-2.58478551	3.101656667	1.21E-10	6.95E-09	G0S2	protein_coding	866
ENSG00000244040	-2.589656846	-1.897871209	0.000865024	0.008058383	IL12A-AS1	antisense	988
ENSG00000171658	-2.603227014	-0.452283853	0.001403511	0.011926673	NMRAL1P1	transcribed_unprocessed_pseudogene	574
ENSG00000229160	-2.603358729	-1.966861393	0.005449262	0.034217335	AC009229.6	lincRNA	351
ENSG00000181790	-2.612256178	-1.575415841	0.000505542	0.005190511	ADGRB1	protein_coding	5527
ENSG00000137841	-2.617471831	-1.574043223	0.000867217	0.008071552	PLCB2	protein_coding	563
ENSG00000257108	-2.646655979	-1.369410552	3.99E-05	0.000637086	NHLRC4	protein_coding	2084
ENSG00000262155	-2.646726504	-2.318411105	0.001702783	0.013850327	RP11-266L9.5	lincRNA	1875
ENSG00000183778	-2.65242642	-2.539310953	0.00221591	0.017008629	B3GALT5	protein_coding	2611.5
ENSG00000214814	-2.655937845	0.330572334	1.62E-05	0.000290684	FER1L6	protein_coding	6051
ENSG00000225077	-2.65905578	-1.966817715	0.000377093	0.004112284	LINC00337	lincRNA	1220
ENSG00000229774	-2.704694568	-2.767578345	0.007636039	0.043926313	AC018866.1	lincRNA	397
ENSG00000233559	-2.712584209	-2.165977765	0.007224808	0.04214607	AC016831.7	lincRNA	1616
ENSG00000250539	-2.712630898	-1.963065182	0.003274383	0.022947272	KRT8P33	processed_pseudogene	1423
ENSG00000123700	-2.722482852	-0.813186748	2.60E-06	5.98E-05	KCNJ2	protein_coding	3538.5
ENSG00000280334	-2.723399316	-1.541831952	0.003708646	0.02524732	RP11-361L15.5	TEC	2810
ENSG00000242852	-2.723757084	-2.515605422	0.003072581	0.021899844	ZNF709	protein_coding	798
ENSG00000004799	-2.731788376	1.424635166	7.89E-11	4.67E-09	PDK4	protein_coding	691
ENSG00000272525	-2.75248447	-2.224051256	0.000517018	0.005292601	RP11-79P5.9	lincRNA	744
ENSG00000270019	-2.769221079	-2.242418343	0.00909721	0.049932179	RP11-141B14.1	lincRNA	1548
ENSG00000188649	-2.771533431	-1.729166685	0.000431944	0.004582547	CC2D2B	protein_coding	1428
ENSG00000232615	-2.775613617	-2.361473435	0.002152473	0.016638729	CTD-2012J19.1	processed_pseudogene	1075
ENSG00000252498	-2.80313615	-2.117442565	0.008083049	0.045749972	RNU6-1016P	snRNA	94

ENSG00000233695	-2.81829075	1.463966938	2.32E-08	8.65E-07	GAS6-AS1	antisense	4493
ENSG00000236969	-2.839652045	-2.301940709	0.002569847	0.01909543	GGT8P	unprocessed_pseudogene	532
ENSG00000175197	-2.855984339	4.234794732	3.07E-09	1.41E-07	DDIT3	protein_coding	928.5
ENSG00000269194	-2.867131113	-2.127084272	0.00030246	0.003447244	AC006942.4	antisense	520
ENSG00000164746	-2.870701502	-2.404230229	0.002037565	0.015941042	C7orf57	protein_coding	1942
ENSG00000279283	-2.878946795	-2.399737692	0.007965528	0.04539299	RP11-417L19.5	TEC	1740
ENSG00000231317	-2.881214063	-2.278444687	0.008296437	0.04664972	RP11-310H4.6	unprocessed_pseudogene	1948
ENSG00000130487	-2.883335143	1.009185169	0.007386318	0.042870871	KLHDC7B	protein_coding	2991
ENSG00000189350	-2.886442489	-2.766121848	0.006781783	0.040296932	FAM179A	protein_coding	720
ENSG00000113739	-2.890763053	6.118994047	7.64E-13	6.28E-11	STC2	protein_coding	582
ENSG00000110076	-2.910281755	-0.55803568	2.12E-08	8.01E-07	NRXN2	protein_coding	853
ENSG00000233622	-2.921611797	-1.576884456	1.13E-05	0.000213476	CYP2T1P	unitary_pseudogene	1285
ENSG00000143994	-2.922489144	-2.364721373	0.001242482	0.010802079	ABHD1	protein_coding	1277
ENSG00000206026	-2.928655625	-1.585596903	6.19E-05	0.000920065	SMIM21	protein_coding	1834
ENSG00000229178	-2.928968563	-2.360192859	0.001263888	0.010965164	AC069513.4	lincRNA	1684
ENSG00000279205	-2.932428068	-2.468284825	0.000770762	0.007332368	RP11-632P5.1	TEC	1624
ENSG00000243224	-2.95040741	-1.265606672	2.43E-06	5.65E-05	RP5-1157M23.2	antisense	1078.5
ENSG00000246339	-2.967021432	-1.921923745	0.003339004	0.02329765	EXTL3-AS1	antisense	836.5
ENSG00000168209	-2.972541743	6.647051307	4.14E-14	4.14E-12	DDIT4	protein_coding	753
ENSG00000251079	-2.977111651	-1.54418104	0.000852914	0.007985081	BMS1P2	transcribed_unprocessed_pseudogene	1595.5
ENSG00000175874	-2.986151911	-1.509825877	1.42E-05	0.000257957	CREG2	protein_coding	906
ENSG00000222020	-2.988416814	-1.624518544	0.000335573	0.003750343	AC062017.1	antisense	414
ENSG00000137078	-2.989896285	-1.828945571	4.61E-06	9.86E-05	SIT1	protein_coding	1082
ENSG00000078795	-2.999636177	-2.636470158	0.003875289	0.026141162	PKD2L2	protein_coding	2185
ENSG00000223461	-3.004122456	-2.663036907	0.004279149	0.028339268	AC004471.9	antisense	582
ENSG00000162755	-3.015306789	-1.902903778	0.003128753	0.022197105	KLHDC9	protein_coding	848.5
ENSG00000197046	-3.032514309	0.489805958	4.19E-14	4.17E-12	SIGLEC15	protein_coding	1054
ENSG00000141431	-3.055277504	-1.430429747	4.59E-06	9.83E-05	ASXL3	protein_coding	565
ENSG00000156042	-3.060608306	-1.185001228	6.24E-05	0.000926411	CFAP70	protein_coding	1556.5
ENSG00000253616	-3.077414637	-2.135161386	0.000886374	0.008223976	RP11-875O11.3	antisense	467
ENSG00000230325	-3.079626311	-2.537986287	0.009056165	0.049794195	RP11-385F5.4	antisense	494
ENSG00000245468	-3.084681914	-2.466833869	0.007995343	0.045494205	RP11-367J11.3	lincRNA	1500
ENSG00000140090	-3.087911901	-2.662794735	0.007007744	0.041275132	SLC24A4	protein_coding	4302
ENSG00000250657	-3.122690595	-0.922910319	0.001178988	0.010340146	RP11-1E6.1	processed_transcript	551.5
ENSG00000267871	-3.130942454	-2.128138533	0.001044759	0.009366196	CTC-444N24.6	antisense	613.5
ENSG00000272808	-3.132720899	-2.061409661	0.001020517	0.009184655	RP11-66B24.7	processed_transcript	5068
ENSG00000185933	-3.14612498	-2.661255206	0.007892599	0.045101498	CALHM1	protein_coding	3053
ENSG00000243431	-3.146154042	-2.098284103	0.004335061	0.028572497	RPL5P30	processed_pseudogene	891
ENSG00000155961	-3.148033112	1.638348256	2.31E-08	8.62E-07	RAB39B	protein_coding	3505
ENSG00000223723	-3.164712323	-2.300269818	0.007352075	0.04272001	BX842568.2	processed_pseudogene	1230
ENSG00000227744	-3.173048918	-1.889006642	3.13E-05	0.000513261	FLJ43879	lincRNA	3489
ENSG00000169129	-3.186854528	-0.542362475	5.27E-07	1.44E-05	AFAP1L2	protein_coding	2332.5
ENSG00000187627	-3.192212722	-2.79893129	0.006928557	0.040971871	RGPD1	protein_coding	6095
ENSG00000263982	-3.193442422	-2.491460836	0.001862559	0.014863871	RP11-504I13.3	antisense	398
ENSG00000128165	-3.193740949	4.755620272	6.98E-08	2.36E-06	ADM2	protein_coding	2405.5
ENSG00000258902	-3.194976924	-2.491491695	0.003408932	0.023681839	CTD-2128A3.2	lincRNA	558
ENSG00000281912	-3.212030603	-1.967143493	0.003406772	0.023674777	LINC01144	lincRNA	1710
ENSG00000231105	-3.238386182	-2.467856986	0.006784469	0.040296932	RP5-1071N3.1	antisense	2479
ENSG00000267365	-3.246345394	-2.385422012	0.001828156	0.014662778	KCNJ2-AS1	antisense	2442
ENSG00000281026	-3.252058019	-1.900942759	0.000444569	0.004694844	N4BP2L2-IT2	sense_intronic	4890
ENSG00000163909	-3.254518802	-1.735852136	1.90E-05	0.000333353	HEYL	protein_coding	3661
ENSG00000230224	-3.26279606	-2.067586538	0.007906053	0.045141012	PHBP9	processed_pseudogene	825
ENSG00000166823	-3.269691684	-1.871124477	9.12E-05	0.001280992	MESP1	protein_coding	1410
ENSG00000236056	-3.279251021	-2.614444657	0.006271117	0.038093509	GAPDHP14	processed_pseudogene	937
ENSG00000199575	-3.319386973	-2.385568457	0.006202935	0.037759538	SNORD114-1	snoRNA	72
ENSG00000218052	-3.32586075	-0.72492782	9.65E-12	6.64E-10	ADAMTS7P4	transcribed_unprocessed_pseudogene	511.5
ENSG00000226091	-3.32635838	-2.562969873	0.007189934	0.042001789	LINC00937	lincRNA	624
ENSG00000118307	-3.328092242	-1.979970265	0.000630452	0.006184761	CASC1	protein_coding	1774
ENSG00000049283	-3.340738163	-1.803534575	0.000340252	0.003786286	EPN3	protein_coding	601
ENSG00000124882	-3.345102837	-0.134198229	2.17E-09	1.03E-07	EREG	protein_coding	1290
ENSG00000249755	-3.351778288	-2.284383818	0.002042609	0.015969606	RP11-466G12.2	processed_pseudogene	657
ENSG00000196666	-3.360478105	-2.051397136	0.002486762	0.018611623	FAM180B	protein_coding	1388
ENSG00000135577	-3.362771849	-2.636439106	0.002093735	0.016268832	NMBR	protein_coding	877.5
ENSG00000151812	-3.365434844	-1.837825793	0.00045858	0.004823122	SLC35F4	protein_coding	1071.5
ENSG00000280025	-3.370376746	-1.814742119	1.50E-05	0.000271302	CTA-992D9.8	lincRNA	4254
ENSG00000259644	-3.371621811	-2.769609271	0.002006621	0.015758481	RP11-680F8.4	sense_intronic	1346
ENSG00000267882	-3.381671526	-2.539370347	0.001132799	0.009987767	RP4-569M23.5	antisense	2253
ENSG00000103710	-3.386505558	-2.223852939	0.000163606	0.002076463	RASL12	protein_coding	1523
ENSG00000245667	-3.387351181	-2.471205334	0.005323611	0.033632291	RP5-940J5.8	antisense	560
ENSG00000182261	-3.403128095	-1.123754332	5.47E-05	0.000832517	NLRP10	protein_coding	1386.5
ENSG00000206344	-3.424243093	-2.172721618	0.005639238	0.035038396	HCG27	protein_coding	783
ENSG00000256443	-3.453869721	-2.099923787	0.006120944	0.037347857	RP11-794G24.1	lincRNA	1831
ENSG00000251287	-3.467693805	-2.25894794	0.000436351	0.0046222	ALG1L2	protein_coding	815
ENSG00000186715	-3.474085264	-2.146904341	0.004793435	0.031011325	MST1L	transcribed_unprocessed_pseudogene	2185
ENSG00000119608	-3.484650664	-2.152085225	0.006698121	0.039905461	PROX2	protein_coding	3359
ENSG00000172349	-3.485065158	1.415367358	1.28E-17	1.93E-15	IL16	protein_coding	2158
ENSG00000262481	-3.520282593	-1.957913105	0.000459525	0.004828163	TMEM256-PLSCR3	protein_coding	1238.5
ENSG00000274414	-3.553226967	-2.18314915	0.004528797	0.029623139	RP5-965G21.4	lincRNA	2335
ENSG00000227533	-3.560449958	-1.521130976	7.36E-06	0.000147552	SLC2A1-AS1	lincRNA	2099.5
ENSG00000113722	-3.563045428	-0.683071454	2.84E-08	1.03E-06	CDX1	protein_coding	1550.5

ENSG00000118898	-3.576846372	0.685217941	1.31E-06	3.26E-05	PPL	protein_coding	2256
ENSG00000280168	-3.583693527	-2.564937927	0.000522576	0.005332219	RP11-1090M7.3	TEC	802
ENSG00000242798	-3.633925389	-1.811631951	0.001457449	0.012231455	RP11-506M12.1	antisense	572
ENSG00000269901	-3.662413407	-2.261512039	0.000364575	0.004009547	RP11-178L8.9	antisense	450
ENSG00000276953	-3.698080722	-2.103018355	0.002028401	0.015899381	TRBV12-4	TR_V_gene	406
ENSG00000230415	-3.702254669	-1.693668389	0.000273239	0.003162944	RP5-902P8.10	lincRNA	328.5
ENSG00000233006	-3.766038682	-2.166741751	0.000947566	0.008678968	AC034220.3	processed_transcript	670
ENSG00000197769	-3.773644591	1.703386904	5.81E-13	4.87E-11	MAP1LC3C	protein_coding	1182
ENSG00000151012	-3.7976776	5.564998221	9.73E-15	1.09E-12	SLC7A11	protein_coding	5221
ENSG00000263818	-3.808328454	-1.954464445	0.001567326	0.012941684	CTD-2206N4.4	transcribed_processed_pseudogene	1046
ENSG00000157445	-3.909542367	-0.482250665	1.18E-14	1.29E-12	CACNA2D3	protein_coding	2634
ENSG00000241935	-3.950193924	-1.944802924	4.69E-05	0.000733921	HOGA1	protein_coding	2243.5
ENSG00000162641	-4.000718804	-2.187696128	0.000167877	0.002113413	AKNAD1	protein_coding	2638
ENSG00000249628	-4.023237161	-1.585338719	1.91E-05	0.000335239	LINC00942	lincRNA	2417
ENSG00000145242	-4.225795792	-2.32218569	1.77E-06	4.27E-05	EPHA5	protein_coding	6721
ENSG00000111728	-4.234881576	-1.127894981	1.73E-08	6.66E-07	ST8SIA1	protein_coding	572
ENSG00000141469	-4.240768598	0.316645441	2.91E-08	1.05E-06	SLC14A1	protein_coding	779
ENSG00000139269	-4.241445518	3.940201337	4.32E-09	1.94E-07	INHBE	protein_coding	1142
ENSG00000099960	-4.242059095	-0.330897037	1.44E-08	5.68E-07	SLC7A4	protein_coding	2307
ENSG00000130540	-4.265072336	-2.285287102	0.000154438	0.001976148	SULT4A1	protein_coding	2353.5
ENSG00000226744	-4.423630431	-0.751326263	5.12E-06	0.000107826	AC079781.5	processed_pseudogene	123
ENSG00000221947	-4.606438899	-2.711078766	0.008336283	0.046822913	XKR9	protein_coding	1799
ENSG00000223776	-4.623397507	-2.913590548	0.007944388	0.045309924	LGALS8-AS1	antisense	1443.5
ENSG00000164142	-4.624885247	-2.582765297	0.007527622	0.043452225	FAM160A1	protein_coding	567.5
ENSG00000238164	-4.632490643	-2.512730046	0.007028014	0.041370993	RP3-395M20.8	antisense	1218.5
ENSG00000267277	-4.639292201	-2.560333123	0.003911956	0.026337027	CTD-2342J14.6	antisense	682
ENSG00000263426	-4.657063424	-2.443989172	0.007483693	0.043278356	RN7SL471P	misc_RNA	299
ENSG00000189045	-4.748312596	-2.449318456	0.003603443	0.024652691	ANKDD1B	protein_coding	1303
ENSG00000279645	-4.753174161	-2.853939129	0.004300118	0.028450847	RP11-759A24.1	TEC	4198
ENSG00000149646	-4.754580263	-2.740126664	0.007453303	0.043138749	CNBD2	protein_coding	1722
ENSG00000188305	-4.773452228	-2.539445627	0.003450074	0.023911507	C19orf35	protein_coding	2574
ENSG00000253730	-4.776760532	-2.944096941	0.003896465	0.026258348	RP11-893F2.13	antisense	547
ENSG00000205334	-4.852166495	-2.740921932	0.00669467	0.039905461	LINC01460	lincRNA	2860
ENSG00000267193	-4.853175422	-2.945595465	0.006691528	0.039900622	RP11-116O18.3	antisense	430
ENSG00000281021	-4.880793982	-2.883692555	0.001724941	0.013981133	RP1-12G14.9	antisense	744
ENSG00000128965	-4.886141688	4.374374303	8.72E-11	5.08E-09	CHAC1	protein_coding	1498
ENSG00000099957	-4.891969234	-1.059977639	3.80E-09	1.71E-07	P2RX6	protein_coding	1834
ENSG00000099937	-4.895693766	-2.663176344	0.001304135	0.011253053	SERPIND1	protein_coding	2117.5
ENSG00000198658	-4.929194173	-2.851823525	0.003527421	0.02430474	ABHD17AP1	unprocessed_pseudogene	933
ENSG00000241684	-4.978302619	-2.254077227	0.003158743	0.022318119	ADAMTS9-AS2	antisense	2179
ENSG00000231443	-4.986670949	-2.56206518	0.005090281	0.032405358	AC024937.6	processed_pseudogene	1242
ENSG00000253125	-4.998066462	-2.713997772	0.001600612	0.013143168	RP11-459E5.1	processed_transcript	570
ENSG00000229728	-5.000105049	-2.635411409	0.000798442	0.007551089	RP11-314N13.3	antisense	480
ENSG00000087085	-5.007732684	-2.610946088	0.001011682	0.009124967	ACHE	protein_coding	1484
ENSG00000184357	-5.03554793	-2.797269125	0.002323016	0.017641119	HIST1H1B	protein_coding	681
ENSG00000233397	-5.08473669	-2.613902748	0.003301137	0.023064526	AC008063.3	lincRNA	619
ENSG00000267293	-5.104723164	-2.447992911	0.00030836	0.003506766	RP11-8H2.1	processed_pseudogene	1524
ENSG00000249180	-5.159737323	-2.797677921	0.002948513	0.021253308	CTC-506B8.1	antisense	565
ENSG00000070388	-5.201653699	-2.689257766	0.000526534	0.005366135	FGF22	protein_coding	1311
ENSG00000238097	-5.202841619	-2.885256264	0.000157693	0.002014593	RP11-513G11.3	lincRNA	1195
ENSG00000249947	-5.228663142	-2.493078186	0.00635259	0.038444121	XBP1P1	processed_pseudogene	652
ENSG00000197768	-5.251060348	-2.661809361	0.001385961	0.011796902	STPG3	protein_coding	1015
ENSG00000124602	-5.252238653	-2.638149185	0.000958874	0.008751571	UNC5CL	protein_coding	1620
ENSG00000265425	-5.293487773	-2.797844921	0.000240821	0.002840073	RP11-128D14.1	lincRNA	755
ENSG00000279943	-5.295468354	-2.742411426	0.000122521	0.001642263	FLJ38576	TEC	2459
ENSG00000231196	-5.359798625	-0.88161397	0.000194512	0.002379291	RP11-495P10.7	processed_transcript	1141.5
ENSG00000269904	-5.500763551	-2.71543063	0.001972582	0.015556053	MAP2K4P1	transcribed_processed_pseudogene	1867
ENSG00000104826	-5.506513027	-2.223674758	8.57E-05	0.001216166	LHB	protein_coding	514
ENSG00000273933	-5.54696766	-2.263929335	0.001857336	0.014833618	RP11-48O20.5	unprocessed_pseudogene	845
ENSG00000225173	-5.640688952	-2.297048489	0.000778623	0.007386398	XXbac-BPG308K3.5	lincRNA	709
ENSG00000187905	-5.711142604	-2.667682294	0.004855769	0.031270757	LRRC74B	protein_coding	2201
ENSG00000100341	-5.728487595	-2.640156407	0.004221722	0.028057651	PNPLA5	protein_coding	2197
ENSG00000250033	-5.736624079	-2.067770914	0.000147848	0.001906469	SLC7A11-AS1	processed_transcript	764
ENSG00000145506	-5.847850946	-0.569211553	4.52E-12	3.34E-10	NKD2	protein_coding	1060
ENSG00000215912	-6.107605215	-2.344223688	3.36E-06	7.45E-05	TTC34	protein_coding	5294.5
ENSG00000233806	-6.995615032	-1.502396982	5.54E-06	0.000115462	LINC01237	processed_transcript	573
ENSG00000183134	-8.740152173	-0.563581531	7.46E-13	6.15E-11	PTGDR2	protein_coding	2895
